# Global spatiotemporal patterns of bovine mastitis pathogens from 2000 to 2025: a meta-analysis for precision dairy health

**DOI:** 10.1186/s40104-026-01488-7

**Published:** 2026-09-10

**Authors:** Kai Yang, Guiqiu Hu, Zimo Pei, Meng Lan, Yu Cao, Xuanting Liu, Juxiong Liu, Dewei He, Wenjin Guo, Shoupeng Fu

**Affiliations:** 1 State Key Laboratory for Diagnosis and Treatment of Severe Zoonotic Infectious Diseases, Key Laboratory for Zoonosis Research of the Ministry of Education, Institute of Zoonosis, College of Veterinary Medicine, Jilin University, Changchun, 130062 China; 2 Jilin Provincial Key Laboratory of Nutrition and Functional Food and College of Food Science and Engineering, Jilin University, Changchun, 130062 China

**Keywords:** Bovine mastitis, Disease resistance breeding, Meta-analysis, Pathogen spectrum, Precision nutrition, Spatiotemporal analysis

## Abstract

**Background:**

Bovine mastitis is the most prevalent and economically damaging disease affecting dairy production worldwide. The disease can reduce milk yield and quality, shorten productive herd life, and compromise animal welfare and farm profitability. Although the spectra of responsible pathogens vary widely across regions, climates, and farming systems, long-term global spatiotemporal data that could guide mastitis resistance breeding and precision nutritional strategies remain limited. This meta-analysis aimed to characterize the spatiotemporal dynamics of bovine mastitis pathogens and to evaluate their associations with geography, climate, cattle breed, and husbandry systems.

**Results:**

A total of 137 studies published between 2000 and 2025 were included, covering 37 countries and 208,510 dairy cows. The pooled prevalence estimates and 95% confidence intervals for the five dominant pathogens were as follows: *Staphylococcus aureus*, 19.59% (95% CI: 17.00%–22.47%); *Streptococcus* spp., 17.29% (95% CI: 15.14%–19.66%); *Escherichia coli*, 13.86% (95% CI: 11.22%–17.00%); Coagulase-negative staphylococci, 15.49% (95% CI: 10.12%–22.99%); and *Klebsiella* spp., 9.92% (95% CI: 7.38%–13.20%). Overall, the pathogen spectrum clearly changed temporally over the 25-year period, with *Klebsiella* spp. exhibiting the most pronounced upward signal. Subgroup analyses suggested pathogen-specific variability across geographies and climates. The highest prevalence of *S. aureus* was observed in plateau climates and Africa. At the continent level, *Streptococcus* spp. were more prevalent in Europe and Asia, whereas climactically, they were more prevalent in plateau and tropical climates. Coagulase-negative staphylococci had the highest continental prevalence in Europe, while climate-specific estimates of its prevalence were relatively high in tropical and subtropical zones.

**Conclusions:**

This study provides long-term global evidence of the substantial temporal and geographic variability of the patterns of various bovine mastitis pathogens, including pathogen-specific evidence for geographic and climatic differences. These spatiotemporal patterns provide important etiological evidence for the targeted genetic improvement of mastitis resistance and pathogen-specific nutritional interventions. These findings provide essential etiological evidence for integrating multidisciplinary technologies including animal breeding, animal nutrition and farm health management to improve bovine mammary health, raise production efficiency and advance the sustainable development of dairy farming.

**Supplementary Information:**

The online version contains supplementary material available at https://doi.org/10.1186/s40104-026-01488-7.

## Introduction

Bovine mastitis is the most prevalent disease in the dairy industry worldwide and is widely recognized as one of the most economically important limiters of dairy production. The disease reduces milk yield and quality, increases culling rates, shortens the productive lifespan of dairy cows, and causes substantial economic losses to dairy farms while negatively affecting animal welfare and public health [1–3]. With the massive increases in dairy production in recent years, mastitis prevention and control have become key steps for improving production efficiency and encouraging healthy dairy farming. According to recent market surveys and forecasts, the global costs associated with bovine mastitis, including prevention, diagnosis, and treatment, is estimated to reach approximately $1.5 billion in 2025 and is projected to increase to $2.3 billion by 2032 [4]. Bovine mastitis can be classified as clinical or subclinical according to its clinical presentation. Clinical mastitis is further categorized into grades I–III and is characterized by local or systemic signs of varying intensity, including redness, swelling, heat, and pain of the udder, as well as changes in the physicochemical properties of the produced milk. Subclinical mastitis, in contrast, lacks obvious clinical signs but is associated with an elevated somatic cell count in the milk, making it particularly detrimental to dairy production [5].

Bovine mastitis can be caused by a wide range of pathogens [6], but the dominant pathogens vary substantially across regions, climates, husbandry systems, and management practices [7, 8]. Clarifying the temporal changes in the distributions of these pathogens is essential for developing targeted strategies for mastitis resistance breeding and nutritional regulation. In some regions, mastitis prevention and control still lack sufficient etiological support, limiting the precision and effectiveness of relevant intervention measures. Therefore, characterizing the global spatiotemporal changes in mastitis pathogens is important for guiding healthy dairy farming and delivering precision mastitis control.

Mastitis resistance is an important economic trait in dairy cattle breeding; in this context, differences in responsible pathogen spectra may directly affect the selection of resistance-related genes and the direction of breeding programs [9, 10]. Nutritional regulation is an important nonantibiotic strategy for improving mammary gland health and reducing the incidence of mastitis; to achieve these goals, however, different pathogens may require targeted nutritional interventions [11–14]. However, the lack of long-term global pathogen data limits the identification of mastitis resistance loci and the development of precision nutritional formulas for dairy cows.

Over the past 25 years, the global dairy industry has undergone substantial development, with the increasing adoption of automated milking systems and standardized health management [15]. These changes have led to greater requirements for mastitis prevention and control. However, previous studies have often been limited in scope, investigating only regional, short-term, and cross-sectional data, and a global, longitudinal analysis covering pathogen information over longer periods remains lacking.

In the present study, we collected global data on bovine mastitis pathogens from 137 studies covering 37 countries and 208,510 dairy cows published from 2000 to 2025. The prevalence of 15 pathogens was analyzed, and differences across continents, countries/regions, climates, sampling periods, cow breeds, and farming systems were evaluated. A random-effects meta-analysis model was used to characterize the temporal and spatial distributions of the pathogens and the factors that potentially influence these distributions. The aim was to clarify the global spatiotemporal pattern of mastitis pathogens and to provide a rational basis for developing mastitis resistance breeding, precision nutrition, and healthy dairy farming strategies.

## Materials and methods

### Search strategy

This study was conducted in accordance with the 2020 PRISMA statement [16] (the checklist can be found in Table S1). To identify eligible studies related to the pathogen spectrum of dairy cow mastitis, the PubMed, Web of Science, and Embase databases were searched using predefined search terms (Table S2). The search was restricted to English-language publications, and no restrictions on publication type were imposed.

### Screening process

Two authors independently screened the titles and abstracts of the studies retrieved from the database searches. The articles considered eligible on this basis were then evaluated according to predetermined inclusion and exclusion criteria (Table S4). At this stage, eligible studies were epidemiological or original research articles that involved sampling after 2000, had clear research objectives, and reported their detection methods and results for pathogenic microorganisms associated with dairy cow mastitis. The exclusion criteria were duplicate publications; non-English articles; reviews; meta-analyses; studies with unclear pathogen information, unavailable data or missing geographic information, unavailable full text, or sampling performed before 2000; case reports; and laboratory-based molecular mechanism studies. The screening decisions were finalized through weekly discussions among the authors. The final included studies are listed in Table S3.

### Data extraction

A predesigned data-extraction template was used to ensure consistency. The extracted variables included first author, title, study retrieval code and database, publication year, sampling year, sampling country, detection method, breed purity, and farming output. Because most studies did not explicitly report the relevant climate zone or continent, these variables were determined according to the reported sampling locations. For studies in which the sampling location was reported only at the national level and spanned multiple climate zones, climate data were not included.

### Quality assessment

Two authors independently assessed the risk of bias according to the methods in previous epidemiological meta-analyses and the Strengthening the Reporting of Observational Studies in Epidemiology (STROBE) guidelines [17, 18]. Briefly, the assessment included sample selection, sample size, outcome assessment, and analytical method (Tables S5 and S6). To further evaluate study quality, a checklist developed on the basis of previous studies [18, 19] was used. The checklist contained 10 items, each scored as 1 point for “Yes” or 0 points for “No”. The final score, *X*, was calculated as the sum of the 10 item scores divided by 10, according to which the study was classified as high quality (*X* ≥ 0.78), moderate quality (0.44 < *X* < 0.78), or low quality (*X* ≤ 0.44). In accordance with the Cochrane Handbook and current statistical recommendations for meta-analyses, quality scores were not directly used as weights in the quantitative synthesis; instead, they were used only as a reference for evaluating the quality of the included study (Tables S7 and S8). The results of the quality assessment was also confirmed through weekly communication among all the authors.

### Statistical analysis

All analyses were performed using R software (version 4.5.2) and RStudio (version 2025.09.2 + 418). Publication trends for pathogen-specific mastitis studies were analyzed using generalized linear models constructed with a negative binomial distribution to account for count data and possible overdispersion. The binom package was used to calculate 95% confidence intervals (CIs) for pathogen-positivity rates. Given the substantial heterogeneity arising from the broad temporal range (2000–2025) and wide geographic distribution of the included studies, meta-analyses of the five major pathogen categories identified in the broader meta-analysis were conducted using a random-effects model in the meta package. Pooled prevalence was estimated using an inverse-variance random-effects model with logit transformation (sm = “PLOGIT”). The DerSimonian‒Laird estimator was used as the primary estimator of τ^2^, while REML, Paule‒Mandel, and Hartung‒Knapp-adjusted models were used in the sensitivity analyses [20–24]. Heterogeneity was assessed using the Chi-square test (Cochrane’s Q statistic) and the *I*^2^ statistic; for the latter, values of 25%, 50%, and 75% were considered indicative of low, moderate, and high heterogeneity, respectively, and *P* < 0.05 was considered indicative of statistical significance. Heterogeneity and potential publication bias were evaluated using forest plots, funnel plots, Egger’s test, and Begg’s test. A *P* < 0.05 in either Egger’s test or Begg’s test was considered indicative of potential publication bias. Temporal trends in pathogen-positivity rates from 2000 to 2025 were explored using meta-regression or descriptive trend analysis, depending on the availability and continuity of the data. Subgroup analyses based on continent, country/region, climate, breed purity, and farming system were performed to clarify the effects of genetic and environmental factors on the prevalence of the pathogens. Result stability was evaluated using leave-one-out sensitivity analysis [25]. Analyses restricted to high-quality studies were also conducted [26].

## Results

### Study selection

**Fig. 1:**
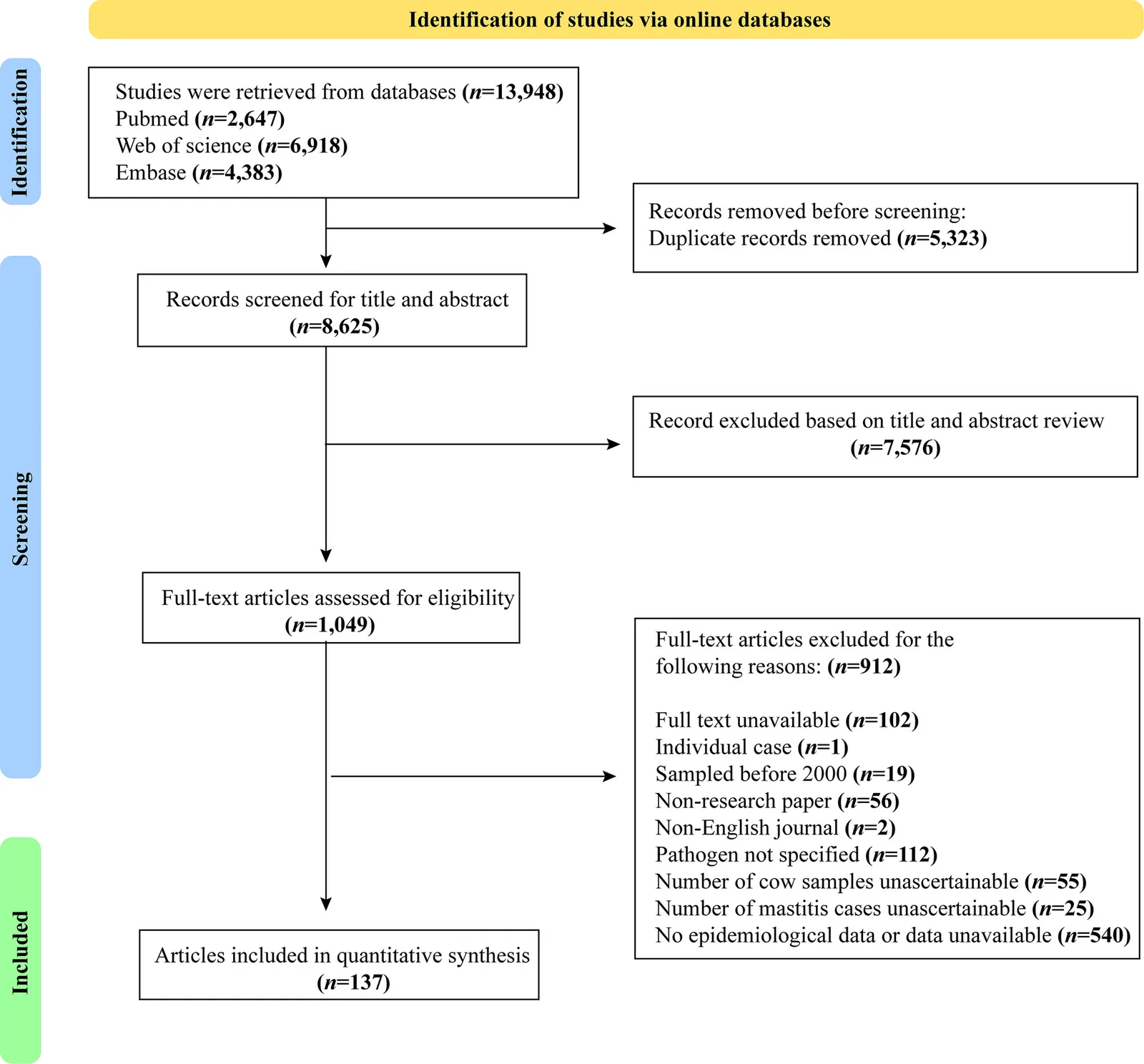
Flow chart of study selection

The initial searches across all three databases yielded 13,948 studies. After duplicate records were removed, 8,625 articles remained. After the titles and abstracts were read, 7,576 of these articles were excluded. A further 912 studies were excluded if they had unavailable full text (102), were case reports (1), had a noncompliant sampling time (19), were nonresearch articles (56), were published in a language other than English (2), did not define the pathogens involved (112), did not report the number of sampled dairy cows (55), did not report the number of mastitis cases (25), and had missing or inaccessible epidemiological data (540). Ultimately, 137 studies were eligible for the epidemiological meta-analysis and were included in the final analysis (Fig. 1 and Table S3).

### Characteristics of the included studies

**Fig. 2:**
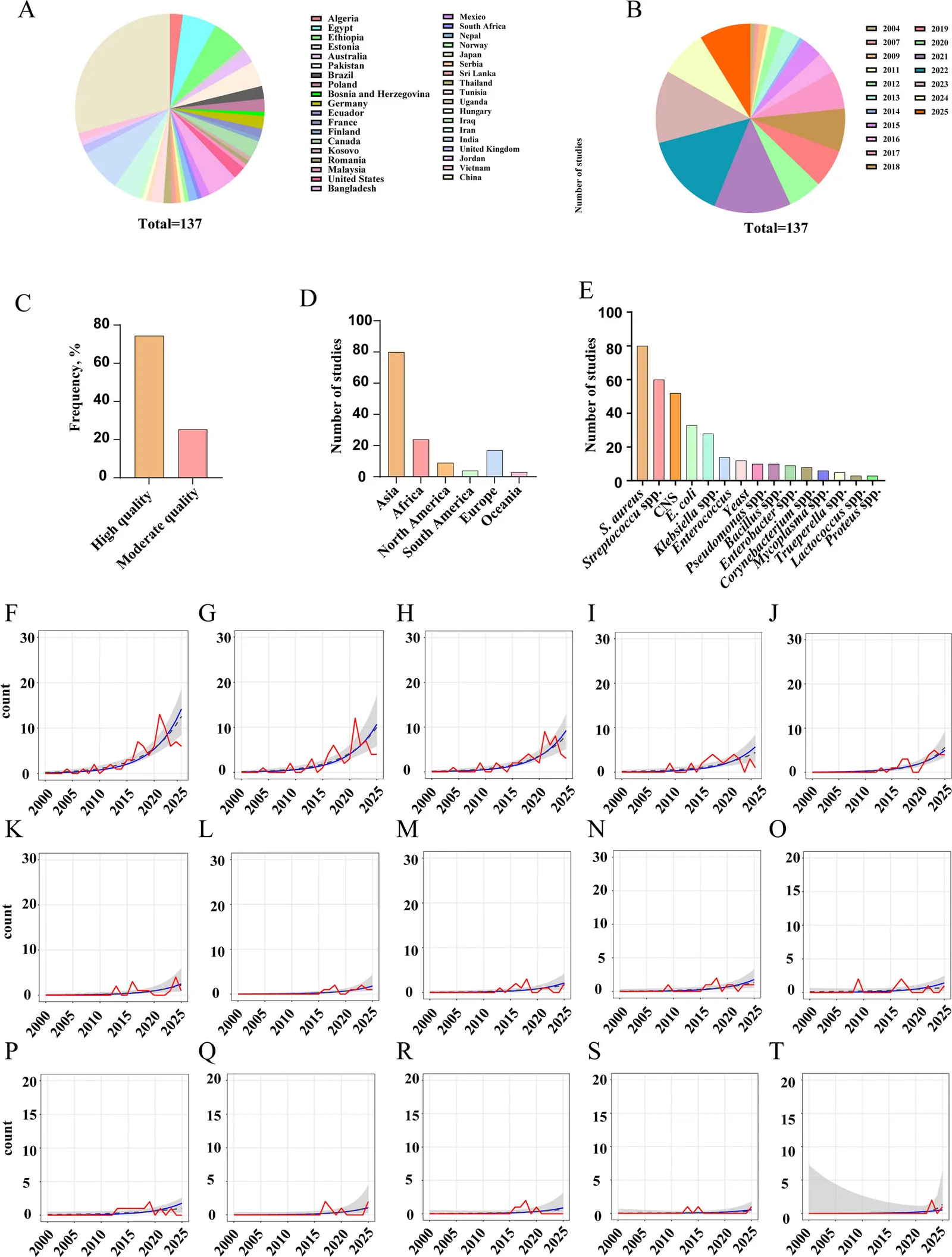
Characteristics of the included studies. **A** Distribution of the included studies by country. **B** Distribution of publication dates of the included studies. **C** Quality assessment of the included studies. **D** Distribution of the included studies by continent. **E** Number of studies on the included pathogens. **F**–**T** Annual changes in the volume of published studies on the epidemiology of pathogens causing bovine mastitis. The dashed line and shaded area represent the regression line fitted using a generalized linear model with a negative binomial distribution and a log link function. The blue line indicates the expected trend based on total publication volume, which is used to determine whether the publication rate for a specific pathogen is faster or slower than the overall publication rate for bovine mastitis

The 137 included studies were conducted across 37 countries, indicating a relatively broad global coverage of studies on bovine mastitis pathogens (Fig. 2A). The number of eligible studies increased after 2010; 56.9% were published during the most recent five years, and 29.9% were published between 2016 and 2020 (Fig. 2B). Most studies were conducted in Asia, whereas South America and Oceania were underrepresented (Fig. 2D). The quality assessment revealed that 74.5% of the included studies were of high quality (Fig. 2C). In terms of pathogen-specific research volume, *S. aureus* was the most frequently investigated pathogen category, followed by *Streptococcus* spp., *E. coli*, coagulase-negative staphylococci (CNS), and *Klebsiella* spp. (Fig. 2E).

Longitudinal analysis of the publication trends from 2000 to 2025 revealed a strong correspondence between research on specific pathogens and the overall growth of the dairy mastitis field. The total number of studies on the epidemiology of major mastitis-causing pathogens has steadily increased. The global publication growth rate (shown as a blue line in the relevant figures) was used as a benchmark to evaluate whether research interest in each specific pathogen (shown as a red line in the relevant figures) was consistent with, exceeded, or lagged behind the overall growth of bovine mastitis research (Fig. 2F–T).

For the five major pathogens (Fig. 2F–J), which represented the dominant pathogen categories, the publication trajectories were nearly aligned with the expected baseline, indicating that the research output for these pathogens increased proportionally with that of the field as a whole. Some minor pathogens (Fig. 2K–N) were associated with substantially lower publication volumes and were categorized as low-attention or sporadically reported pathogens; although their publication trends generally aligned with expectations, they appeared to fluctuate more or were relatively flat because of their small baseline size. Other minor pathogens (Fig. 2O–T) consistently had low absolute publication numbers, generally remaining near the baseline, and were associated with wider confidence intervals because of the sparsity of the relevant data.

### Global pooled prevalence estimates, bias assessments, and robustness analyses for pathogens that cause mastitis in dairy cows

**Fig. 3:**
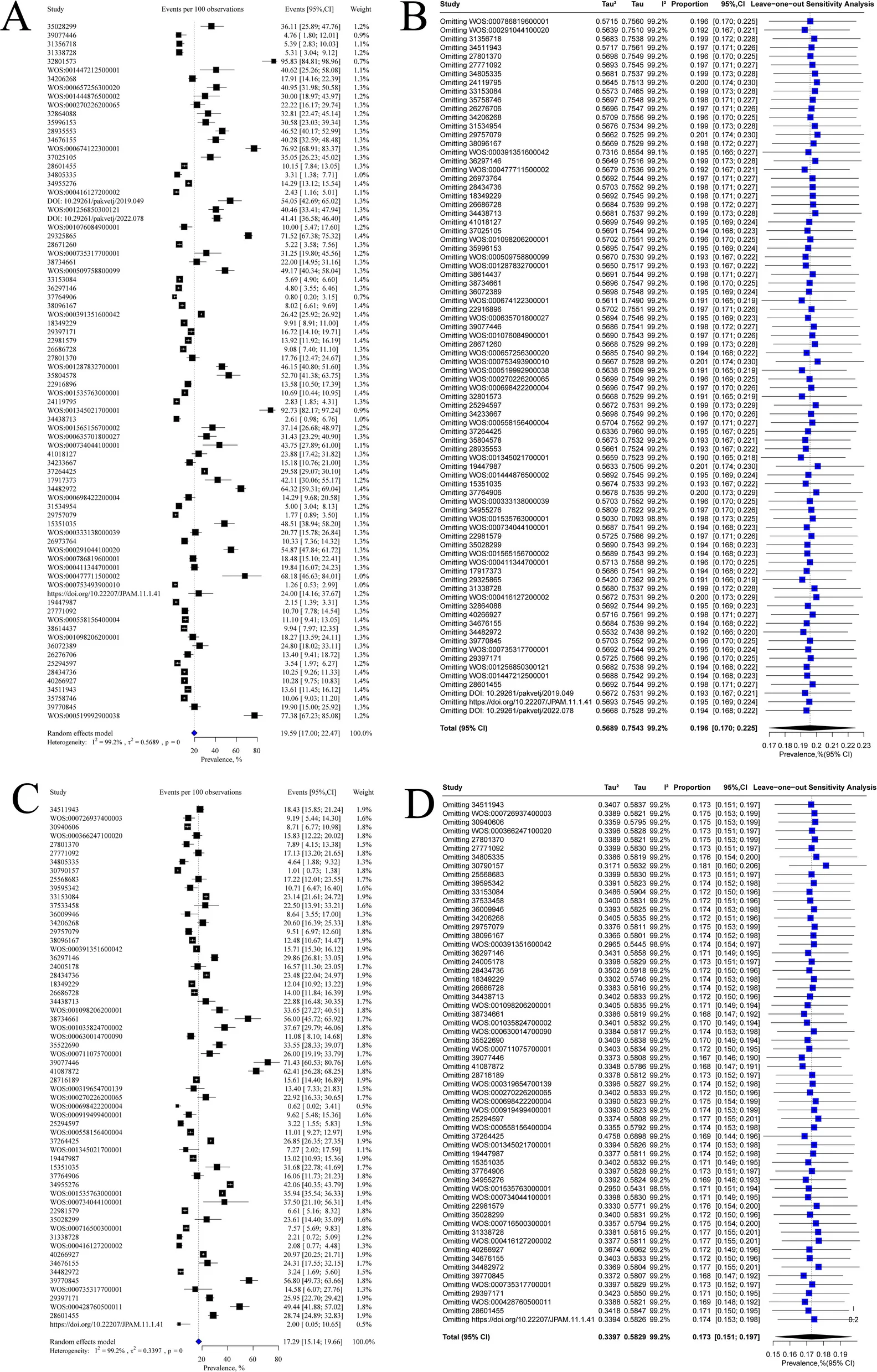
Meta-analysis of major pathogens causing dairy cow mastitis (2000–2025). **A** and **B** Forest plot of the results of the meta-analysis and leave-one-out sensitivity analysis, respectively, for *Staphylococcus aureus.* **C** and **D** Forest plot of the results of the meta-analysis and leave-one-out sensitivity analysis, respectively, for *Streptococcus* spp.

**Fig. 4:**
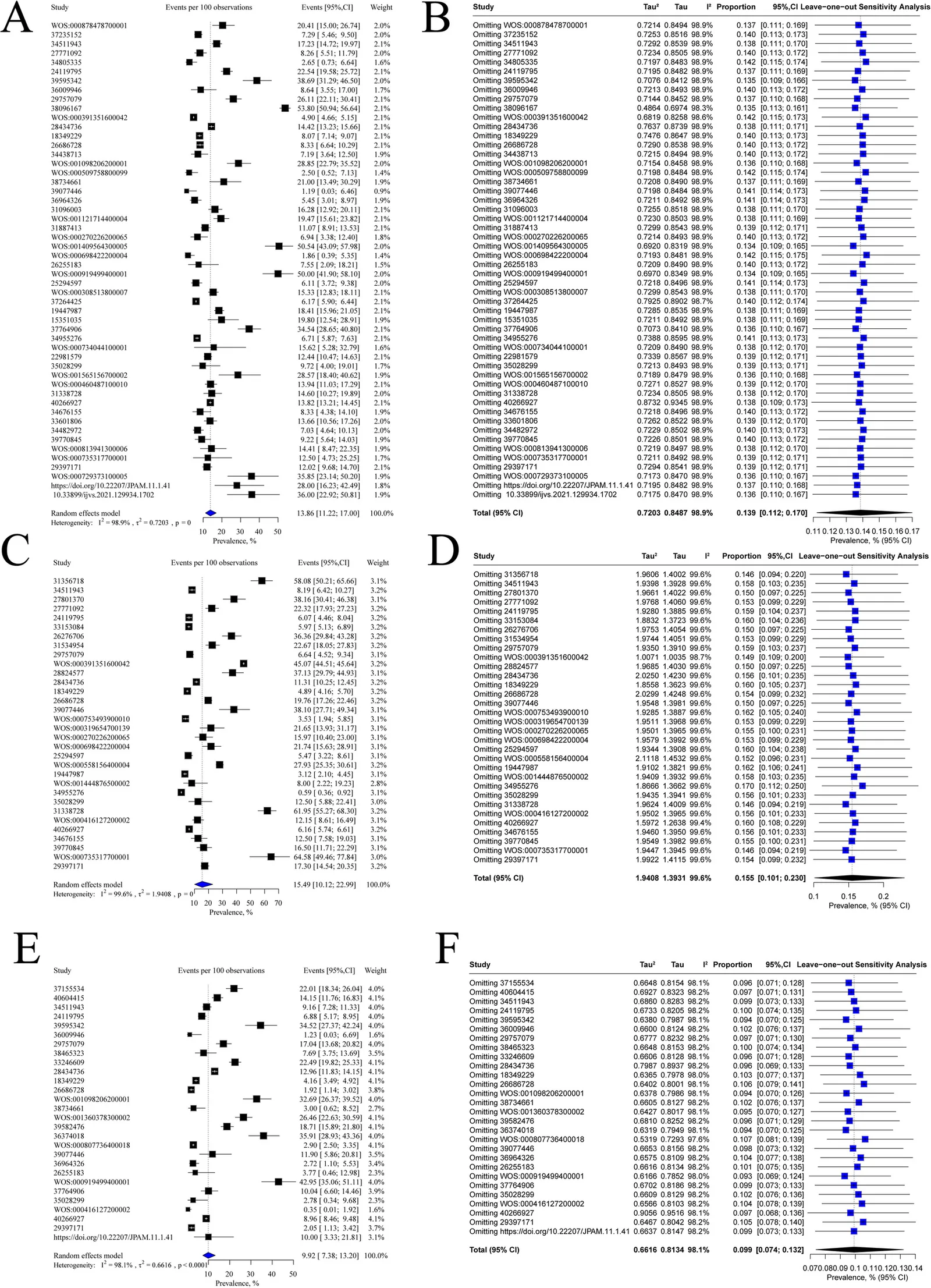
Meta-analysis of major pathogens causing dairy cow mastitis (2000–2025). **A** and **B** Forest plot of the results of the meta-analysis and leave-one-out sensitivity analysis, respectively, for *Escherichia coli.* **C** and **D** Forest plot of the results of the meta-analysis and leave-one-out sensitivity analysis, respectively, for coagulase-negative staphylococci*.* **E** and **F** Forest plot of the results of the meta-analysis and leave-one-out sensitivity analysis, respectively, for *Klebsiella* spp.

Random effects pooled analysis of the studies conducted between 2000 and 2025 revealed that the prevalence of bovine mastitis caused by the five dominant pathogen categories was 19.59% for *S. aureus* (95% CI: 17.00%−22.47%), 17.29% for *Streptococcus* spp. (95% CI: 15.14%−19.66%), 13.86% for *E. coli* (95% CI: 11.22%−17.00%), 15.49% for CNS (95% CI: 10.12%−22.99%), and 9.92% for *Klebsiella* spp. (95% CI: 7.38%−13.20%) (Figs. 3A, 3C, 4A, 4C, and 4E; Tables 1, 2, 3, 4, and 5). Substantial heterogeneity was observed across all five pathogen categories, with *I*^2^ values ranging from 98.1% to 99.6% (all *P* < 0.001). The wide confidence intervals indicated that the true prevalence may vary considerably across future or local study settings, reflecting differences in geographic region, sampling strategy, diagnostic method, and host population characteristics.

**Table 1:** *S. aureus* subgroup information

Variable (Subgroups)	Number of study (*n*)	Number sampled (*n*)	Number of positive (*n*)	Rate of positive, %	95% CI lower, %	95% CI upper, %	Heterogeneity Chi-squared (*χ*^2^)	*I*^2^, %	*P*-value for heterogeneity
Continent							5,353.88	99.9	< 0.0001
North America	8	63,659	6,815	11.72	9.52	14.34	125.04	94.4	< 0.0001
Oceania	3	3,658	472	5.16	1.29	18.5	35.75	94.4	< 0.0001
Africa	19	2,910	673	27.04	18.19	38.18	443.33	95.9	< 0.0001
South America	1	498	26	5.22	3.59	7.54	-	-	-
Europe	14	67,616	17,535	16.54	13.35	20.31	1,322.29	99.3	< 0.0001
Asia	35	26,866	3,882	19.59	17.00	22.47	2,488.87	98.6	< 0.0001
Country							9,514.08	99.7	< 0.0001
United States	3	57,774	6,130	7.8	4.17	14.15	43.96	95.5	< 0.0001
Canada	4	5,780	652	12.07	9.19	15.69	36.92	91.9	< 0.0001
Mexico	1	105	33	31.43	23.34	40.83	-	-	-
Australia	3	3,658	472	5.16	1.29	18.5	35.75	94.4	< 0.0001
Algeria	2	393	21	5.34	3.51	8.06	0	0	0.9724
Egypt	5	570	177	44.61	24.7	66.42	59.42	93.3	< 0.0001
Ethiopia	8	1,002	415	39.9	29.09	51.8	88.53	92.1	< 0.0001
Tunisia	3	913	46	5.25	1.61	15.8	30.33	93.4	< 0.0001
South Africa	1	32	14	43.75	28.17	60.67	-	-	-
Brazil	1	498	26	5.22	3.59	7.54	-	-	-
Kosovo	1	152	27	17.76	12.50	24.61	-	-	-
Germany	3	3,986	207	4.48	2.9	6.86	8.64	76.8	0.0133
France	1	1,210	97	8.02	6.62	9.68	-	-	-
Finland	1	29,969	7,917	26.42	25.92	26.92	-	-	-
Bosnia and Herzegovina	1	120	59	49.17	40.39	58	-	-	-
Romania	1	325	150	46.15	40.81	51.59	-	-	-
Poland	2	148	37	25.59	17.82	35.28	1.47	31.8	0.2258
Norway	1	30,154	8,919	29.58	29.07	30.10	-	-	-
United Kingdom	1	929	20	2.15	1.40	3.30	-	-	-
Hungary	1	101	49	48.51	39	58.14	-	-	-
Estonia	1	522	53	10.15	7.85	13.04	-	-	-
India	6	1,465	292	22.86	10.82	41.99	164.12	97	< 0.0001
China	13	22,480	2,436	14.42	11.75	17.57	245.89	95.1	< 0.0001
Iran	2	507	74	14.86	7.23	28.09	10.34	90.3	0.0013
Bangladesh	3	278	81	36.94	3.34	90.85	72.21	97.2	< 0.0001
Nepal	2	325	61	19.15	12.03	29.1	3.85	74	0.0497
Thailand	1	84	4	4.76	1.87	11.61	-	-	-
Pakistan	5	1,226	633	41.79	24.65	61.17	143.94	97.2	< 0.0001
Malaysia	1	74	39	52.70	41.48	63.66	-	-	-
Japan	1	57	24	42.11	30.19	55.02	-	-	-
Sri Lanka	1	370	238	64.32	59.32	69.04	-	-	-
Climate							35.75	94.4	< 0.0001
Plateau climate	6	808	294	35.00	27.85	42.91	24.72	79.8	0.0002
Tropical climate	12	2,458	721	31.59	18.54	48.36	456.72	97.6	< 0.0001
Temperate climate	29	90,826	19,987	14.87	12.03	18.24	3,971.28	99.3	< 0.0001
Subtropical climate	22	61,239	7,171	20.45	12.73	31.17	1935.1	98.9	< 0.0001
Breeding type							595.95	99.8	< 0.0001
Purebred dairy cows	9	34,315	8,508	21.95	14.32	32.12	531.61	98.5	< 0.0001
Crossbred dairy cows	12	7,589	874	15.1	9.77	22.61	381.75	97.1	< 0.0001
Production system							7.07	85.8	0.0079
Intensive	25	89,692	19,977	22	14.3	32.1	3,853.48	99.4	< 0.0001
Extensive	13	2055	407	27.3	17.8	39.4	220.81	94.6	< 0.0001

**Table 2:** *Streptococcus* spp. subgroup information

Variable (Subgroups)	Number of study (*n*)	Number sampled (*n*)	Number of positive (*n*)	Rate of positive, %	95% CI lower, %	95% CI upper, %	Heterogeneity Chi-squared (*χ*^2^)	*I*^2^, %	*P*-value for heterogeneity
Continent							4,211.98	99.9	< 0.0001
North America	5	62,430	21,112	16.63	8.05	31.22	1,086.33	99.6	< 0.0001
Oceania	3	3,658	1,367	8.51	0.76	53.05	120.12	98.3	< 0.0001
Africa	9	1663	228	14.58	9.43	21.88	76.72	89.6	< 0.0001
Europe	14	70,589	14,822	20.07	16.34	24.40	1,405.56	99.1	< 0.0001
Asia	29	27,172	4,779	17.26	13.56	21.72	1,375.14	98	< 0.0001
Country							6,181.9	99.6	< 0.0001
Ethiopia	3	360	85	23.62	19.51	28.28	0.08	0	0.9622
Algeria	1	226	5	2.21	0.95	5.07	-	-	-
Egypt	1	335	69	20.60	16.61	25.25	-	-	-
Estonia	1	522	150	28.74	25.02	32.76	-	-	-
Australia	3	3,658	1,367	8.51	0.86	53.05	120.12	98.3	< 0.0001
Poland	2	148	63	32.48	6.3	77.49	19.42	94.8	< 0.0001
Germany	3	3,986	962	23.13	17.8	29.49	25.36	92.1	< 0.0001
France	1	1,210	151	12.48	10.74	14.46	-	-	-
Finland	1	29,969	4,707	15.71	15.30	16.12	-	-	-
Canada	4	5,780	754	13.35	8.15	21.10	130.34	97.7	< 0.0001
Kosovo	1	152	12	7.89	4.57	13.29	-	-	-
United States	1	56,650	20,358	35.94	35.54	36.33	-	-	-
Bangladesh	4	434	112	22.43	13.51	34.87	19.28	84.4	0.0002
South Africa	1	32	12	37.50	22.93	54.75	-	-	-
Norway	1	30,154	8,096	26.85	26.35	27.35	-	-	-
Serbia	1	81	7	8.64	4.25	16.78	-	-	-
Sri Lanka	1	370	12	3.24	1.86	5.58	-	-	-
Thailand	2	262	148	64.54	37.96	79.36	10.91	90.8	0.001
Tunisia	2	613	44	2.96	0.20	32.19	7.72	87	0.0055
Uganda	1	97	13	13.40	8	21.59	-	-	-
Hungary	1	101	32	31.68	23.42	41.29	-	-	-
Iran	1	180	31	17.22	12.41	23.41	-	-	-
India	2	3,995	41	1.03	0.76	1.40	0.45	0	0.5003
United Kingdom	2	4,266	642	14.48	12.13	17.20	3.8	73.7	0.0514
China	19	21,931	4,435	17.92	14.31	22.21	706.96	97.5	< 0.0001
Climate							1,375.14	98	< 0.0001
Plateau climate	3	360	85	23.6	19.5	28.3	0.08	0	0.9622
Tropical climate	10	1,570	373	22.69	12.64	37.31	200.8	96	< 0.0001
Temperate climate	30	91,501	18,554	17.81	15.39	20.52	2,214.85	98.7	< 0.0001
Subtropical climate	12	62,918	20,692	7.73	3.57	15.91	1,009.12	98.9	< 0.0001
Breeding type							44.54	97.8	< 0.0001
Purebred dairy cows	5	34,322	5,374	19.88	13.75	27.86	232.48	98.3	< 0.0001
Crossbred dairy cows	10	6,243	1,189	20.51	13.21	30.43	335.29	97.3	< 0.0001
Production system							5.33	81.2	0.021
Intensive	21	89,138	18,034	16.62	13.87	19.79	2,206.84	99.1	< 0.0001
Extensive	7	1,293	228	14.33	5.53	32.25	200.64	97	< 0.0001

**Table 3:** *E. coli* subgroup information

Variable (Subgroups)	Number of study (*n*)	Number sampled (*n*)	Number of positive (*n*)	Rate of positive, %	95% CI lower, %	95% CI upper, %	Heterogeneity Chi-squared (*χ*^2^)	*I*^2^, %	*P*-value for heterogeneity
Continent							1,297.5	99.6	< 0.0001
North America	6	6,574	711	11.42	7.67	16.69	131.04	96.2	< 0.0001
Oceania	2	3,370	220	4.79	1.97	11.20	3.61	72.3	0.0574
Africa	8	1663	247	11.05	7.02	16.99	66.29	89.4	< 0.0001
South America	1	257	14	5.45	3.27	8.93	-	-	-
Europe	10	62,961	4,292	14.26	7.53	25.38	2,890.56	99.7	< 0.0001
Asia	25	22,366	3,271	17.49	14.53	20.92	534.06	95.5	< 0.0001
Country							5,353.88	98.9	< 0.0001
Ethiopia	3	360	29	8.12	5.70	11.44	0.52	0	0.7704
Thailand	1	84	1	1.19	0.21	6.44	-	-	-
Algeria	1	226	33	14.60	10.59	19.80	-	-	-
Egypt	1	432	59	13.66	10.74	17.22	-	-	-
Australia	2	3,370	220	4.79	1.97	11.2	3.61	72.3	0.0574
Pakistan	1	111	16	14.41	9.07	22.14	-	-	-
Brazil	1	257	14	5.45	3.27	8.93	-	-	-
Poland	2	148	27	17.79	10.88	27.73	1.54	35	0.2149
Bosnia and Herzegovina	1	120	3	2.50	0.85	7.09	-	-	-
Germany	1	249	86	34.54	28.91	40.64	-	-	-
France	1	1,210	651	53.80	50.98	56.59	-	-	-
Finland	1	29,969	1,468	4.90	4.66	5.15	-	-	-
Canada	4	5,780	540	10.00	7.84	12.67	24.11	87.6	< 0.0001
United States	1	741	167	22.54	19.67	25.68	-	-	-
Bangladesh	4	460	143	27.11	11.57	51.39	58.12	94.8	< 0.0001
Mexico	1	53	4	7.55	2.97	17.86	-	-	-
South Africa	1	32	5	15.62	6.86	31.75	-	-	-
Norway	1	30,154	1859	6.17	5.90	6.44	-	-	-
Serbia	1	81	7	8.64	4.25	16.78	-	-	-
Sri Lanka	1	370	26	7.03	4.84	10.10	-	-	-
Tunisia	2	613	121	7.97	0.49	60.27	24.34	95.9	< 0.0001
Hungary	1	101	20	19.80	13.20	28.62	-	-	-
Iraq	1	50	18	36	24.14	49.86	-	-	-
Iran	2	932	140	15.06	12.90	17.51	0.99	0	0.3204
India	1	50	14	28	17.47	41.67	-	-	-
United Kingdom	1	929	171	18.41	16.05	21.03	-	-	-
Jordan	1	380	74	19.47	15.81	23.75	-	-	-
Vietnam	1	686	50	7.29	5.57	9.48	-	-	-
China	12	19,243	2,789	17.11	13.70	21.16	275.94	96	< 0.0001
Climate							339.68	99.1	< 0.0001
Plateau climate	3	360	29	8.12	5.70	11.44	0.52	0	0.7704
Tropical climate	8	1603	146	12.12	6.26	22.17	96.48	92.7	< 0.0001
Temperate climate	24	83,695	7,026	13.75	9.97	18.67	3,755.05	99.4	< 0.0001
Subtropical climate	9	1849	391	19.19	12.13	29.02	218.28	95.9	< 0.0001
Breeding type							852.12	99.9	< 0.0001
Purebred dairy cows	5	33,922	1876	12.30	6.51	22.04	371.41	98.9	< 0.0001
Crossbred dairy cows	12	7,564	1,185	16.13	11.80	21.67	258.53	95.7	< 0.0001
Production system							195.39	99.5	< 0.0001
Intensive	15	81,421	6,910	16.39	11.05	23.63	3,704.27	99.6	< 0.0001
Extensive	12	1831	331	12.53	7.48	20.24	177.95	93.8	< 0.0001

**Table 4:** CNS subgroup information

Variable (Subgroups)	Number of study (*n*)	Number sampled (*n*)	Number of positive (*n*)	Rate of positive, %	95% CI lower, %	95% CI upper, %	Heterogeneity Chi-squared (*χ*^2^)	*I*^2^, %	*P*-value for heterogeneity
Continent							6,555.14	99.9	< 0.0001
North America	4	5,508	502	10.29	4.70	21.05	228.03	98.9	< 0.0001
Oceania	2	3,507	54	2.79	0.13	38.61	115.98	99.1	< 0.0001
Africa	10	1813	445	20.82	11.29	35.20	306.6	97.1	< 0.0001
Europe	5	33,981	13,798	22.07	5.98	55.76	1,330.73	99.7	< 0.0001
Asia	11	19,025	1813	15.49	9.62	24.00	924.05	98.9	< 0.0001
Country							8,851.17	99.8	< 0.0001
Ethiopia	3	360	50	13.97	10.75	17.97	0.87	0	0.6482
Thailand	1	84	32	38.10	28.45	48.78	-	-	-
Algeria	2	393	237	60.29	55.36	65.02	0.6	0	0.4392
Egypt	1	50	4	8	3.15	18.84	-	-	-
Australia	2	3,507	54	2.79	0.13	38.61	115.98	99.1	< 0.0001
Poland	1	48	31	64.58	50.44	76.57	-	-	-
Germany	1	2,883	172	5.97	5.16	6.89	-	-	-
Finland	1	29,969	13,508	45.07	44.51	45.64	-	-	-
Canada	3	4,767	457	12.15	4.76	27.70	208.67	99	< 0.0001
Kosovo	1	152	58	38.16	30.82	46.08	-	-	-
United States	1	741	45	6.07	4.57	8.03	-	-	-
Tunisia	3	913	133	15.28	7.00	30.18	40.61	95.1	< 0.0001
Uganda	1	97	21	21.65	14.62	30.84	-	-	-
India	2	564	76	12.91	0.96	69.36	77.64	98.7	< 0.0001
United Kingdom	1	929	29	3.12	2.18	4.45	-	-	-
China	8	18,377	1705	14.21	8.33	23.19	775.11	99.1	< 0.0001
Climate							144.79	97.9	< 0.0001
Plateau climate	3	360	50	13.97	10.75	17.97	0.87	0	0.6482
Tropical climate	4	745	129	19.94	7.24	44.27	86.73	96.5	< 0.0001
Temperate climate	13	52,390	15,197	16.45	7.59	32.08	6,723.65	99.8	< 0.0001
Subtropical climate	7	1947	401	19.35	9.20	36.23	289.79	97.9	< 0.0001
Breeding type							1,477.93	99.9	< 0.0001
Purebred dairy cows	5	33,833	13,840	21.18	6.72	50.05	1,208.62	99.7	< 0.0001
Crossbred dairy cows	6	5,251	541	16.12	8.19	29.29	188	97.3	< 0.0001
Production system							40.49	97.5	< 0.0001
Intensive	9	50,561	15,087	15.61	5.33	30.47	6,258.24	99.9	< 0.0001
Extensive	6	1,261	271	19.21	7.70	40.39	221.19	97.7	< 0.0001

**Table 5:** *Klebsiella* spp. subgroup information

Variable (Subgroups)	Number of study (*n*)	Number sampled (*n*)	Number of positive (*n*)	Rate of positive, %	95% CI lower, %	95% CI upper, %	Heterogeneity Chi-squared (*χ*^2^)	*I*^2^, %	*P*-value for heterogeneity
Continent							249.25	98	< 0.0001
North America	5	5,561	216	3.44	2.13	5.52	31.89	87.5	< 0.0001
Oceania	1	288	1	0.35	0.01	1.92	-	-	-
Africa	2	524	79	8.00	1.26	37.14	7.34	86.4	0.0067
South America	1	257	7	2.72	1.33	5.51	-	-	-
Europe	3	430	29	4.29	1.24	13.77	8.21	75.6	0.0165
Asia	16	26,791	2,727	16.87	12.04	23.14	1,119.92	98.7	< 0.0001
Country							343.7	96.2	< 0.0001
Ethiopia	1	72	2	2.78	0.77	9.57	-	-	-
Thailand	1	84	10	11.90	6.60	20.54	-	-	-
Australia	1	288	1	0.35	0.06	1.94	-	-	-
Brazil	1	257	7	2.72	1.33	5.51	-	-	-
Poland	1	100	3	3	1.03	8.45	-	-	-
Germany	1	249	25	10.04	6.89	14.40	-	-	-
Canada	3	4,767	163	2.64	1.46	4.71	14.74	86.4	0.0006
United States	1	741	51	6.88	5.27	8.94	-	-	-
Mexico	1	53	2	3.77	1.04	12.75	-	-	-
Serbia	1	81	1	1.23	0.22	6.67	-	-	-
Tunisia	1	452	77	17.04	13.85	20.78	-	-	-
India	2	180	15	8.39	5.12	13.45	0.25	0	0.6168
Vietnam	1	468	103	22.01	18.49	25.98	-	-	-
China	12	26,059	2,599	18.38	12.50	26.19	1,073.53	99	< 0.0001
Climate							189.72	98.4	< 0.0001
Plateau climate	1	72	2	2.78	0.77	9.57	-	-	-
Tropical climate	6	1,042	137	8.35	3.85	17.16	50.85	90.2	< 0.0001
Temperate climate	14	27,482	2,202	9.03	5.96	13.46	941.25	98.6	< 0.0001
Subtropical climate	4	1,104	213	19.19	9.04	36.18	66.47	95.5	< 0.0001
Breeding type							25.37	96.1	< 0.0001
Purebred dairy cows	2	3,317	189	13.10	1.29	63.53	181.81	99.4	< 0.0001
Crossbred dairy cows	10	12,389	1,035	8.27	4.67	14.24	559.59	98.4	< 0.0001
Production system							11.28	91.1	0.0008
Intensive	16	27,569	2,700	13.18	9.20	18.52	12.11	83.5	0.0023
Extensive	3	577	81	6.53	1.59	23.22	1,087.82	98.6	< 0.0001

The results of Egger’s tests and Begg’s tests, together with visual inspection of the funnel plots, suggested potential publication bias or small-study effects for some pathogen categories (Figs. S1–S5 and Table S10). Specifically, no significant publication bias was detected for *S. aureus* by either Egger’s test or Begg’s test (*P* > 0.05), although its funnel plot contains some visual asymmetry. For *Streptococcus* spp., *E. coli*, and CNS, the significance of the results of Egger’s and/or Begg’s test and the asymmetric funnel plots indicated possible publication bias or small-study effects.

To evaluate the stability of the pooled estimates, leave-one-out sensitivity analysis was conducted by excluding one study at a time (Figs. 3B, 3D, 4B, 4D, and 4F). After the removal of the individual studies, the recalculated pooled prevalence estimates for all five dominant pathogen categories remained close to the original estimates; the nearly vertical distribution of the estimates in the sensitivity plots indicated that no single study disproportionately influenced the pooled results, supporting the robustness of the global summary estimates. To further evaluate the robustness of the pooled estimates under conditions of substantial heterogeneity, alternative random-effects specifications were compared for the five dominant pathogen categories (Table S11). Across the DerSimonian–Laird models, REML models, Paule–Mandel models, and REML models with Hartung–Knapp adjustment, the pooled prevalence estimates remained broadly consistent. Specifically, the estimates ranged from 19.52% to 19.59% for *S. aureus*; from 16.42% to 17.29% for *Streptococcus* spp.; from 13.81% to 13.86% for *E. coli*; from 15.49% to 15.50% for CNS; and from 9.32% to 9.92% for *Klebsiella* spp. Although the confidence intervals widened under some alternative specifications, particularly after Hartung–Knapp adjustment, the overall ranking and interpretation of the dominant pathogen categories did not change markedly.

Sensitivity analyses restricted to high-quality studies yielded results similar to those obtained from the broader analysis (Table S12). After the analysis was restricted to high-quality studies, the pooled prevalence estimates were 20.92% for *S. aureus*, 18.53% for *Streptococcus* spp., 12.67% for *E. coli*, 16.31% for CNS, and 10.80% for *Klebsiella* spp. These estimates are similar to the corresponding estimates from the full dataset, indicating that the main findings were not dominated by lower-quality studies. However, the heterogeneity remained substantial in the high-quality-only analyses, with *I*^2^ values ranging from 97.7% to 98.9%, suggesting that between-study variability was attributable mainly to true differences in geographic region, diagnostic method, study population, and production system rather than to study quality alone.

### Global prevalence and spatiotemporal variability of major mastitis pathogens in dairy cows

**Fig. 5:**
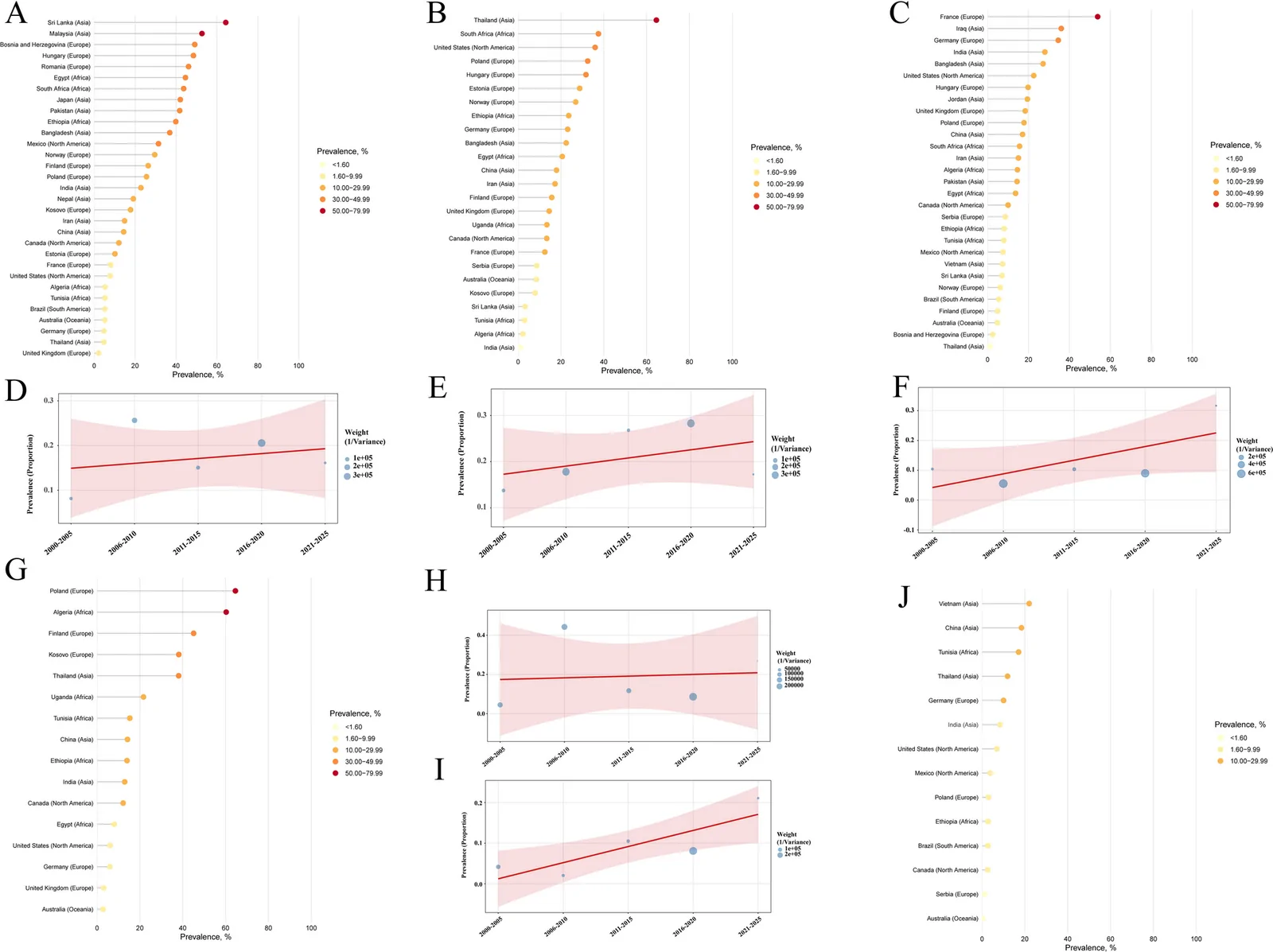
Spatiotemporal distribution of major pathogens causing dairy cow mastitis (2000–2025). **A** Country-level ranked prevalence plots for *Staphylococcus aureus*. **B** Country-level ranked prevalence plots for *Streptococcus* spp. **C** Country-level ranked prevalence plots for *Escherichia coli.* **D** Temporal trend in the prevalence of *Staphylococcus aureus* in causing dairy cow mastitis. **E** Temporal trend in the prevalence of *Streptococcus* spp. in causing dairy cow mastitis. **F** Temporal trend in the *incidence of E. coli* in causing dairy cow mastitis. **G** Country-level ranked prevalence plots for coagulase-negative staphylococci. **H** Temporal trend in the prevalence of coagulase-negative staphylococci in causing dairy cow mastitis. **I** Temporal trend in the prevalence of *Klebsiella* spp. in causing dairy cow mastitis. **J** Country-level ranked prevalence plots for *Klebsiella* spp.

Geographic analysis revealed significant differences among the five dominant pathogen categories (all *P* < 0.001) (Tables 1, 2, 3, 4, and 5). The corresponding numbers of continents and countries in these analyses were 6 and 31 for *S. aureus*, 5 and 25 for *Streptococcus* spp., 6 and 29 for *E. coli*, 5 and 16 for CNS, and 6 and 14 for *Klebsiella* spp., respectively (Fig. 5). Further analysis revealed clear pathogen-specific distribution patterns. *S. aureus* had the highest prevalence in Africa and Asia, with pooled prevalence values of 27.04% and 19.59%, respectively (Fig. 5A). *Streptococcus* spp. were most prevalent in Europe and Asia, with pooled prevalence values of 20.07% and 17.26%, respectively (Fig. 5B). *E. coli* was most prevalent in Asia and Europe, with pooled prevalence values of 17.49% and 14.26%, respectively (Fig. 5C). CNS was most prevalent in Europe, whereas *Klebsiella* spp. were most prevalent in Asia (Fig. 5G and J). Detailed continental estimates and confidence intervals are provided in Tables 1, 2, 3, 4, and 5. At the country level, hotspot regions also differed among the pathogen categories. *S. aureus* showed evidence of high prevalence in Sri Lanka and Malaysia, with country-level estimates exceeding 50%. *Streptococcus* spp. were most common in Thailand and South Africa, whereas *E. coli* had the highest country-level prevalence in France. CNS showed the highest prevalence values in Poland and Algeria, and *Klebsiella* spp. were most prominent in Vietnam.

Meta-regression analysis was used to evaluate temporal trends in the prevalence of the major bovine mastitis pathogens from 2000 to 2025 (Fig. 5D, E, F, H, and I). *S. aureus* showed a fluctuating but generally increasing pattern across sequential 5-year intervals. The prevalence of *Streptococcus* spp. increased from 2000 to 2020 but declined from 2021 to 2025. *E. coli* began to increase in prevalence after 2006, reaching 31.7% from 2021 to 2025, indicating an overall increasing trend. The prevalence of CNS peaked at 44.3% during 2006–2010 and then tended to stabilize. Among the five dominant pathogen categories, *Klebsiella* spp. showed the most pronounced upward temporal trajectory. In all the regression plots in the figures above, the bubble size represents the inverse-variance weight of each study.

Period-stratified geographic analysis revealed that the countries with the highest reported prevalence values changed over time, indicating that pathogen hotspots were dynamic rather than fixed over the 25-year period (Figs. S6–S10). For example, the leading hotspot regions for *S. aureus*, *E. coli*, CNS, and *Klebsiella* spp. shifted across successive periods, whereas *Streptococcus* spp. repeatedly demonstrated high prevalence values in North America during later periods. These results suggest that temporal changes in bovine mastitis pathogen patterns were accompanied by geographic redistributions of high-prevalence regions. Detailed period-specific hotspot countries and prevalence estimates are provided in Figs. S6–S10.

### Sources of heterogeneity in the prevalence of major pathogens causing mastitis in dairy cows

From 2000 to 2025, substantial heterogeneity was observed in the pooled prevalence estimates for all five dominant categories of pathogen causing bovine mastitis. To explore potential sources of heterogeneity, subgroup analyses were conducted according to continent, climate type, breed purity, and farming system (Tables 1, 2, 3, 4, and 5 and Figs. S11–S35). The results revealed that subgroup differences were pathogen specific rather than consistent for all predefined variables.

For the continent-level comparisons, significant between-subgroup differences were observed for *S. aureus*, *E. coli*, and *Klebsiella* spp. but not for *Streptococcus* spp. and CNS. Climate-related subgroup differences were significant for *S. aureus*, *Streptococcus* spp., and *E. coli* but not for CNS or *Klebsiella* spp. In contrast, breed purity did not significantly differ between the subgroups for any of the five dominant pathogen categories. Farming system showed a significant between subgroup difference only for *S. aureus*, while no significant differences were observed for *Streptococcus* spp., *E. coli*, CNS, or *Klebsiella* spp.

### Temporal trends and subgroup analyses of minor pathogens causing dairy cow mastitis

Geographic analysis revealed that minor mastitis pathogens exhibited uneven and pathogen-specific regional distribution patterns (Figs. S36–S45). The reported prevalence estimate of *Enterococcus* spp. was relatively high in Poland, China, Uganda, and Kosovo, whereas that of yeast species was particularly high in one Chinese study, with additional moderate estimates reported in Algeria and parts of Europe. High-prevalence estimates were also detected for *Pseudomonas* spp. and *Bacillus* spp., especially in individual studies from China, India, and Bangladesh. *Enterobacter* spp. were relatively more prevalent in Mexico than in other reported regions. Because several of these estimates were derived from a small number of studies, the validity of these apparent hotspots should be interpreted cautiously and verified further through standardized regional surveillance. Detailed country-level estimates are provided in Figs. S36–S45 and Tables S13–S22.

The geographic distribution of several minor pathogens also showed marked regional specificity (Figs. S36–S45). The prevalence of *Mycoplasma* spp. was higher in Iran (29.57%) and Estonia (17.24%) than in most other reported countries, whereas the prevalence of *Proteus* spp. was high in Mexico (28.30%). In contrast, *Corynebacterium* spp. showed a relatively even distribution without clear extreme hotspots, while the prevalence estimates of *Trueperella* spp. and *Lactococcus* spp. generally remained low.

Meta-regression analysis was used to evaluate the temporal trends in the prevalence estimates of minor mastitis pathogens from 2000 to 2025 (Figs. S36–S45). The prevalence of *Enterococcus* spp., *Pseudomonas* spp., yeast, and *Bacillus* spp. tended to increase overall, with more evident increases for *Pseudomonas* spp. and *Bacillus* spp. *Corynebacterium* spp. also showing a positive temporal trend in its prevalence, although the slope was relatively modest. In contrast, the abundances of *Mycoplasma* spp. and *Trueperella* spp. tended to decrease over time. In all the regression plots in the figures above, the bubble size represents the inverse-variance weight of each study.

Owing to insufficient continuous time series data, the prevalence estimates of *Enterobacter* spp., *Proteus* spp., and *Lactococcus* spp. could not be robustly analyzed using meta-regression. Therefore, temporal changes in the occurrence of these pathogens were evaluated descriptively using bar charts (Figs. S43B, S44B, and S45B). *Enterobacter* spp. were reported mainly between 2011 and 2020, whereas *Proteus* spp. and *Lactococcus* spp. were reported predominantly after 2016. Because the available data for these pathogens are sparse and temporally discontinuous, the current evidence is insufficient to support reliable long-term trend estimations.

## Discussion

This study provides a comprehensive overview of changes in the global mastitis pathogen spectrum. Overall, the findings of this meta-analysis suggest that dairy cow mastitis remains largely associated with several predominant pathogens, but the distribution of these pathogens is not static. Instead, the pathogen spectrum shows clear temporal shifts and marked spatial heterogeneity. Subgroup analyses suggested that geographic and climatic factors may contribute to this heterogeneity in a pathogen-specific manner. These results highlight the need for region-specific surveillance and targeted prevention strategies rather than a uniform, global control model.

In accordance with the PRISMA guidelines, in this study, global evidence on the etiology of dairy cow mastitis from 2000 to 2025 was systematically synthesized. Over the past 25 years, the trajectories for major mastitis pathogens, including *S. aureus*, *Streptococcus* spp., *E. coli*, CNS, and *Klebsiella* spp., reported in the literature have generally followed the overall growth of mastitis research. This observation suggests that these organisms have remained important within the global mastitis pathogen spectrum and continue to pose challenges for modern dairy production despite the use of long-standing control efforts. In contrast, less frequently reported pathogens appear to occupy a more peripheral but relatively stable position in the literature. Their limited representation may reflect opportunistic pathogenicity, regional clustering, lower detection priority, or inconsistent reporting across studies. Although current evidence does not indicate that these minor pathogens have become a major global research focus, they continue to be detected, suggesting that they should not be overlooked, particularly with regard to herd-specific outbreaks, antimicrobial resistance monitoring, and changes in host susceptibility.

Given the broad temporal and geographic scope of this meta-analysis, the substantial between-study heterogeneity was not unexpected. The use of random effects modeling, heterogeneity assessment, publication bias evaluation, and leave-one-out sensitivity analysis helped improve the reliability of the pooled estimates. Although evidence of possible publication bias or small-study effects was observed for several pathogens, the sensitivity analysis revealed that the overall estimates were not influenced disproportionately by any single study. To further evaluate whether the findings depended on a single statistical specification, the results of several random-effects models were evaluated, including the DerSimonian–Laird, restricted maximum likelihood, Paule–Mandel, and Hartung–Knapp-adjusted approaches. The broadly consistent estimates obtained with these models support the stability of the main conclusions under different assumptions for between-study variance estimation and uncertainty adjustment. This multimodel validation is particularly important, given the substantial heterogeneity observed in the global data, because reliance on a single random-effects estimator may lead to overconfidence in or model-dependent interpretations of the results. Nevertheless, such consistency across models does not eliminate the underlying across-study heterogeneity; rather, it indicates that the major conclusions are statistically robust, whereas the wide intervals still indicate that the prevalence of the different pathogens may vary considerably across regions, production systems, and future study settings. Therefore, the main findings regarding temporal changes and spatial variation in the major mastitis pathogen spectrum appear to be statistically stable.

The pooled prevalence estimates revealed that *S. aureus*, *Streptococcus* spp., CNS, *E. coli*, and *Klebsiella* spp. were the main pathogens associated with dairy cow mastitis globally. Among them, *S. aureus* and *Streptococcus* spp. were particularly prominent, which is consistent with their established roles as major contagious and environmental mastitis pathogens, respectively [27]. However, the extremely high heterogeneity observed across the studies indicates that these pooled estimates should be interpreted as global reference values rather than fixed benchmarks for specific countries, regions, or farms. The wide confidence intervals further suggest that local pathogen prevalence estimates may deviate substantially from the global mean. Therefore, the global pooled estimates are the most useful for identifying broad pathogen patterns, whereas regionally, control targets should be calibrated on the basis of local surveillance data, diagnostic practices, herd structure, and management conditions.

Geographic analysis revealed marked spatial heterogeneity in the distribution of the major mastitis pathogens. These differences may reflect true epidemiological variability involving factors such as climate, herd structure, biosecurity conditions, and production systems, but they may also be shaped by differences in case composition, sampling strategy, diagnostic method, study population, and sample size [28]. Similar caution is needed when interpreting the results for minor pathogens, for which isolated, high estimates in certain regions may be more sensitive to sparse-data or local reporting effects. Therefore, future surveillance efforts should prioritize standardized diagnostic protocols and more balanced geographic coverage to verify apparent hotspots and improve the comparability of pathogen-prevalence estimates across regions.

The results of the temporal analysis suggested that the major mastitis pathogen spectrum has not remained fixed but rather has changed over the past 25 years. *S. aureus*, *E. coli*, and *Klebsiella* spp. in particular showed evidence of persistent or increasing abundance, whereas the patterns for *Streptococcus* spp. and CNS appeared to be more variable over time. These changes may be related to shifts in herd management, housing systems, antimicrobial use, diagnostic capacity, and environmental exposure. However, the period-specific estimates should also be interpreted cautiously because differences in sample size, study weight, and regional data availability may influence the apparent direction of the temporal trends. The increasing prevalence estimates observed for *Klebsiella* spp. are especially noteworthy because this pathogen is often associated with environmental reservoirs and severe clinical outcomes, suggesting that it should receive greater attention in future mastitis surveillance and control programs.

The results of geographical-temporal analysis further suggest that regional, high-risk profiles for mastitis pathogens are dynamic rather than fixed. Apparent shifts in pathogen hotspots over time may reflect changes in dairy industry structure, herd management, diagnostic strategies, antimicrobial use, biosecurity practices, and environmental pressures [29], but they may also be affected by uneven surveillance efforts and differences in the studies available for each period and region. Therefore, mastitis prevention and control strategies should be developed on the basis of regularly updated pathogen profiles rather than isolated cross-sectional evidence or empirical assumptions. Establishing continuous regional surveillance systems could help identify emerging hotspots, allow adjustment of intervention priorities, and improve the timeliness of pathogen-specific control strategies.

The temporal changes described above may be partly understood in the context of the structural transformation of the dairy industry. Over the past 25 years, dairy production has become increasingly more intense, with wider adoption of automated milking systems and growing concerns about antimicrobial resistance [30, 31]. Changes in housing conditions, environmental exposure, stocking density, milking procedures, and hygiene management may have altered the relative abundances of environmental pathogens such as *E. coli* and *Klebsiella* spp. in some regions. In addition, the changing profiles of less frequently reported pathogens suggests that opportunistic or atypical organisms may become regionally abundant under specific management, environmental, or therapeutic conditions. Such shifts may not only reflect true epidemiological changes but also be associated with improved diagnostic coverage and greater reporting capacity. Therefore, future surveillance systems should not only focus on traditional major pathogens but also include specific minor or emerging pathogens, especially in regions where management practices, antimicrobial use, or environmental conditions are changing rapidly.

The observed differences across climate zones suggest that climatic conditions may be important contributors to the global heterogeneity in the distributions of mastitis pathogens. Temperature, humidity, bedding moisture, heat stress, and the barn microenvironment can influence pathogen survival, environmental contamination, teat-end exposure, and infection pressure [32]. However, the effect of climate is not deterministic, as it may be modified by farm management. In warm and humid regions, for example, improved bedding management, ventilation, environmental hygiene, and heat-stress control may help reduce the burden of environmental pathogens.

Production systems may shape pathogen distribution. Differences between intensive and extensive systems are particularly important for environmental or opportunistic pathogens, as stocking density, housing condition, manure accumulation, bedding management, and milking hygiene can directly affect exposure pressure. In contrast, contagious or persistent pathogens may be influenced not only by the production system itself but also by herd-carrier status, milking-time transmission, chronic infection management, and culling strategies. The findings of this study indicate that climate- and production-related risks should be considered jointly when region-specific mastitis control programs are designed.

Breed structure may also contribute to the observed heterogeneity in mastitis pathogen distribution. Differences between purebred and cross-bred dairy cows should be interpreted cautiously, as breed composition is often closely linked with geography, production intensity, herd size, housing conditions, and management practices [33–35]. High-yielding purebred dairy cows may face greater metabolic and udder health pressure than their cross-bred counterparts, whereas cross-bred dairy cows may be raised under distinct environmental and management conditions. Therefore, the apparent breed-related differences in pathogen prevalence were likely shaped by the combined effects of genetic background, immune response, anatomical traits, and production systems [36–38]. Practically, mastitis prevention efforts should be stratified not only by region, climate, and time period but also by herd structure and farm management. The findings of this study also have implications for mastitis resistance breeding: rather than rely on broad, nonspecific selection for mastitis resistance, future breeding programs should integrate pathogen-specific phenotypes with candidate immune-related genes or SNP markers, such as *TLR4* and *BoLA*, to improve selection precision [39, 40]. In addition, regional differences in pathogen spectra suggest that the breeding objectives may need to be adapted to the local epidemiological condition. Such locally informed cross-breeding may help balance milk production, environmental adaptability, and disease resistance, but such strategies would need to be validated using farm-level pathogen surveillance and genetic performance data.

In some countries and regions, limited access to routine bacteriological testing results in a reliance on clinical signs for mastitis treatment decision-making, which may reduce the precision of antimicrobial use [41–43]. The spatiotemporal heterogeneity observed in this study further supports the need for auxiliary strategies tailored to local pathogen profiles. Targeted nutritional regulation strategies may complement hygiene management, diagnostic testing, and antimicrobial stewardship by improving host immunity, maintaining mammary epithelial barrier function, and reducing inflammatory damage.

Regarding Gram-positive pathogens such as *S. aureus*, *Streptococcus* spp., and CNS, previous studies have suggested that selenium-vitamin E, ω-3 polyunsaturated fatty acids, and probiotics may support antioxidant levels, immune regulation, and epithelial barrier integrity [44–47]. With respect to Gram-negative pathogens such as *E. coli* and *Klebsiella* spp., plant essential oils, tannins, and prebiotics have been investigated for their potential roles in altering microbial balance and alleviating endotoxin-related mammary inflammation [48–51]. The development of nutritional strategies may also need to consider climate and production systems, such as heat-stress mitigation in hot and humid regions or mineral and immune-supportive supplementation depending on the management condition. However, these interventions should be regarded as supportive measures rather than substitutes for pathogen identification, environmental control, and rational antimicrobial use.

Future mastitis control strategies should combine spatial surveillance with temporal trend monitoring to support regular updates of regional pathogen profiles. Such systems could help identify changes in dominant pathogens early and guide timely adjustments of prevention priorities. In warm and humid regions or for intensive production systems, environmental management, including bedding control, ventilation, hygiene, and heat-stress mitigation, should be strengthened to reduce environmental pathogen pressure. In herds with a high contagious or persistent pathogen burden, milking procedures, teat disinfection, identification of carrier cows, and chronic infection management should be prioritized. Overall, pathogen-specific control programs should be adaptive, informed by local conditions, and regularly updated rather than be based on static global averages.

This study has several limitations. First, the included studies were unevenly distributed across regions, with relatively greater representation from research-heavy areas and limited evidence from some continents. This unevenness may affect the representativeness of the global pooled estimates. Second, climate and continental information was not consistently reported in the original studies. Consequently, some subgroup variables had to be inferred from the sampling location, and climate classification was limited when the country covered multiple climate zones, which may have reduced the specificity of the climate-related analyses. Third, differences in sampling strategies, diagnostic methods, study populations, and clinical or subclinical case composition likely contributed to the substantial heterogeneity observed across the studies. These limitations should be considered when interpreting the global estimates and applying the findings to specific regional or farm-level contexts. Overall, however, these results should be interpreted with caution. Differences in study scale, sampling strategy, diagnostic method, reporting practice, and regional surveillance effort may partly explain the observed heterogeneity, and thus the results should not be interpreted entirely as true epidemiological changes [52, 53]. In particular, estimates for less frequently reported pathogens may be more vulnerable to sparse-data and regional reporting bias. Thus, although this study provides a useful reference for understanding mastitis pathogen distributions worldwide, regional prevention and control strategies should still be developed on the basis of local surveillance data and farm-level epidemiological evidence [54].

Future studies should promote standardized case definitions, sampling strategies, and diagnostic procedures to improve comparability across regions and reduce methodological heterogeneity. Greater continuity in surveillance is also needed in underrepresented regions to improve the balance and reliability of the global evidence. Additionally, regional studies should consider investigating pathogens with potentially emerging importance and evaluate their relationships with environmental management, antimicrobial use, and animal resistance profiles. Such efforts would help distinguish true epidemiological changes from differences caused by surveillance effort, diagnostic capacity, or reporting practices.

Future studies could use the spatiotemporal pathogen distribution data generated in this study to conduct genome-wide association analyses of pathogen-specific resistance traits, identify key loci associated with resistance to dominant pathogens, and support the development of molecular breeding tools for precision selection. In parallel, future research should further investigate the associations among pathogen-specific mammary damage mechanisms, nutritional regulation, and mammary gland health, with the goal of developing regional and pathogen-specific nutritional strategies for dairy cows and improving combined mastitis prevention and control systems.

## Conclusions

The results revealed substantial temporal and spatial worldwide variability in bovine mastitis pathogens. Subgroup analyses similarly suggested pathogen-specific geographic and climatic variation, whereas differences according to breed purity and farming system were less consistent and should be interpreted with caution. The findings of this meta-analysis provide a long-term, global, empirical basis for veterinary epidemiological prevention and control, including the establishment of regional surveillance systems, the updating of control programs, and the rational use of antimicrobial agents. Furthermore, these findings provide key data-based support for genetic breeding for bovine mastitis resistance, including the need for molecular marker screening and regionally tailored breeding, as well as targeted nutritional regulation of mammary gland health. Overall, these findings may promote the interdisciplinary integration of animal genetic improvement, nutritional science, and epidemic prevention and control while offering new insights for global, healthy dairy farming and improved production performance. Despite evidence of potential publication bias or small-study effects for some pathogen categories, leave-one-out sensitivity analyses supported the robustness of the pooled global estimates.

Future studies should further strengthen standardized regional surveillance and translate pathogen-specific distribution data into locally adapted breeding, nutritional, and management strategies.

## Supplementary Information

Additional file 1: Table S1. PRISMA2020 checklist. Table S2. Literature search syntax for the pathogen spectrum of dairy cow mastitis. Table S3. Included literature and source information. Table S4. Inclusion and exclusion criteria for studies on the pathogen spectrum of dairy cow mastitis. Table S5. Risk of bias assessment based on STROBE criteria. Table S6. Bias assessment results for included studies based on STROBE criteria. Table S7. Quality assessment checklist. Table S8. Quality score and quality index score of individual contributing studies. Table S9. Data extraction variables from reviewed literature. Table S10. Bias in the inclusion of studies for multi-model validation. Table S11. Sensitivity analysis using alternative random-effects models for the five dominant pathogen categories. Table S12. Sensitivity analysis restricted to high-quality studies. Table S13. Research information and epidemiological situation of *Enterococcus* spp. causing bovine mastitis. Table S14. Research information and epidemiological situation of *Pseudomonas *spp. causing bovine mastitis. Table S15. Research information and epidemiological situation of *Bacillus* spp. causing bovine mastitis. Table S16. Research information and epidemiological situation of yeast causing bovine mastitis. Table S17. Research information and epidemiological situation of *Corynebacterium* spp. causing bovine mastitis. Table S18. Research information and epidemiological situation of *Enterobacter* spp. causing bovine mastitis. Table S19. Research information and epidemiological situation of *Mycoplasma* spp. causing bovine mastitis. Table S20. Research information and epidemiological situation of *Trueperella* spp. causing bovine mastitis. Table S21. Research information and epidemiological situation of *Proteus* spp. causing bovine mastitis. Table S22. Research information and epidemiological situation of* Lactococcus* spp. causing bovine mastitis. Fig. S1. Funnel plot for the meta-analysis of bovine mastitis caused by *S. aureus*. Fig. S2. Funnel plot for the meta-analysis of bovine mastitis caused by *Streptococcus* spp. Fig. S3. Funnel plot for the meta-analysis of bovine mastitis caused by *E. coli*. Fig. S4. Funnel plot for the meta-analysis of bovine mastitis caused by CNS. Fig. S5. Funnel plot for the meta-analysis of bovine mastitis caused by* Klebsiella* spp. Fig. S6. Period-specific hotspot countries for* S. aureus* infection in dairy cow mastitis. Fig. S7. Period-specific hotspot countries for*Streptococcus* spp. infection in dairy cow mastitis. Fig. S8. Period-specific hotspot countries for *E. coli* infection in dairy cow mastitis. Fig. S9. Period-specific hotspot countries for CNS infection in dairy cow mastitis. Fig. S10. Period-specific hotspot countries for *Klebsiella* spp. infection in dairy cow mastitis. Fig. S11. Prevalence of *S. aureus* in dairy cow mastitis: a meta-analysis and subgroup analysis by country. Fig. S12. Prevalence of *S. aureus* in dairy cow mastitis: a meta-analysis and subgroup analysis by continent. Fig. S13. Prevalence of *S. aureus* in dairy cow mastitis: a meta-analysis and subgroup analysis by climate. Fig. S14. Prevalence of *S. aureus *in dairy cow mastitis: a meta-analysis and subgroup analysis by breed purity. Fig. S15. Prevalence of *S. aureus* in dairy cow mastitis: a meta-analysis and subgroup analysis by intensification. Fig. S16. Prevalence of *Streptococcus* spp. in dairy cow mastitis: a meta-analysis and subgroup analysis by country. Fig. S17. Prevalence of *Streptococcus* spp. in dairy cow mastitis: a meta-analysis and subgroup analysis by continent. Fig. S18. Prevalence of *Streptococcus* spp. in dairy cow mastitis: a meta-analysis and subgroup analysis by climate. Fig. S19. Prevalence of *Streptococcus* spp. in dairy cow mastitis: a meta-analysis and subgroup analysis by breed purity. Fig. S20. Prevalence of *Streptococcus* spp. in dairy cow mastitis: a meta-analysis and subgroup analysis by intensification. Fig. S21. Prevalence of *E. coli *in dairy cow mastitis: a meta-analysis and subgroup analysis by country. Fig. S22. Prevalence of *E. coli* in dairy cow mastitis: a meta-analysis and subgroup analysis by continent. Fig. S23. Prevalence of *E. coli* in dairy cow mastitis: a meta-analysis and subgroup analysis by climate. Fig. S24. Prevalence of* E. coli* in dairy cow mastitis: a meta-analysis and subgroup analysis by breed purity. Fig. S25. Prevalence of *E. coli* in dairy cow mastitis: a meta-analysis and subgroup analysis by intensification. Fig. S26. Prevalence of CNS in dairy cow mastitis: a meta-analysis and subgroup analysis by country. Fig. S27. Prevalence of CNS in dairy cow mastitis: a meta-analysis and subgroup analysis by continent. Fig. S28. Prevalence of CNS in dairy cow mastitis: a meta-analysis and subgroup analysis by climate. Fig. S29. Prevalence of CNS in dairy cow mastitis: a meta-analysis and subgroup analysis by breed purity. Fig. S30. Prevalence of CNS in dairy cow mastitis: a meta-analysis and subgroup analysis by intensification. Fig. S31. Prevalence of *Klebsiella* spp. in dairy cow mastitis: a meta-analysis and subgroup analysis by country. Fig. S32. Prevalence of *Klebsiella* spp. in dairy cow mastitis: a meta-analysis and subgroup analysis by continent. Fig. S33. Prevalence of *Klebsiella* spp. in dairy cow mastitis: a meta-analysis and subgroup analysis by climate. Fig. S34. Prevalence of *Klebsiella* spp. in dairy cow mastitis: a meta-analysis and subgroup analysis by breed purity. Fig. S35. Prevalence of *Klebsiella* spp. in dairy cow mastitis: a meta-analysis and subgroup analysis by intensification. Fig. S36. Spatiotemporal distribution of *Enterococcus* spp. causing dairy cow mastitis (2000–2025). Fig. S37. Spatiotemporal distribution of *Pseudomonas* spp. causing dairy cow mastitis (2000–2025). Fig. S38. Spatiotemporal distribution of yeast causing dairy cow mastitis (2000–2025). Fig. S39. Spatiotemporal distribution of *Bacillus* spp. causing dairy cow mastitis (2000–2025). Fig. S40. Spatiotemporal distribution of *Corynebacterium* spp. causing dairy cow mastitis (2000–2025). Fig. S41. Spatiotemporal distribution of *Mycoplasma* spp. causing dairy cow mastitis (2000–2025). Fig. S42. Spatiotemporal distribution of *Trueperella* spp. causing dairy cow mastitis (2000–2025). Fig. S43. Spatiotemporal distribution of *Enterobacter* spp. causing dairy cow mastitis (2000–2025). Fig. S44. Spatiotemporal distribution of *Proteus* spp. causing dairy cow mastitis (2000–2025). Fig. S45. Spatiotemporal distribution of *Lactococcus* spp. causing dairy cow mastitis (2000–2025).

## Data Availability

The supplemental material, analytic code, original data, and findings of this study are available at https://doi.org/10.6084/m9.figshare.31120591.

## Abbreviations

BoLA: Bovine leukocyte antigen
CI: Confidence interval
CNS: Coagulase-negative staphylococci
PRISMA: Preferred Reporting Items for Systematic Reviews and Meta-Analyses
SNP: Single nucleotide polymorphism
STROBE: Strengthening the Reporting of Observational Studies in Epidemiology
TLR4: Toll-like receptor 4

## References

[1] Hogeveen H, Huijps K, Lam TJ. Economic aspects of mastitis: new developments. N Z Vet J. 2011;59(1):16–23. 21328153 10.1080/00480169.2011.547165

[2] Ruegg PL. A 100-year review: mastitis detection, management, and prevention. J Dairy Sci. 2017;100(12):10381–97. 29153171 10.3168/jds.2017-13023

[3] Liang D, Arnold LM, Stowe CJ, Harmon RJ, Bewley JM. Estimating US dairy clinical disease costs with a stochastic simulation model. J Dairy Sci. 2017;100(2):1472–86. 28012631 10.3168/jds.2016-11565

[4] Coherent Market Insights. Bovine mastitis market, by type, by product, by route of administration, by therapy, by end user, and by region: size, share, outlook, and opportunity analysis, 2025–2032. Burlingame, CA: Coherent Market Insights; 2025. Report No. 1699457.

[5] Ashraf A, Imran M. Causes, types, etiological agents, prevalence, diagnosis, treatment, prevention, effects on human health and future aspects of bovine mastitis. Anim Health Res Rev. 2020;21(1):36–49. 32051050 10.1017/S1466252319000094

[6] Sharun K, Dhama K, Tiwari R, Gugjoo MB, Iqbal Yatoo M, Patel SK, et al. Advances in therapeutic and managemental approaches of bovine mastitis: a comprehensive review. Vet Q. 2021;41(1):107–36. 33509059 10.1080/01652176.2021.1882713 PMC7906113

[7] Qiu Y, Fu S, Sun N, Yang B, Feng S, Zhang J, et al. Seasonal and regional dynamics of mastitis pathogens: insights from a 3-year study in China. Transbound Emerg Dis. 2025;2025:3631905. 41346562 10.1155/tbed/3631905 PMC12674873

[8] Tora ET, Bekele NB, Suresh Kumar RS. Bacterial profile of bovine mastitis in Ethiopia: a systematic review and meta-analysis. PeerJ. 2022;10:e13253. 35547189 10.7717/peerj.13253 PMC9083533

[9] Sorensen LP, Mark T, Madsen P, Lund MS. Genetic correlations between pathogen-specific mastitis and somatic cell count in Danish Holsteins. J Dairy Sci. 2009;92(7):3457–71. 19528624 10.3168/jds.2008-1870

[10] Schukken YH, Lam TJGM, Barkema HW. Biological basis for selection on udder health. Interbull Bull. 1997;15:27–33.

[11] Gonzalez-Chavez SA, Arevalo-Gallegos S, Rascon-Cruz Q. Lactoferrin: structure, function and applications. Int J Antimicrob Agents. 2009;33(4):301 e1-308. 18842395 10.1016/j.ijantimicag.2008.07.020

[12] Pyorala S. Indicators of inflammation in the diagnosis of mastitis. Vet Res. 2003;34(5):565–78. 14556695 10.1051/vetres:2003026

[13] Seegers H, Fourichon C, Beaudeau F. Production effects related to mastitis and mastitis economics in dairy cattle herds. Vet Res. 2003;34(5):475–91. 14556691 10.1051/vetres:2003027

[14] Thompson-Crispi KA, Sewalem A, Miglior F, Mallard BA. Genetic parameters of adaptive immune response traits in Canadian Holsteins. J Dairy Sci. 2012;95(1):401–9. 22192219 10.3168/jds.2011-4452

[15] Gaworski M. Implementation of technical and technological progress in dairy production. Processes. 2021;9(12):2103.

[16] Page MJ, McKenzie JE, Bossuyt PM, Boutron I, Hoffmann TC, Mulrow CD, et al. The PRISMA 2020 statement: an updated guideline for reporting systematic reviews. BMJ. 2021;372:n71. 10.1136/bmj.n71 PMC8005924 33782057

[17] Cuschieri S. The STROBE guidelines. Saudi J Anaesth. 2019;13(Suppl 1):S31–4. 30930717 10.4103/sja.SJA_543_18 PMC6398292

[18] Su Y, Zhao G, Xu J, Wang Y. Mapping the global distribution of *Mycoplasma bovis* infections in cattle (2000-2024): a geospatial modeling analysis. J Dairy Sci. 2000;108(9):10099–124. 10.3168/jds.2025-26513 40639651

[19] Hoy D, Brooks P, Woolf A, Blyth F, March L, Bain C, et al. Assessing risk of bias in prevalence studies: modification of an existing tool and evidence of interrater agreement. J Clin Epidemiol. 2012;65(9):934–9. 22742910 10.1016/j.jclinepi.2011.11.014

[20] DerSimonian R, Laird N. Meta-analysis in clinical trials revisited. Contemp Clin Trials. 2015;45(Pt A):139–45. 26343745 10.1016/j.cct.2015.09.002 PMC4639420

[21] Hartung J, Knapp G. On tests of the overall treatment effect in meta-analysis with normally distributed responses. Stat Med. 2001;20(12):1771–82. 11406840 10.1002/sim.791

[22] Paule RC, Mandel J. Consensus values, regressions, and weighting factors. J Res Natl Inst Stand Technol. 1989;94(3):197–203. 28053410 10.6028/jres.094.020 PMC4943748

[23] Sidik K, Jonkman JN. A simple confidence interval for meta-analysis. Stat Med. 2002;21(21):3153–9. 12375296 10.1002/sim.1262

[24] Veroniki AA, Jackson D, Viechtbauer W, Bender R, Bowden J, Knapp G, et al. Methods to estimate the between-study variance and its uncertainty in meta-analysis. Res Synth Methods. 2016;7(1):55–79. 26332144 10.1002/jrsm.1164 PMC4950030

[25] Viechtbauer W, Cheung MW. Outlier and influence diagnostics for meta-analysis. Res Synth Methods. 2010;1(2):112–25. 26061377 10.1002/jrsm.11

[26] Deeks JJ, Higgins JPT, Altman DG, McKenzie JE, Veroniki AA. Analysing data and undertaking meta-analyses. In: Higgins JPT, Thomas J, Chandler J, Cumpston M, Li T, Page MJ, Welch VA, editors. Cochrane handbook for systematic reviews of interventions version 6.5. Cochrane; 2024.

[27] Zadoks R, Fitzpatrick J. Changing trends in mastitis. Ir Vet J. 2009;62(Suppl 4):S59. 10.1186/2046-0481-62-S4-S59 PMC3339353 22082032

[28] Reyher KK, Haine D, Dohoo IR, Revie CW. Examining the effect of intramammary infections with minor mastitis pathogens on the acquisition of new intramammary infections with major mastitis pathogens--a systematic review and meta-analysis. J Dairy Sci. 2012;95(11):6483–502. 22981582 10.3168/jds.2012-5594

[29] Thompson-Crispi K, Atalla H, Miglior F, Mallard BA. Bovine mastitis: frontiers in immunogenetics. Front Immunol. 2014;5:493. 25339959 10.3389/fimmu.2014.00493 PMC4188034

[30] Coombe JE, Tymms SM, Humphris M. Antimicrobial stewardship in the dairy industry: responding to the threat of antimicrobial resistance. Aust Vet J. 2019;97(7):231–2. 31236929 10.1111/avj.12807

[31] Jacobs JA, Siegford JM. Invited review: the impact of automatic milking systems on dairy cow management, behavior, health, and welfare. J Dairy Sci. 2012;95(5):2227–47. 22541453 10.3168/jds.2011-4943

[32] Hogan JS, Smith KL, Hoblet KH, Todhunter DA, Schoenberger PS, Hueston WD, et al. Bacterial counts in bedding materials used on nine commercial dairies. J Dairy Sci. 1989;72(1):250–8. 2925950 10.3168/jds.s0022-0302(89)79103-7

[33] Coombe JE, Morton JM, Beggs DS, Dodds MJ, Pyman MF. Breed structures in Australian dairy herds. Aust Vet J. 2022;100(1–2):29–39. 34651306 10.1111/avj.13126

[34] Curry BA, Dukkipati VSR, López-Villalobos N. Comparative study of first lactation performance of Norwegian Red crossbred cows with traditional breeds in New Zealand dairy systems. N Z J Agric Res. 2025;68(4):712–7.

[35] Mota RR, Brito LF, Berry DP. Editorial: beef on dairy: the use of a simple tool to improve both cattle production systems. Front Genet. 2022;13:813949. 35559015 10.3389/fgene.2022.813949 PMC9086432

[36] Begley N, Buckley F, Pierce KM, Fahey AG, Mallard BA. Differences in udder health and immune response traits of Holstein-Friesians, Norwegian reds, and their crosses in second lactation. J Dairy Sci. 2009;92(2):749–57. 19164687 10.3168/jds.2008-1356

[37] Cai Z, Iso-Touru T, Sanchez MP, Kadri N, Bouwman AC, Chitneedi PK, et al. Meta-analysis of six dairy cattle breeds reveals biologically relevant candidate genes for mastitis resistance. Genet Sel Evol. 2024;56:54. 10.1186/s12711-024-00920-8 PMC11247842 39009986

[38] Neijenhuis F, Barkema HW, Hogeveen H, Noordhuizen JP. Relationship between teat-end callosity and occurrence of clinical mastitis. J Dairy Sci. 2001;84(12):2664–72. 11814022 10.3168/jds.S0022-0302(01)74720-0

[39] Rupp R, Hernandez A, Mallard BA. Association of bovine leukocyte antigen (BoLA) DRB3.2 with immune response, mastitis, and production and type traits in Canadian Holsteins. J Dairy Sci. 2007;90(2):1029–38. 17235182 10.3168/jds.S0022-0302(07)71589-8

[40] Sharma BS, Leyva I, Schenkel F, Karrow NA. Association of toll-like receptor 4 polymorphisms with somatic cell score and lactation persistency in Holstein bulls. J Dairy Sci. 2006;89(9):3626–35. 16899698 10.3168/jds.S0022-0302(06)72402-X

[41] Gonzalez Pereyra V, Pol M, Pastorino F, Herrero A. Quantification of antimicrobial usage in dairy cows and preweaned calves in Argentina. Prev Vet Med. 2015;122(3):273–9. 26558514 10.1016/j.prevetmed.2015.10.019

[42] Muloi DM, Ibayi EL, Nyotera S, Kirimi H, Abdi AM, Mutinda SM, et al. Treatment strategies and antibiotic usage practices in mastitis management in Kenyan smallholder dairy farms. BMC Vet Res. 2025;21:212. 10.1186/s12917-025-04662-7 PMC11951627 40148949

[43] Tomazi T, Dos Santos MV. Antimicrobial use for treatment of clinical mastitis in dairy herds from Brazil and its association with herd-level descriptors. Prev Vet Med. 2020;176:104937. 32126401 10.1016/j.prevetmed.2020.104937

[44] Bouchard DS, Rault L, Berkova N, Le Loir Y, Even S. Inhibition of *Staphylococcus aureus* invasion into bovine mammary epithelial cells by contact with live *Lactobacillus casei*. Appl Environ Microbiol. 2013;79(3):877–85. 23183972 10.1128/AEM.03323-12 PMC3568545

[45] Desbois AP, Smith VJ. Antibacterial free fatty acids: activities, mechanisms of action and biotechnological potential. Appl Microbiol Biotechnol. 2010;85(6):1629–42. 19956944 10.1007/s00253-009-2355-3

[46] Hoque MN, Das ZC, Rahman A, Hoque MM. Effect of administration of vitamin E, selenium and antimicrobial therapy on incidence of mastitis, productive and reproductive performances in dairy cows. Int J Vet Sci Med. 2016;4(2):63–70. 30255040 10.1016/j.ijvsm.2016.11.001 PMC6145041

[47] Yang Y, Lv S, Wang Z, Liu J. Selenium ameliorates *S. aureus*-induced inflammation in bovine mammary epithelial cells by regulating ROS-induced NLRP3 inflammasome. Biol Trace Elem Res. 2022;200(7):3171–5. 34535880 10.1007/s12011-021-02924-7

[48] Morales-Ubaldo AL, Rivero-Perez N, Valladares-Carranza B, Velazquez-Ordonez V, Delgadillo-Ruiz L, Zaragoza-Bastida A. Bovine mastitis, a worldwide impact disease: prevalence, antimicrobial resistance, and viable alternative approaches. Vet Anim Sci. 2023;21:100306. 37547227 10.1016/j.vas.2023.100306 PMC10400929

[49] Rani S, Verma S, Singh H, Ram C. Antibacterial activity and mechanism of essential oils in combination with medium-chain fatty acids against predominant bovine mastitis pathogens. Lett Appl Microbiol. 2022;74(6):959–69. 35178733 10.1111/lam.13675

[50] Smith AH, Imlay JA, Mackie RI. Increasing the oxidative stress response allows *Escherichia coli* to overcome inhibitory effects of condensed tannins. Appl Environ Microbiol. 2003;69(6):3406–11. 12788743 10.1128/AEM.69.6.3406-3411.2003 PMC161476

[51] Wang Y, Nan X, Zhao Y, Jiang L, Wang H, Zhang F, et al. Dietary supplementation of inulin ameliorates subclinical mastitis via regulation of rumen microbial community and metabolites in dairy cows. Microbiol Spectr. 2021;9(2):e0010521. 34494854 10.1128/Spectrum.00105-21 PMC8557905

[52] Egger M, Davey Smith G, Schneider M, Minder C. Bias in meta-analysis detected by a simple, graphical test. BMJ. 1997;315(7109):629–34. 9310563 10.1136/bmj.315.7109.629 PMC2127453

[53] Mavridis D, Chaimani A, Efthimiou O, Leucht S, Salanti G. Addressing missing outcome data in meta-analysis. Evid Based Ment Health. 2014;17(3):85–9. 25009175 10.1136/eb-2014-101900 PMC13404265

[54] Olde Riekerink RG, Barkema HW, Kelton DF, Scholl DT. Incidence rate of clinical mastitis on Canadian dairy farms. J Dairy Sci. 2008;91(4):1366–77. 18349229 10.3168/jds.2007-0757

