# Supplementary material for: Global spatiotemporal patterns of bovine mastitis pathogens from 2000 to 2025: a meta-analysis for precision dairy health
Source: J Anim Sci Biotechnol. 2026 Sep 10;17:178. doi: 10.1186/s40104-026-01488-7 (PMC13560248; doi:10.1186/s40104-026-01488-7)
Supplement: Supplementary file 1 [file 40104_2026_1488_MOESM1_ESM.docx]

**Supplemental materials for**

**Global spatiotemporal patterns of bovine mastitis pathogens from 2000 to 2025: A meta-analysis for precision dairy health**

**Supplementary tables and legends**

**Tables S1-22**

**Figs. S1-45**

**Contents**

[**Table S1. PRISMA2020 checklist.**	10](#_Toc230889983)

[**Table S2. Literature search syntax for the pathogen spectrum of dairy cow mastitis.**	16](#_Toc230889984)

[**Table S3. Included literature and source information.**	17](#_Toc230889985)

[**Table S4. Inclusion and exclusion criteria for studies on the pathogen spectrum of dairy cow mastitis.**	21](#_Toc230889986)

[**Table S5. Risk of bias assessment based on STROBE criteria.**	22](#_Toc230889987)

[**Table S6. Bias assessment results for included studies based on STROBE criteria.**	23](#_Toc230889988)

[**Table S7. Quality assessment checklist.**	26](#_Toc230889989)

[**Table S8. Quality score and quality index score of individual contributing studies.**	27](#_Toc230889990)

[**Table S9. Data extraction variables from reviewed literature.**	31](#_Toc230889991)

[**Table S10.** **Bias in the inclusion of studies for multi-model validation.**	32](#_Toc230889992)

[**Table S11. Sensitivity analysis using alternative random-effects models for the five dominant pathogen categories.**	33](#_Toc230889993)

[**Table S12. Sensitivity analysis restricted to high-quality studies.**	34](#_Toc230889994)

[**Table S13.** **Research information and epidemiological situation of *Enterococcus* spp. causing bovine mastitis.**	35](#_Toc230889995)

[**Table S14.** **Research information and epidemiological situation of *Pseudomonas* spp. causing bovine mastitis.**	36](#_Toc230889996)

[**Table S15.** **Research information and epidemiological situation of *Bacillus* spp. causing bovine mastitis.**	37](#_Toc230889997)

[**Table S16.** **Research information and epidemiological situation of *Yeast* causing bovine mastitis.**	38](#_Toc230889998)

[**Table S17.** **Research information and epidemiological situation of *Corynebacterium* spp. causing bovine mastitis.**	39](#_Toc230889999)

[**Table S18.** **Research information and epidemiological situation of *Enterobacter* spp. causing bovine mastitis.**	40](#_Toc230890000)

[**Table S19.** **Research information and epidemiological situation of *Mycoplasma* spp. causing bovine mastitis.**	41](#_Toc230890001)

[**Table S20.** **Research information and epidemiological situation of** ***Trueperella* spp. causing bovine mastitis.**	42](#_Toc230890002)

[**Table S21.** **Research information and epidemiological situation of** ***Proteus* spp. causing bovine mastitis.**	43](#_Toc230890003)

[**Table S22.** **Research information and epidemiological situation of** ***Lactococcus* spp. causing bovine mastitis.**	44](#_Toc230890004)

[**Fig. S1.** **Funnel plot for the meta-analysis of bovine mastitis caused by *S. aureus*.**	45](#_Toc230890005)

[**Fig. S2.** **Funnel plot for the meta-analysis of bovine mastitis caused by *Streptococcus* spp.**	46](#_Toc230890006)

[**Fig. S3.** **Funnel plot for the meta-analysis of bovine mastitis caused by** ***E.coli*.**	47](#_Toc230890007)

[**Fig. S4.** **Funnel plot for the meta-analysis of bovine mastitis caused by** **CNS*.***	48](#_Toc230890008)

[**Fig. S5.** **Funnel plot for the meta-analysis of bovine mastitis caused by** ***Klebsiella* spp*.***	49](#_Toc230890009)

[**Fig. S6. Period-specific hotspot countries for *S. aureus* infection in dairy cow mastitis.**	50](#_Toc230890010)

[**Fig. S7. Period-specific hotspot countries for *Streptococcus* spp. infection in dairy cow mastitis.**	51](#_Toc230890012)

[**Fig. S8. Period-specific hotspot countries for *E. coli* infection in dairy cow mastitis.**	52](#_Toc230890014)

[**Fig. S9. Period-specific hotspot countries for CNS infection in dairy cow mastitis.**	53](#_Toc230890015)

[**Fig. S10. Period-specific hotspot countries for *Klebsiella* spp. infection in dairy cow mastitis.**	54](#_Toc230890017)

[**Fig. S11. Prevalence of *S. aureus* in dairy cow mastitis: a meta-analysis and subgroup analysis by country.**	55](#_Toc230890018)

[**Fig. S12. Prevalence of *S. aureus* in dairy cow mastitis: a meta-analysis and subgroup analysis by continent.**	56](#_Toc230890019)

[**Fig. S13. Prevalence of *S. aureus* in dairy cow mastitis: a meta-analysis and subgroup analysis by climate.**	56](#_Toc230890020)

[**Fig. S14. Prevalence of *S. aureus* in dairy cow mastitis: a meta-analysis and subgroup analysis by breed purity.**	58](#_Toc230890021)

[**Fig. S15. Prevalence of *S. aureus* in dairy cow mastitis: a meta-analysis and subgroup analysis by** **intensification.**	59](#_Toc230890022)

[**Fig. S16. Prevalence of *Streptococcus* spp. in dairy cow mastitis: a meta-analysis and subgroup analysis by country.**	60](#_Toc230890023)

[**Fig. S17. Prevalence of *Streptococcus* spp. in dairy cow mastitis: a meta-analysis and subgroup analysis by continent.**	61](#_Toc230890024)

[**Fig. S18. Prevalence of *Streptococcus* spp. in dairy cow mastitis: a meta-analysis and subgroup analysis by climate.**	62](#_Toc230890025)

[**Fig. S19. Prevalence of *Streptococcus* spp. in dairy cow mastitis: a meta-analysis and subgroup analysis by breed purity.**	63](#_Toc230890026)

[**Fig. S20. Prevalence of *Streptococcus* spp. in dairy cow mastitis: a meta-analysis and subgroup analysis by** **intensification.**	64](#_Toc230890027)

[**Fig. S21. Prevalence of *E. coli* in dairy cow mastitis: a meta-analysis and subgroup analysis by country.**	65](#_Toc230890028)

[**Fig. S22. Prevalence of *E. coli* in dairy cow mastitis: a meta-analysis and subgroup analysis by continent.**	66](#_Toc230890029)

[**Fig. S23. Prevalence of *E. coli* in dairy cow mastitis: a meta-analysis and subgroup analysis by climate.**	67](#_Toc230890030)

[**Fig. S24. Prevalence of *E. coli* in dairy cow mastitis: a meta-analysis and subgroup analysis by breed purity.**	68](#_Toc230890031)

[**Fig. S25. Prevalence of *E. coli* in dairy cow mastitis: a meta-analysis and subgroup analysis by** **intensification.**	69](#_Toc230890032)

[**Fig. S26. Prevalence of CNS in dairy cow mastitis: a meta-analysis and subgroup analysis by country.**	70](#_Toc230890033)

[**Fig. S27. Prevalence of CNS in dairy cow mastitis: a meta-analysis and subgroup analysis by continent.**	71](#_Toc230890034)

[**Fig. S28. Prevalence of CNS in dairy cow mastitis: a meta-analysis and subgroup analysis by climate.**	72](#_Toc230890035)

[**Fig. S29. Prevalence of CNS in dairy cow mastitis: a meta-analysis and subgroup analysis by breed purity.**	73](#_Toc230890036)

[**Fig. S30. Prevalence of CNS in dairy cow mastitis: a meta-analysis and subgroup analysis by** **intensification.**	74](#_Toc230890037)

[**Fig. S31. Prevalence of *Klebsiella* spp. in dairy cow mastitis: a meta-analysis and subgroup analysis by country.**	75](#_Toc230890038)

[**Fig. S32. Prevalence of *Klebsiella* spp. in dairy cow mastitis: a meta-analysis and subgroup analysis by continent.**	76](#_Toc230890039)

[**Fig. S33. Prevalence of *Klebsiella* spp. in dairy cow mastitis: a meta-analysis and subgroup analysis by climate.**	77](#_Toc230890040)

[**Fig. S34. Prevalence of *Klebsiella* spp. in dairy cow mastitis: a meta-analysis and subgroup analysis by breed purity.**	78](#_Toc230890041)

[**Fig. S35. Prevalence of *Klebsiella* spp. in dairy cow mastitis: a meta-analysis and subgroup analysis by** **intensification.**	79](#_Toc230890042)

[**Fig. S36. Spatiotemporal distribution of *Enterococcus* spp. causing dairy cow mastitis (2000-2025).**	80](#_Toc230890043)

[**Fig. S37. Spatiotemporal distribution of *Pseudomonas* spp. causing dairy cow mastitis (2000-2025).**	81](#_Toc230890044)

[**Fig. S38. Spatiotemporal distribution of *Yeast* causing dairy cow mastitis (2000-2025).**	82](#_Toc230890045)

[**Fig. S39. Spatiotemporal distribution of *Bacillus* spp. causing dairy cow mastitis (2000-2025).**	83](#_Toc230890046)

[**Fig. S40. Spatiotemporal distribution of *Corynebacterium* spp. causing dairy cow mastitis (2000-2025).**	84](#_Toc230890047)

[**Fig. S41. Spatiotemporal distribution of *Mycoplasma* spp. causing dairy cow mastitis (2000-2025).**	85](#_Toc230890048)

[**Fig. S42. Spatiotemporal distribution of *Trueperella* spp. causing dairy cow mastitis (2000-2025).**	86](#_Toc230890049)

[**Fig. S43. Spatiotemporal distribution of *Enterobacter* spp. causing dairy cow mastitis (2000-2025).**	87](#_Toc230890050)

[**Fig. S44. Spatiotemporal distribution of *Proteus* spp. causing dairy cow mastitis (2000-2025).**	88](#_Toc230890051)

[**Fig. S45. Spatiotemporal distribution of *Lactococcus* spp. causing dairy cow mastitis (2000-2025).**	89](#_Toc230890052)

**Table S1. PRISMA 2020 checklist.**

| **Section and Topic** | **Item #** | **Checklist item** | **Location where item is reported** |
| --- | --- | --- | --- |
| **TITLE** | | |  |
| Title | 1 | Identify the report as a systematic review. | Page 1 |
| **ABSTRACT** | | |  |
| Abstract | 2 | See the PRISMA 2020 for Abstracts checklist. | Page 2-3 |
| **INTRODUCTION** | | |  |
| Rationale | 3 | Describe the rationale for the review in the context of existing knowledge. | Page 4-5 |
| Objectives | 4 | Provide an explicit statement of the objective(s) or question(s) the review addresses. | Page 5-6 |
| **METHODS** | | |  |
| Eligibility criteria | 5 | Specify the inclusion and exclusion criteria for the review and how studies were grouped for the syntheses. | Pages 6–7; Supplementary Material, Table S4 |
| Information sources | 6 | Specify all databases, registers, websites, organisations, reference lists and other sources searched or consulted to identify studies. Specify the date when each source was last searched or consulted. | Page 6; Supplementary Material, Table S2 |
| Search strategy | 7 | Present the full search strategies for all databases, registers and websites, including any filters and limits used. | Page 6; Supplementary Material, Table S2 |
| Selection process | 8 | Specify the methods used to decide whether a study met the inclusion criteria of the review, including how many reviewers screened each record and each report retrieved, whether they worked independently, and if applicable, details of automation tools used in the process. | Pages 6–7; Fig. 1; Supplementary Material, Table S4 |
| Data collection process | 9 | Specify the methods used to collect data from reports, including how many reviewers collected data from each report, whether they worked independently, any processes for obtaining or confirming data from study investigators, and if applicable, details of automation tools used in the process. | Page 7; Supplementary Material, Table S9 |
| Data items | 10a | List and define all outcomes for which data were sought. Specify whether all results that were compatible with each outcome domain in each study were sought (e.g. for all measures, time points, analyses), and if not, the methods used to decide which results to collect. | Pages 6–7; Supplementary Material, Table S9 |
|  | 10b | List and define all other variables for which data were sought (e.g. participant and intervention characteristics, funding sources). Describe any assumptions made about any missing or unclear information. | Pages 6–7; Supplementary Material, Table S9 |
| Study risk of bias assessment | 11 | Specify the methods used to assess risk of bias in the included studies, including details of the tool(s) used, how many reviewers assessed each study and whether they worked independently, and if applicable, details of automation tools used in the process. | Pages 7–8; Supplementary Material, Tables S5–S8 |
| Effect measures | 12 | Specify for each outcome the effect measure(s) (e.g. risk ratio, mean difference) used in the synthesis or presentation of results. | Pages 8–9 |
| Synthesis methods | 13a | Describe the processes used to decide which studies were eligible for each synthesis (e.g. tabulating the study intervention characteristics and comparing against the planned groups for each synthesis (item #5)). | Pages 5–8; Supplementary Material, Tables S3, S4 and S9 |
|  | 13b | Describe any methods required to prepare the data for presentation or synthesis, such as handling of missing summary statistics, or data conversions. | Pages 6–8; Supplementary Material, Table S9 |
|  | 13c | Describe any methods used to tabulate or visually display results of individual studies and syntheses. | Pages 8–22; Figs. 2–5; Tables 1–5; Supplementary Material, Tables S10–S22 and Figs. S1–S45 |
|  | 13d | Describe any methods used to synthesize results and provide a rationale for the choice(s). If meta-analysis was performed, describe the model(s), method(s) to identify the presence and extent of statistical heterogeneity, and software package(s) used. | Pages 8–9 |
|  | 13e | Describe any methods used to explore possible causes of heterogeneity among study results (e.g. subgroup analysis, meta-regression). | Pages 8–9 |
|  | 13f | Describe any sensitivity analyses conducted to assess robustness of the synthesized results. | Pages 8–9 |
| Reporting bias assessment | 14 | Describe any methods used to assess risk of bias due to missing results in a synthesis (arising from reporting biases). | Page 9; Supplementary Material, Table S10 and Figs. S1–S5 |
| Certainty assessment | 15 | Describe any methods used to assess certainty (or confidence) in the body of evidence for an outcome. | Page 7; Supplementary Material, Tables S5–S8 |
| **RESULTS** | | |  |
| Study selection | 16a | Describe the results of the search and selection process, from the number of records identified in the search to the number of studies included in the review, ideally using a flow diagram. | Page 9; Fig. 1 |
|  | 16b | Cite studies that might appear to meet the inclusion criteria, but which were excluded, and explain why they were excluded. | Page 9; Fig. 1 |
| Study characteristics | 17 | Cite each included study and present its characteristics. | Pages 9–11; Supplementary Material, Table S3 |
| Risk of bias in studies | 18 | Present assessments of risk of bias for each included study. | Page 10; Supplementary Material, Tables S5–S8 |
| Results of individual studies | 19 | For all outcomes, present, for each study: (a) summary statistics for each group (where appropriate) and (b) an effect estimate and its precision (e.g. confidence/credible interval), ideally using structured tables or plots. | Tables 1–5; Supplementary Material, Tables S13–S22 and Figs. S11–S45 |
| Results of syntheses | 20a | For each synthesis, briefly summarise the characteristics and risk of bias among contributing studies. | Pages 9–22; Supplementary Material, Tables S5–S12 |
|  | 20b | Present results of all statistical syntheses conducted. If meta-analysis was done, present for each the summary estimate and its precision (e.g. confidence/credible interval) and measures of statistical heterogeneity. If comparing groups, describe the direction of the effect. | Pages 11–22; Tables 1–5; Supplementary Material, Tables S10–S22 and Figs. S1–S45 |
|  | 20c | Present results of all investigations of possible causes of heterogeneity among study results. | Pages 15–22; Tables 1–5; Supplementary Material, Figs. S11–S35 |
|  | 20d | Present results of all sensitivity analyses conducted to assess the robustness of the synthesized results. | Pages 12–13; Supplementary Material, Tables S11 and S12 |
| Reporting biases | 21 | Present assessments of risk of bias due to missing results (arising from reporting biases) for each synthesis assessed. | Pages 12–13; Supplementary Material, Table S10 and Figs. S1–S5 |
| Certainty of evidence | 22 | Present assessments of certainty (or confidence) in the body of evidence for each outcome assessed. | Page 10; Supplementary Material, Tables S5–S8 and S12 |
| **DISCUSSION** | | |  |
| Discussion | 23a | Provide a general interpretation of the results in the context of other evidence. | Page 22-31 |
|  | 23b | Discuss any limitations of the evidence included in the review. | Page 30-31 |
|  | 23c | Discuss any limitations of the review processes used. | Page 30-31 |
|  | 23d | Discuss implications of the results for practice, policy, and future research. | Page 24-32 |
| **OTHER INFORMATION** | | |  |
| Registration and protocol | 24a | Provide registration information for the review, including register name and registration number, or state that the review was not registered. | Not applicable |
|  | 24b | Indicate where the review protocol can be accessed, or state that a protocol was not prepared. | Not applicable |
|  | 24c | Describe and explain any amendments to information provided at registration or in the protocol. | Not applicable |
| Support | 25 | Describe sources of financial or non-financial support for the review, and the role of the funders or sponsors in the review. | Funding section |
| Competing interests | 26 | Declare any competing interests of review authors. | Competing interests section |
| Availability of data, code and other materials | 27 | Report which of the following are publicly available and where they can be found: template data collection forms; data extracted from included studies; data used for all analyses; analytic code; any other materials used in the review. | Data availability section; Supplementary Material, Tables S2–S22 and Figs. S1–S45 |

*From:* Page MJ, McKenzie JE, Bossuyt PM, Boutron I, Hoffmann TC, Mulrow CD, et al. The PRISMA 2020 statement: an updated guideline for reporting systematic reviews. BMJ 2021;372:n71. doi: 10.1136/bmj.n71.

This work is licensed under CC BY 4.0. To view a copy of this license, visit <https://creativecommons.org/licenses/by/4.0/>

**Table S2. Literature search syntax for the pathogen spectrum of dairy cow mastitis.**

| Database | Website | Search strategy |
| --- | --- | --- |
| PubMed | https://pubmed.ncbi.nlm.nih.gov/ | ((("Epidemiology"[MeSH Terms] OR "Seroepidemiologic Studies"[MeSH Terms] OR "Prevalence"[MeSH Terms] OR "Risk assessment"[MeSH Terms] OR "Incidence"[MeSH Terms] OR "Morbidity"[MeSH Terms] OR "Surveys and Questionnaires"[MeSH Terms] OR ("positivity rate"[Title/Abstract] OR "prevalence trend"[Title/Abstract] OR "Health Risk Assessment"[Title/Abstract] OR "Period"[Title/Abstract] OR "morbidity rate"[Title/Abstract] OR "occurrence"[Title/Abstract] OR "characterization"[Title/Abstract] OR "survey"[Title/Abstract] OR "investigation"[Title/Abstract] OR "rate"[Title/Abstract])) AND ("Mastitis"[MeSH Terms] OR "Staphylococcus aureus"[MeSH Terms] OR "Streptococcus agalactiae"[MeSH Terms] OR "Mycoplasma bovis"[MeSH Terms] OR "Escherichia coli"[MeSH Terms] OR "Klebsiella pneumoniae"[MeSH Terms] OR ("Causative bacteria"[Title/Abstract] OR "Source infection"[Title/Abstract] OR "Atypical streptococci"[Title/Abstract] OR "Pathogen"[Title/Abstract] OR "Etiology"[Title/Abstract] OR "Risk factor"[Title/Abstract] OR "streptococcus spp"[Title/Abstract])) AND ("Cattle"[MeSH Terms] OR ("Dairy cow"[Title/Abstract] OR "Bovine"[Title/Abstract] OR "Cow"[Title/Abstract] OR "Calf"[Title/Abstract] OR "Dairy Cattle"[Title/Abstract] OR "Holstein"[Title/Abstract] OR "Jersey"[Title/Abstract] OR "Brown Swiss"[Title/Abstract] OR "Ayrshire"[Title/Abstract] OR "Guernsey"[Title/Abstract] OR "Red Holstein"[Title/Abstract] OR "Danish Red"[Title/Abstract] OR "Norwegian Red"[Title/Abstract])) AND ("Mastitis"[MeSH Terms] OR ("Clinical bovine mastitis"[Title/Abstract] OR "bovine mastitis"[Title/Abstract] OR "Cow mastitis"[Title/Abstract] OR "Mastitis"[Title/Abstract] OR "Bovine"[Title/Abstract] OR "BM"[Title/Abstract] OR "Bovine udder"[Title/Abstract]))) NOT "human"[Title/Abstract]) AND (2000:2025[pdat]) |
| Web of Science | http://www.webofscience.com/ | #1 TI OR AB："positivity rate" OR "prevalence trend" OR "Health Risk Assessment" OR "Period" OR "morbidity rate" OR "occurrence" OR "characterization" OR "survey" OR "investigation" OR "rate" OR"Epidemiology" OR "Seroepidemiologic Studies" OR "Prevalence" OR "Risk assessment" OR "Incidence" OR "Morbidity" OR "Surveys and Questionnaires"  #2 TI OR AB："Causative bacteria" OR "Source infection" OR "Atypical streptococci" OR "Pathogen" OR "Etiology" OR "Risk factor" OR "Streptococcus spp." OR "Mastitis" OR "Staphylococcus aureus" OR "Streptococcus agalactiae" OR "Mycoplasma bovis" OR "Escherichia coli" OR "Klebsiella pneumoniae"  #3 TI OR AB："Dairy cow" OR "Bovine" OR "Cow" OR "Calf" OR "Dairy Cattle" OR "Holstein" OR "Jersey" OR "Brown Swiss" OR "Ayrshire" OR "Guernsey" OR "Red Holstein" OR "Danish Red" OR "Norwegian Red" OR "Cattle"  #4TI OR AB："Clinical bovine mastitis" OR "bovine mastitis" OR "Cow mastitis" OR "Mastitis" OR "Bovine" OR "BM" OR "Bovine udder" OR "Mastitis" |
| Embase | https://www.embase.com/ | #1(('epidemiology'/exp OR 'seroepidemiologic studies'/exp OR 'prevalence'/exp OR 'risk assessment'/exp OR 'incidence'/exp OR 'morbidity'/exp OR 'surveys'/exp) AND 'questionnaires'/exp OR 'positivity rate':ab,ti OR 'prevalence trend':ab,ti OR 'health risk assessment':ab,ti OR 'period':ab,ti OR 'morbidity rate':ab,ti OR 'occurrence':ab,ti OR 'characterization':ab,ti OR 'survey':ab,ti OR 'investigation':ab,ti OR 'rate':ab,ti) AND [2000-2025]/py  #2('mastitis'/exp OR 'staphylococcus aureus'/exp OR 'streptococcus agalactiae'/exp OR 'mycoplasma bovis'/exp OR 'escherichia coli'/exp OR 'klebsiella pneumoniae'/exp OR 'causative bacteria':ab,ti OR 'source infection':ab,ti OR 'atypical streptococci':ab,ti OR 'pathogen':ab,ti OR 'etiology':ab,ti OR 'risk factor':ab,ti OR 'streptococcus spp.':ab,ti) AND [2000-2025]/py  #3('cattle'/exp OR 'dairy cow':ab,ti OR 'bovine':ab,ti OR 'cow':ab,ti OR 'calf':ab,ti OR 'dairy cattle':ab,ti OR 'holstein':ab,ti OR 'jersey':ab,ti OR 'brown swiss':ab,ti OR 'ayrshire':ab,ti OR 'guernsey':ab,ti OR 'red holstein':ab,ti OR 'danish red':ab,ti OR 'norwegian red':ab,ti) AND [2000-2025]/py  #4('mastitis'/exp OR 'clinical bovine mastitis':ab,ti OR 'bovine mastitis':ab,ti OR 'cow mastitis':ab,ti OR 'mastitis':ab,ti OR 'bovine':ab,ti OR 'bm':ab,ti OR 'bovine udder':ab,ti) AND [2000-2025]/py  #5english:la AND [2000-2025]/py  #6 #1AND #2 AND #3 AND #4 AND #5 |

**Table S3. Included literature and source information.**

| **Study** | **Country** | **Database** | **ID** |
| --- | --- | --- | --- |
| [1] | India | Web of science | WOS:000786819600001 |
| [2] | China | Web of science | WOS:000878478700001 |
| [3] | India | Web of science | WOS:000291044100020 |
| [4] | Algeria | PubMed | 31356718 |
| [5] | Vietnam | PubMed | 37235152 |
| [6] | Vietnam | PubMed | 37155534 |
| [7] | China | PubMed | 40604415 |
| [8] | China | PubMed | 34511943 |
| [9] | China | Web of science | WOS:000726937400003 |
| [10] | China | PubMed | 30940606 |
| [11] | China | Web of science | WOS:000366247100020 |
| [12] | Kosovo | PubMed | 27801370 |
| [13] | China | PubMed | 27771092 |
| [14] | Australia | PubMed | 34805335 |
| [15] | India | PubMed | 30790157 |
| [16] | Iran | PubMed | 25568683 |
| [17] | United States | PubMed | 24119795 |
| [18] | China | PubMed | 39595342 |
| [19] | Germany | PubMed | 33153084 |
| [20] | Egypt | Web of science | WOS:001338554500021 |
| [21] | China | PubMed | 35758746 |
| [22] | China | PubMed | 26276706 |
| [23] | Bangladesh | PubMed | 37533458 |
| [24] | Serbia | PubMed | 36009946 |
| [25] | Egypt | PubMed | 34206268 |
| [26] | China | PubMed | 30157847 |
| [27] | Tunisia | PubMed | 31534954 |
| [28] | Tunisia | PubMed | 29757079 |
| [29] | France | PubMed | 38096167 |
| [30] | Finland | Web of science | WOS:000391351600042 |
| [31] | Germany | PubMed | 36297146 |
| [32] | India | Web of science | WOS:000477711500002 |
| [33] | Iran | PubMed | 26973764 |
| [34] | India | PubMed | 38465323 |
| [35] | China | PubMed | 33246609 |
| [36] | China | PubMed | 24005178 |
| [37] | India | Web of science | WOS:000914329100018 |
| [38] | Ecuador | PubMed | 36548820 |
| [39] | Brazil | PubMed | 39180074 |
| [40] | India | PubMed | 28824577 |
| [41] | China | PubMed | 28434736 |
| [42] | Canada | PubMed | 18349229 |
| [43] | Canada | PubMed | 26686728 |
| [44] | Bangladesh | PubMed | 34438713 |
| [45] | Nepal | PubMed | 41018127 |
| [46] | Ethiopia | PubMed | 37025105 |
| [47] | China | Web of science | WOS:001098206200001 |
| [48] | Ethiopia | PubMed | 35996153 |
| [49] | Iran | Web of science | WOS:000432327500014 |
| [50] | Bosnia and Herzegovina | Web of science | WOS:000509758800099 |
| [51] | Romania | Web of science | WOS:001287832700001 |
| [52] | China | PubMed | 38614437 |
| [53] | Poland | PubMed | 38734661 |
| [54] | China | PubMed | 32810702 |
| [55] | China | Web of science | WOS:001360378300002 |
| [56] | China | PubMed | 36072389 |
| [57] | Bangladesh | Web of science | WOS:001035824700002 |
| [58] | Ethiopia | Web of science | WOS:000674122300001 |
| [59] | China | PubMed | 39582476 |
| [60] | China | PubMed | 36374018 |
| [61] | China | Web of science | WOS:000807736400018 |
| [62] | United States | PubMed | 22916896 |
| [63] | China | Web of science | WOS:000630014700090 |
| [64] | Mexico | Web of science | WOS:000635701800027 |
| [65] | China | PubMed | 35522690 |
| [66] | Egypt | Web of science | WOS:001480412600001 |
| [67] | China | Web of science | WOS:000711075700001 |
| [68] | Thailand | PubMed | 39077446 |
| [69] | Poland | PubMed | 30989140 |
| [70] | Brazil | PubMed | 36964326 |
| [71] | Pakistan | Web of science | WOS:001076084900001 |
| [72] | China | PubMed | 41087872 |
| [73] | Brazil | PubMed | 28671260 |
| [74] | Iran | PubMed | 31096003 |
| [75] | Egypt | Web of science | WOS:000657256300020 |
| [76] | Jordan | Web of science | WOS:001121714400004 |
| [77] | United Kingdom | PubMed | 28716189 |
| [78] | India | Web of science | WOS:000753493900010 |
| [79] | Uganda | Web of science | WOS:000319654700139 |
| [80] | China | PubMed | 31887413 |
| [81] | China | Web of science | WOS:000987293900001 |
| [82] | China | Web of science | WOS:000519992900038 |
| [83] | Ethiopia | Web of science | WOS:000270226200065 |
| [84] | China | Web of science | WOS:001234881800001 |
| [85] | Bangladesh | Web of science | WOS:001409564300005 |
| [86] | Tunisia | Web of science | WOS:000698422200004 |
| [87] | Mexico | PubMed | 26255183 |
| [88] | Egypt | PubMed | 32801573 |
| [89] | China | Web of science | WOS:000919499400001 |
| [90] | China | PubMed | 25294597 |
| [91] | Nepal | PubMed | 34233667 |
| [92] | China | Web of science | WOS:000558156400004 |
| [93] | China | Web of science | WOS:000308513800007 |
| [94] | Norway | PubMed | 37264425 |
| [95] | China | Web of science | WOS:000390631000004 |
| [96] | Malaysia | PubMed | 35804578 |
| [97] | Ethiopia | PubMed | 28935553 |
| [98] | Bangladesh | Web of science | WOS:001345021700001 |
| [99] | China | Web of science | WOS:000940708300001 |
| [100] | United Kingdom | PubMed | 19447987 |
| [101] | Egypt | Web of science | WOS:001444876500002 |
| [102] | Hungary | PubMed | 15351035 |
| [103] | Germany | PubMed | 37764906 |
| [104] | Iran | Web of science | WOS:000333138000039 |
| [105] | Australia | PubMed | 34955276 |
| [106] | United States | Web of science | WOS:001535763000001 |
| [107] | South Africa | Web of science | WOS:000734044100001 |
| [108] | Canada | PubMed | 22981579 |
| [109] | Ethiopia | PubMed | 35028299 |
| [110] | Bangladesh | Web of science | WOS:001565156700002 |
| [111] | India | Web of science | WOS:000411344700001 |
| [112] | China | Web of science | WOS:000982776200001 |
| [113] | China | Web of science | WOS:000716500300001 |
| [114] | Japan | PubMed | 17917373 |
| [115] | Iran | Web of science | WOS:000460487100010 |
| [116] | Pakistan | PubMed | 29325865 |
| [117] | Algeria | PubMed | 31338728 |
| [118] | Australia | Web of science | WOS:000416127200002 |
| [119] | Ethiopia | PubMed | 32864088 |
| [120] | China | PubMed | 40266927 |
| [121] | Ethiopia | PubMed | 34676155 |
| [122] | Egypt | PubMed | 33601806 |
| [123] | Sri Lanka | PubMed | 34482972 |
| [124] | China | PubMed | 39770845 |
| [125] | Algeria | Web of science | WOS:000351168800022 |
| [126] | Pakistan | Web of science | WOS:000813941300006 |
| [127] | Poland | Web of science | WOS:000735317700001 |
| [128] | Canada | PubMed | 29397171 |
| [129] | Pakistan | Web of science | WOS:001256850300121 |
| [130] | Bangladesh | Web of science | WOS:000729373100005 |
| [131] | Egypt | Web of science | WOS:001447212500001 |
| [132] | Thailand | Web of science | WOS:000428760500011 |
| [133] | Estonia | PubMed | 28601455 |
| [134] | Pakistan | Embase | 10.29261/pakvetj/2019.049 |
| [135] | India | Embase | https://doi.org/10.22207/JPAM.11.1.41 |
| [136] | Iraq | Embase | 10.33899/ijvs.2021.129934.1702 |
| [137] | Pakistan | Embase | 10.29261/pakvetj/2022.078 |

**Table S4. Inclusion and exclusion criteria for studies on the pathogen spectrum of dairy cow mastitis.**

| **Inclusion criteria** | **Exclusion criteria** |
| --- | --- |
| Epidemiological studies of pathogenic microorganisms causing bovine mastitis | Pathogen not specified |
| Cross-sectional studies on pathogenic microorganisms associated with bovine mastitis | Laboratory challenge or molecular-mechanism studies |
| Investigations of bovine mastitis caused by pathogenic microorganisms | Articles reporting only isolation/identification of pathogens |
| Studies on pathogenic microorganisms in bovine mastitis across different countries or regions | Non-research articles (e.g., reviews, editorials, comments) |
| Detection and identification of pathogenic microorganisms in bovine mastitis | Data unavailable or lacking epidemiological information |
| Studies on pathogenic microorganisms in bovine mastitis over different time periods | Full text unobtainable |
|  | Non-English papers |
|  | No geographic information provided |
|  | Samples collected before 2000 |
|  | Individual-case studies |
|  | Studies not conducted in dairy cows |
|  | Case reports |

**Table S5. Risk of bias assessment based on STROBE criteria.**

| **Bias type** | **Low risk(score=1)** | **Moderate risk(score=2)** | **High risk(score=3)** |
| --- | --- | --- | --- |
| Sample representativeness | 1. Clearly defined mastitis cases (CM or SCM) quantity  2. Use of consecutive or random sampling | 1. Clearly defined mastitis cases (CM or SCM) quantity  2. Sample comes from a large farm, convenience sampling, or unclear sampling framework | Denominator is healthy cows, quarter numbers, or cow numbers, or mastitis status not distinguished |
| Sample Size | 1. Report sample size calculation or ≥200 samples | 1. Not calculated 100-199 samples  2. Calculated but not reached the calculated sample size | 1. <100 cases with no calculation basis  2. No sample size calculation, potentially leading to insufficient sample size; only part of the population studied, reason unclear |
| Outcome Assessment | 1. ≥2 types of phenotypic or molecular methods verified (e.g., culture + PCR/MALDI-TOF) or a single recognized method | 1. Only one method and not validated  2. Use of reliable data sources (such as administrative databases), but with certain limitations | 1. Method unclear or obviously inappropriate  2. Only estimation |
| Analytical  methods | Analysis method suitable for sample type, detailed description, includes robust statistical methods | Analysis method suitable for sample type, but did not consider all relevant confounding factors | No details provided on analysis methods; results may have reproducibility issues |

**Table S6. Bias assessment results for included studies based on STROBE criteria.**

| **Study** | **Author** | **Publication**  **year** | **Sample population** | **Sample size** | **Outcome assessment** | **Analytical methods** | **Total**  **score** |
| --- | --- | --- | --- | --- | --- | --- | --- |
| [1] | U. Brahma | 2022 | 2 | 1 | 1 | 2 | 6 |
| [2] | K. Li | 2022 | 2 | 2 | 1 | 2 | 7 |
| [3] | R. Kumar | 2011 | 2 | 2 | 1 | 2 | 7 |
| [4] | N. Zaatout | 2019 | 2 | 1 | 1 | 3 | 7 |
| [5] | T. T. My | 2023 | 2 | 1 | 1 | 1 | 5 |
| [6] | T. T. My | 2023 | 2 | 1 | 1 | 2 | 6 |
| [7] | J. Wang | 2025 | 2 | 1 | 1 | 1 | 5 |
| [8] | J. Shen | 2021 | 2 | 1 | 1 | 2 | 6 |
| [9] | Y. Guo | 2021 | 2 | 2 | 1 | 1 | 6 |
| [10] | X. Y. Tian | 2019 | 2 | 1 | 1 | 2 | 6 |
| [11] | Y. Ding | 2016 | 2 | 1 | 1 | 2 | 6 |
| [12] | I. Mehmeti | 2016 | 2 | 2 | 1 | 3 | 8 |
| [13] | D. Wang | 2016 | 2 | 2 | 1 | 2 | 7 |
| [14] | H. Al-Harbi | 2021 | 2 | 2 | 1 | 2 | 7 |
| [15] | P. S. Bhutia | 2019 | 2 | 1 | 1 | 2 | 6 |
| [16] | A. Ebrahimi | 2013 | 2 | 2 | 2 | 3 | 9 |
| [17] | L. Oliveira | 2013 | 1 | 1 | 1 | 1 | 4 |
| [18] | Y. Huang | 2024 | 2 | 2 | 1 | 2 | 7 |
| [19] | A. Schmenger | 2020 | 1 | 1 | 1 | 1 | 4 |
| [20] | A. M. Abdalhamed | 2023 | 2 | 3 | 1 | 2 | 8 |
| [21] | Z. Zhang | 2022 | 1 | 1 | 1 | 2 | 5 |
| [22] | J. Xu | 2015 | 2 | 1 | 1 | 2 | 6 |
| [23] | J. Hassan | 2023 | 2 | 3 | 1 | 2 | 8 |
| [24] | D. Tomanić | 2022 | 3 | 3 | 1 | 2 | 9 |
| [25] | N. K. Abd El-Aziz | 2021 | 2 | 1 | 1 | 2 | 6 |
| [26] | J. Du | 2018 | 2 | 1 | 1 | 2 | 6 |
| [27] | A. Klibi | 2019 | 2 | 1 | 1 | 2 | 6 |
| [28] | M. Saidani | 2018 | 1 | 1 | 1 | 2 | 5 |
| [29] | O. Salat | 2023 | 2 | 1 | 2 | 3 | 8 |
| [30] | S. Taponen | 2017 | 1 | 1 | 1 | 1 | 4 |
| [31] | F. Fredebeul-Krein | 2022 | 2 | 1 | 2 | 1 | 6 |
| [32] | N. S.  Karabasanavar | 2018 | 2 | 3 | 1 | 3 | 9 |
| [33] | M. Mohsenzadeh | 2015 | 2 | 1 | 1 | 1 | 5 |
| [34] | B. P. Katira | 2024 | 2 | 2 | 1 | 1 | 6 |
| [35] | J. Cheng | 2021 | 2 | 1 | 1 | 1 | 5 |
| [36] | L. Wang | 2013 | 2 | 2 | 1 | 2 | 7 |
| [37] | S. Panigrahi | 2022 | 2 | 3 | 1 | 2 | 8 |
| [38] | M. P. Huilca-Ibarra | 2022 | 1 | 1 | 1 | 2 | 5 |
| [39] | F. F. Guimarães | 2024 | 2 | 1 | 1 | 2 | 6 |
| [40] | S. Mahato | 2017 | 2 | 2 | 1 | 2 | 7 |
| [41] | J. Gao | 2017 | 1 | 1 | 1 | 2 | 5 |
| [42] | R. G. M. Olde Riekerink | 2018 | 1 | 1 | 1 | 1 | 4 |
| [43] | L. J. Levison | 2016 | 1 | 1 | 1 | 1 | 4 |
| [44] | S. Singha | 2021 | 1 | 2 | 1 | 1 | 5 |
| [45] | S. Adhikari | 2025 | 2 | 2 | 1 | 2 | 7 |
| [46] | N. Feyissa | 2023 | 1 | 3 | 1 | 1 | 6 |
| [47] | W. Cao | 2023 | 1 | 1 | 2 | 2 | 6 |
| [48] | M. Yimana | 2022 | 1 | 2 | 1 | 1 | 5 |
| [49] | M. Imandar | 2018 | 2 | 1 | 1 | 2 | 6 |
| [50] | Seric-Haracic | 2019 | 2 | 2 | 2 | 3 | 9 |
| [51] | I. Hutu | 2024 | 1 | 1 | 1 | 2 | 5 |
| [52] | L. Zhu | 2024 | 1 | 1 | 1 | 2 | 5 |
| [53] | A. Dobrut | 2024 | 1 | 2 | 1 | 2 | 6 |
| [54] | Y. Liu | 2020 | 1 | 1 | 1 | 2 | 5 |
| [55] | M. Wusiman | 2025 | 1 | 1 | 1 | 2 | 5 |
| [56] | J. Liu | 2022 | 2 | 2 | 1 | 2 | 7 |
| [57] | Z. Farzana | 2023 | 2 | 2 | 1 | 1 | 6 |
| [58] | D. T. Tegegne | 2021 | 1 | 2 | 2 | 2 | 7 |
| [59] | K. Cai | 2025 | 1 | 1 | 1 | 3 | 6 |
| [60] | S. Fu | 2022 | 1 | 2 | 1 | 2 | 6 |
| [61] | S. Song | 2022 | 1 | 1 | 1 | 3 | 6 |
| [62] | K. L. Anderson | 2012 | 1 | 1 | 1 | 1 | 4 |
| [63] | L. Lin | 2021 | 2 | 1 | 1 | 2 | 6 |
| [64] | Y. Mora-Hernández | 2021 | 1 | 2 | 3 | 2 | 8 |
| [65] | G. Han | 2022 | 1 | 1 | 1 | 2 | 5 |
| [66] | H. Fayed | 2025 | 2 | 2 | 1 | 2 | 7 |
| [67] | J. Zeng | 2022 | 1 | 2 | 1 | 2 | 6 |
| [68] | A. Camsing | 2024 | 2 | 3 | 1 | 2 | 8 |
| [69] | H. Różańska | 2019 | 2 | 1 | 1 | 2 | 6 |
| [70] | T. A. O. Sierra | 2023 | 2 | 1 | 1 | 2 | 6 |
| [71] | M. Shahid | 2023 | 2 | 2 | 1 | 2 | 7 |
| [72] | Y. Wang | 2025 | 2 | 1 | 1 | 2 | 6 |
| [73] | A. R. E. O. Xavier | 2017 | 2 | 1 | 1 | 2 | 6 |
| [74] | F. Fazel | 2019 | 2 | 1 | 1 | 2 | 6 |
| [75] | E. E. Abdeen | 2021 | 2 | 2 | 1 | 2 | 7 |
| [76] | Y. H. Tarazi | 2023 | 2 | 1 | 1 | 2 | 6 |
| [77] | S. C. Archer | 2017 | 1 | 1 | 1 | 1 | 4 |
| [78] | D. C. Widianingrum | 2022 | 2 | 1 | 1 | 2 | 6 |
| [79] | D. P. Kateete | 2013 | 2 | 3 | 3 | 2 | 10 |
| [80] | Z. N. Yu | 2020 | 2 | 1 | 1 | 2 | 6 |
| [81] | Z. Y. Liang | 2023 | 1 | 1 | 1 | 2 | 5 |
| [82] | Q. Ren | 2020 | 3 | 3 | 1 | 2 | 9 |
| [83] | M. Lakew | 2009 | 2 | 2 | 1 | 2 | 7 |
| [84] | J. Liu | 2024 | 2 | 1 | 1 | 2 | 6 |
| [85] | T. Chowdhury | 2025 | 2 | 2 | 1 | 2 | 7 |
| [86] | A. Maalaoui | 2021 | 1 | 2 | 1 | 2 | 6 |
| [87] | J. Olivares-Pérez | 2015 | 2 | 3 | 1 | 2 | 8 |
| [88] | R. M. Ewida | 2020 | 2 | 3 | 1 | 2 | 8 |
| [89] | T. Xu | 2023 | 2 | 2 | 1 | 2 | 7 |
| [90] | F. L. Yang | 2015 | 2 | 1 | 3 | 2 | 8 |
| [91] | A. Shrestha | 2021 | 2 | 2 | 1 | 2 | 7 |
| [92] | X. Song | 2020 | 1 | 1 | 1 | 2 | 5 |
| [93] | J. Xu | 2012 | 2 | 1 | 1 | 2 | 6 |
| [94] | M. Smistad | 2023 | 2 | 1 | 1 | 2 | 6 |
| [95] | X. Wu | 2016 | 2 | 1 | 1 | 2 | 6 |
| [96] | S. I. Saeed | 2022 | 2 | 3 | 1 | 2 | 8 |
| [97] | B. Seyoum | 2018 | 2 | 1 | 1 | 2 | 6 |
| [98] | A. Farabi | 2025 | 2 | 3 | 1 | 2 | 8 |
| [99] | X. Xie | 2023 | 2 | 1 | 1 | 2 | 6 |
| [100] | J. E. Breen | 2009 | 2 | 1 | 3 | 2 | 8 |
| [101] | M. M. Elhaig | 2024 | 2 | 3 | 1 | 2 | 8 |
| [102] | D. Kálmán | 2004 | 1 | 2 | 1 | 1 | 5 |
| [103] | I. Krebs | 2023 | 1 | 1 | 1 | 2 | 5 |
| [104] | H. Jamali | 2014 | 2 | 1 | 2 | 2 | 7 |
| [105] | R. Dyson | 2022 | 1 | 1 | 1 | 2 | 5 |
| [106] | D. R. Bruno | 2025 | 1 | 1 | 1 | 2 | 5 |
| [107] | N. G. Khasapane | 2021 | 2 | 3 | 1 | 2 | 8 |
| [108] | M.-È. Paradis | 2012 | 1 | 1 | 1 | 1 | 4 |
| [109] | N. Belay | 2022 | 2 | 3 | 1 | 2 | 8 |
| [110] | J. Ara | 2025 | 2 | 3 | 1 | 2 | 8 |
| [111] | V. Sharma | 2017 | 2 | 1 | 1 | 2 | 6 |
| [112] | Y. S. Lin | 2023 | 2 | 1 | 1 | 2 | 6 |
| [113] | S. Y. Xu | 2021 | 2 | 1 | 1 | 2 | 6 |
| [114] | H. Nagahata | 2007 | 2 | 3 | 1 | 2 | 8 |
| [115] | M. Marashifard | 2019 | 2 | 1 | 1 | 2 | 6 |
| [116] | A. I. Aqib | 2018 | 2 | 1 | 1 | 2 | 6 |
| [117] | N. Zaatout | 2020 | 2 | 1 | 1 | 2 | 6 |
| [118] | A. Al-Farha | 2017 | 2 | 1 | 1 | 2 | 6 |
| [119] | W. Girmay | 2020 | 2 | 3 | 1 | 2 | 8 |
| [120] | H. Zhao | 2025 | 2 | 1 | 1 | 1 | 5 |
| [121] | H. Fesseha | 2021 | 2 | 3 | 3 | 2 | 10 |
| [122] | H. F. Ahmed | 2018 | 2 | 1 | 1 | 2 | 6 |
| [123] | R. M. S. B. K. Ranasinghe | 2021 | 2 | 1 | 1 | 2 | 6 |
| [124] | L. Wang | 2024 | 2 | 1 | 3 | 2 | 8 |
| [125] | S. Ksouri | 2015 | 2 | 1 | 1 | 2 | 6 |
| [126] | M. A. Anwar | 2022 | 2 | 2 | 1 | 2 | 7 |
| [127] | M. Bochniarz | 2021 | 2 | 3 | 2 | 2 | 9 |
| [128] | S. A. Naqvi | 2018 | 2 | 1 | 2 | 2 | 7 |
| [129] | M. A. Shahzad | 2024 | 2 | 2 | 1 | 2 | 7 |
| [130] | M. A. S. Bag | 2021 | 2 | 3 | 1 | 2 | 8 |
| [131] | N. H. Eidaroos | 2025 | 2 | 3 | 1 | 2 | 8 |
| [132] | S. Boonyayatra | 2018 | 2 | 2 | 1 | 2 | 7 |
| [133] | A. A. E. Timonen | 2017 | 2 | 1 | 1 | 2 | 6 |
| [134] | A. Khan | 2019 | 2 | 3 | 2 | 2 | 9 |
| [135] | A. Gangwal | 2017 | 2 | 3 | 1 | 3 | 9 |
| [136] | A. A. Neamah | 2022 | 2 | 3 | 1 | 3 | 9 |
| [137] | A. Ahmed | 2022 | 2 | 1 | 1 | 2 | 6 |

**Table S7. Quality assessment checklist.**

| **No.** | **Items (Score 1 point if achieved; no points if not achieved.)** |
| --- | --- |
| **1** | Was the research objective clearly defined? |
| **2** | Was the sampling area clearly described with reference to the location? |
| **3** | Was the study period specified? |
| **4** | Was the sample type clearly described? |
| **5** | Was some form of random selection used to select the samples? |
| **6** | Were the subjects categorized by species? |
| **7** | Was the diagnostic method for the samples clearly described? |
| **8** | Were the clinical symptoms of the samples clearly described? |
| **9** | Was there a distinction between clinical and subclinical mastitis? |
| **10** | Was farm size described? |

**Table S8. Quality score and quality index score of individual contributing studies.**

| **Study** | **Author** | **Year** | **Quality score** | **Quality index score** | **Quality category** |
| --- | --- | --- | --- | --- | --- |
| [1] | U. Brahma | 2022 | 9 | 0.9 | High |
| [2] | K. Li | 2022 | 9 | 0.9 | High |
| [3] | R. Kumar | 2011 | 6 | 0.6 | Moderate |
| [4] | N. Zaatout | 2019 | 8 | 0.8 | High |
| [5] | T. T. My | 2023 | 9 | 0.9 | High |
| [6] | T. T. My | 2023 | 9 | 0.9 | High |
| [7] | J. Wang | 2025 | 9 | 0.9 | High |
| [8] | J. Shen | 2021 | 8 | 0.8 | High |
| [9] | Y. Guo | 2021 | 9 | 0.9 | High |
| [10] | X. Y. Tian | 2019 | 8 | 0.8 | High |
| [11] | Y. Ding | 2016 | 9 | 0.9 | High |
| [12] | I. Mehmeti | 2016 | 8 | 0.8 | High |
| [13] | D. Wang | 2016 | 7 | 0.7 | Moderate |
| [14] | H. Al-Harbi | 2021 | 7 | 0.7 | Moderate |
| [15] | P. S. Bhutia | 2019 | 7 | 0.7 | Moderate |
| [16] | A. Ebrahimi | 2013 | 7 | 0.7 | Moderate |
| [17] | L. Oliveira | 2013 | 7 | 0.7 | Moderate |
| [18] | Y. Huang | 2024 | 6 | 0.6 | Moderate |
| [19] | A. Schmenger | 2020 | 8 | 0.8 | High |
| [20] | A. M. Abdalhamed | 2023 | 7 | 0.7 | Moderate |
| [21] | Z. Zhang | 2022 | 8 | 0.8 | High |
| [22] | J. Xu | 2015 | 9 | 0.9 | High |
| [23] | J. Hassan | 2023 | 7 | 0.7 | Moderate |
| [24] | D. Tomanić | 2022 | 8 | 0.8 | High |
| [25] | N. K. Abd El-Aziz | 2021 | 9 | 0.9 | High |
| [26] | J. Du | 2018 | 7 | 0.7 | Moderate |
| [27] | A. Klibi | 2019 | 8 | 0.8 | High |
| [28] | M. Saidani | 2018 | 8 | 0.8 | High |
| [29] | O. Salat | 2023 | 7 | 0.7 | Moderate |
| [30] | S. Taponen | 2017 | 7 | 0.7 | Moderate |
| [31] | F. Fredebeul-Krein | 2022 | 8 | 0.8 | High |
| [32] | N. S. Karabasanavar | 2018 | 8 | 0.8 | High |
| [33] | M. Mohsenzadeh | 2015 | 7 | 0.7 | Moderate |
| [34] | B. P. Katira | 2024 | 7 | 0.7 | Moderate |
| [35] | J. Cheng | 2021 | 10 | 1 | High |
| [36] | L. Wang | 2013 | 7 | 0.7 | Moderate |
| [37] | S. Panigrahi | 2022 | 8 | 0.8 | High |
| [38] | M. P. Huilca-Ibarra | 2022 | 8 | 0.8 | High |
| [39] | F. F. Guimarães | 2024 | 9 | 0.9 | High |
| [40] | S. Mahato | 2017 | 8 | 0.8 | High |
| [41] | J. Gao | 2017 | 9 | 0.9 | High |
| [42] | R. G. M. Olde Riekerink | 2018 | 8 | 0.8 | High |
| [43] | L. J. Levison | 2016 | 8 | 0.8 | High |
| [44] | S. Singha | 2021 | 9 | 0.9 | High |
| [45] | S. Adhikari | 2025 | 9 | 0.9 | High |
| [46] | N. Feyissa | 2023 | 10 | 1 | High |
| [47] | W. Cao | 2023 | 7 | 0.7 | Moderate |
| [48] | M. Yimana | 2022 | 9 | 0.9 | High |
| [49] | M. Imandar | 2018 | 9 | 0.9 | High |
| [50] | Seric-Haracic | 2019 | 8 | 0.8 | High |
| [51] | I. Hutu | 2024 | 10 | 1 | High |
| [52] | L. Zhu | 2024 | 9 | 0.9 | High |
| [53] | A. Dobrut | 2024 | 7 | 0.7 | Moderate |
| [54] | Y. Liu | 2020 | 9 | 0.9 | High |
| [55] | M. Wusiman | 2025 | 9 | 0.9 | High |
| [56] | J. Liu | 2022 | 8 | 0.8 | High |
| [57] | Z. Farzana | 2023 | 6 | 0.6 | Moderate |
| [58] | D. T. Tegegne | 2021 | 8 | 0.8 | High |
| [59] | K. Cai | 2025 | 9 | 0.9 | High |
| [60] | S. Fu | 2022 | 8 | 0.8 | High |
| [61] | S. Song | 2022 | 9 | 0.9 | High |
| [62] | K. L. Anderson | 2012 | 7 | 0.7 | Moderate |
| [63] | L. Lin | 2021 | 8 | 0.8 | High |
| [64] | Y. Mora-Hernández | 2021 | 8 | 0.8 | High |
| [65] | G. Han | 2022 | 8 | 0.8 | High |
| [66] | H. Fayed | 2025 | 8 | 0.8 | High |
| [67] | J. Zeng | 2022 | 9 | 0.9 | High |
| [68] | A. Camsing | 2024 | 9 | 0.9 | High |
| [69] | H. Różańska | 2019 | 6 | 0.6 | Moderate |
| [70] | T. A. O. Sierra | 2023 | 7 | 0.7 | Moderate |
| [71] | M. Shahid | 2023 | 8 | 0.8 | High |
| [72] | Y. Wang | 2025 | 9 | 0.9 | High |
| [73] | A. R. E. O. Xavier | 2017 | 8 | 0.8 | High |
| [74] | F. Fazel | 2019 | 8 | 0.8 | High |
| [75] | E. E. Abdeen | 2021 | 8 | 0.8 | High |
| [76] | Y. H. Tarazi | 2023 | 8 | 0.8 | High |
| [77] | S. C. Archer | 2017 | 9 | 0.9 | High |
| [78] | D. C. Widianingrum | 2022 | 7 | 0.7 | Moderate |
| [79] | D. P. Kateete | 2013 | 9 | 0.9 | High |
| [80] | Z. N. Yu | 2020 | 9 | 0.9 | High |
| [81] | Z. Y. Liang | 2023 | 9 | 0.9 | High |
| [82] | Q. Ren | 2020 | 8 | 0.8 | High |
| [83] | M. Lakew | 2009 | 10 | 1 | High |
| [84] | J. Liu | 2024 | 9 | 0.9 | High |
| [85] | T. Chowdhury | 2025 | 7 | 0.7 | Moderate |
| [86] | A. Maalaoui | 2021 | 9 | 0.9 | High |
| [87] | J. Olivares-Pérez | 2015 | 6 | 0.6 | Moderate |
| [88] | R. M. Ewida | 2020 | 8 | 0.8 | High |
| [89] | T. Xu | 2023 | 9 | 0.9 | High |
| [90] | F. L. Yang | 2015 | 8 | 0.8 | High |
| [91] | A. Shrestha | 2021 | 10 | 1 | High |
| [92] | X. Song | 2020 | 9 | 0.9 | High |
| [93] | J. Xu | 2012 | 8 | 0.8 | High |
| [94] | M. Smistad | 2023 | 9 | 0.9 | High |
| [95] | X. Wu | 2016 | 8 | 0.8 | High |
| [96] | S. I. Saeed | 2022 | 8 | 0.8 | High |
| [97] | B. Seyoum | 2018 | 9 | 0.9 | High |
| [98] | A. Farabi | 2025 | 9 | 0.9 | High |
| [99] | X. Xie | 2023 | 9 | 0.9 | High |
| [100] | J. E. Breen | 2009 | 9 | 0.9 | High |
| [101] | M. M. Elhaig | 2024 | 7 | 0.7 | Moderate |
| [102] | D. Kálmán | 2004 | 8 | 0.8 | High |
| [103] | I. Krebs | 2023 | 9 | 0.9 | High |
| [104] | H. Jamali | 2014 | 8 | 0.8 | High |
| [105] | R. Dyson | 2022 | 9 | 0.9 | High |
| [106] | D. R. Bruno | 2025 | 7 | 0.7 | Moderate |
| [107] | N. G. Khasapane | 2021 | 7 | 0.7 | Moderate |
| [108] | M.-È. Paradis | 2012 | 7 | 0.7 | Moderate |
| [109] | N. Belay | 2022 | 9 | 0.9 | High |
| [110] | J. Ara | 2025 | 8 | 0.8 | High |
| [111] | V. Sharma | 2017 | 6 | 0.6 | Moderate |
| [112] | Y. S. Lin | 2023 | 6 | 0.6 | Moderate |
| [113] | S. Y. Xu | 2021 | 9 | 0.9 | High |
| [114] | H. Nagahata | 2007 | 6 | 0.6 | Moderate |
| [115] | M. Marashifard | 2019 | 8 | 0.8 | High |
| [116] | A. I. Aqib | 2018 | 7 | 0.7 | Moderate |
| [117] | N. Zaatout | 2020 | 9 | 0.9 | High |
| [118] | A. Al-Farha | 2017 | 7 | 0.7 | Moderate |
| [119] | W. Girmay | 2020 | 9 | 0.9 | High |
| [120] | H. Zhao | 2025 | 9 | 0.9 | High |
| [121] | H. Fesseha | 2021 | 9 | 0.9 | High |
| [122] | H. F. Ahmed | 2018 | 9 | 0.9 | High |
| [123] | R. M. S. B. K. Ranasinghe | 2021 | 10 | 1 | High |
| [124] | L. Wang | 2024 | 9 | 0.9 | High |
| [125] | S. Ksouri | 2015 | 8 | 0.8 | High |
| [126] | M. A. Anwar | 2022 | 8 | 0.8 | High |
| [127] | M. Bochniarz | 2021 | 10 | 1 | High |
| [128] | S. A. Naqvi | 2018 | 10 | 1 | High |
| [129] | M. A. Shahzad | 2024 | 8 | 0.8 | High |
| [130] | M. A. S. Bag | 2021 | 9 | 0.9 | High |
| [131] | N. H. Eidaroos | 2025 | 8 | 0.8 | High |
| [132] | S. Boonyayatra | 2018 | 9 | 0.9 | High |
| [133] | A. A. E. Timonen | 2017 | 9 | 0.9 | High |
| [134] | A. Khan | 2019 | 9 | 0.9 | High |
| [135] | A. Gangwal | 2017 | 6 | 0.6 | Moderate |
| [136] | A. A. Neamah | 2022 | 6 | 0.6 | Moderate |
| [137] | A. Ahmed | 2022 | 9 | 0.9 | High |

**Table S9. Data extraction variables from reviewed literature.**

| **Variable** | **Guidance** |
| --- | --- |
| **Title** | Title of the literature included in the research. |
| **First author** | First author of the included article. |
| **Publish year** | Year of publication for the article identified for data extraction. |
| **Year of samples collection** | Year of sample collection in the included studies. |
| **Accession number** | Unique identifier assigned to the literature. |
| **Full reference** | Complete citation of the literature in the appropriate format. |
| **Country** | Geographic location information recorded at the country level. |
| **Continent** | Continent of the sample collection region. |
| **Climate** | Climate of the sample collection area. |
| **Intensive or not** | Is the farm where samples were collected intensive. |
| **Purebred or not** | Are the dairy cows from which samples were collected purebred. |
| **Sample size** | Total number of animals tested for a specific pathogen using assay methods |
| **Positive number** | Number of animals diagnosed as positive for a specific pathogen. |
| **Detection methods** | Method used for testing (e.g., PCR, culture and biochemical identification). |

**Table S10.** **Bias in the inclusion of studies for multi-model validation.**

| **Pathogens** | **Pooled effect**  **,%** | **I² ,%** | **Heterogeneity *P*-value** | **Egger's *P*-value** | **Begg's *P*-value** | **Leave-one-out ,%** |
| --- | --- | --- | --- | --- | --- | --- |
| *S. aureus* | 19.59 | 99.2 | 0 | 0.6985 | 0.28 | 19.6 |
| *Streptococcus* spp. | 17.29 | 99.2 | 0 | 0.0037 | 0.41 | 17.3 |
| *E. coli* | 13.86 | 98.9 | 0 | 0.0186 | 0.01 | 13.9 |
| CNS | 15.49 | 99.6 | 0 | 0.0016 | 0.15 | 15.5 |
| *Klebsiella* spp. | 9.92 | 98.1 | < 0.0001 | 0.7397 | 0.32 | 9.9 |

**Table S11. Sensitivity analysis using alternative random-effects models for the five dominant pathogen categories.**

| **Pathogens** | **Model** | **Positive rate,%** | **CI lower,%** | **CI upper,%** | **Tau^2^** | **I^2^,%** |
| --- | --- | --- | --- | --- | --- | --- |
| *S. aureus* | DerSimonian-Laird | 19.59 | 17 | 22.47 | 0.5689 | 99.2 |
|  | REML | 19.52 | 15.15 | 24.8 | 1.8973 | 99.2 |
|  | Paule-Mandel | 19.52 | 15.05 | 24.94 | 1.9958 | 99.2 |
|  | REML + Hartung-Knapp | 19.52 | 14.99 | 25.03 | 1.8973 | 99.2 |
| *Streptococcus* spp. | DerSimonian-Laird | 17.29 | 15.14 | 19.66 | 0.3397 | 99.2 |
|  | REML | 16.46 | 12.99 | 20.63 | 1.1367 | 99.2 |
|  | Paule-Mandel | 16.42 | 12.87 | 20.73 | 1.2108 | 99.2 |
|  | REML + Hartung-Knapp | 16.46 | 12.84 | 20.86 | 1.1367 | 99.2 |
| *E. coli* | DerSimonian-Laird | 13.86 | 11.22 | 17 | 0.7203 | 98.9 |
|  | REML | 13.84 | 11.17 | 17.03 | 0.7455 | 98.9 |
|  | Paule-Mandel | 13.81 | 11.07 | 17.1 | 0.7934 | 98.9 |
|  | REML + Hartung-Knapp | 13.84 | 11.04 | 17.22 | 0.7455 | 98.9 |
| CNS | DerSimonian-Laird | 15.49 | 10.12 | 22.99 | 1.9408 | 99.6 |
|  | REML | 15.5 | 10.6 | 22.11 | 1.5466 | 99.6 |
|  | Paule-Mandel | 15.5 | 10.6 | 22.11 | 1.5487 | 99.6 |
|  | REML + Hartung-Knapp | 15.5 | 10.43 | 22.42 | 1.5466 | 99.6 |
| *Klebsiella* spp. | DerSimonian-Laird | 9.92 | 7.38 | 13.2 | 0.6616 | 98.1 |
|  | REML | 9.37 | 6.22 | 13.87 | 1.3136 | 98.1 |
|  | Paule-Mandel | 9.32 | 6.09 | 13.99 | 1.4163 | 98.1 |
|  | REML + Hartung-Knapp | 9.37 | 6.01 | 14.31 | 1.3136 | 98.1 |

**Table S12. Sensitivity analysis restricted to high-quality studies.**

| **Pathogens** | **Analysis** | **Number of studies, *n*** | **Positive rate,%** | **CI lower,%** | **CI upper,%** | **Tau^2^** | **I^2^,%** |
| --- | --- | --- | --- | --- | --- | --- | --- |
| *S. aureus* | All studies | 80 | 19.59 | 17 | 22.47 | 0.5689 | 99.2 |
|  | High-quality studies only | 60 | 20.92 | 17.34 | 25.01 | 0.7781 | 98.9 |
| *Streptococcus* spp. | All studies | 60 | 17.29 | 15.14 | 19.66 | 0.3397 | 99.2 |
|  | High-quality studies only | 43 | 18.53 | 16.22 | 21.1 | 0.2492 | 98.1 |
| *E. coli* | All studies | 52 | 13.86 | 11.22 | 17 | 0.7203 | 98.9 |
|  | High-quality studies only | 37 | 12.67 | 10.54 | 15.17 | 0.3612 | 97.7 |
| CNS | All studies | 32 | 15.49 | 10.12 | 22.99 | 1.9408 | 99.6 |
|  | High-quality studies only | 26 | 16.31 | 11.55 | 22.52 | 1.0505 | 98.9 |
| *Klebsiella* spp. | All studies | 28 | 9.92 | 7.38 | 13.2 | 0.6616 | 98.1 |
|  | High-quality studies only | 19 | 10.8 | 7.71 | 14.92 | 0.6186 | 98.5 |

**Table S13.** **Research information and epidemiological situation of *Enterococcus* spp. causing bovine mastitis.**

| **Accession number** | **Published year** | **Year of samples collection** | **Country** | **Continent** | **Climate** | **Intensive or not** | **Purebred or not** | **Sample size**  **(*n*)** | **Number of positive**  **(*n*)** | **Positive rate**  **,%** | **CI lower,%** | **CI upper,%** |
| --- | --- | --- | --- | --- | --- | --- | --- | --- | --- | --- | --- | --- |
| 27801370 | 2016 | 2013-2014 | Kosovo | Europe | Temperate | NA | NA | 152 | 22 | 14.47 | 9.76 | 20.94 |
| 24119795 | 2013 | NA | United States | North America | Temperate | Yes | NA | 741 | 15 | 2.02 | 1.23 | 3.31 |
| 39595342 | 2024 | NA | China | Asia | Temperate | NA | Yes | 168 | 15 | 8.93 | 5.49 | 14.21 |
| 39180074 | 2024 | 2017-2019 | Brazil | South America | Plateau | NA | Yes | 1577 | 93 | 5.90 | 4.84 | 7.17 |
| 18349229 | 2018 | 2003-2005 | Canada | North America | Temperate | NA | Yes | 3149 | 68 | 2.16 | 1.71 | 2.73 |
| 26686728 | 2016 | 2011-2012 | Canada | North America | Temperate | NA | NA | 936 | 33 | 3.53 | 2.52 | 4.91 |
| WOS:001098206200001 | 2023 | 2022 | China | Asia | Subtropical | Yes | NA | 208 | 20 | 9.62 | 6.31 | 14.38 |
| 39077446 | 2024 | 2023 | Thailand | Asia | Tropical | NA | No | 84 | 1 | 1.19 | 0.21 | 6.44 |
| 30989140 | 2019 | 2014-2017 | Poland | Europe | Temperate | NA | NA | 2000 | 426 | 21.30 | 19.56 | 23.15 |
| WOS:000319654700139 | 2013 | 2010-2011 | Uganda | Africa | Tropical | NA | NA | 97 | 16 | 16.49 | 10.42 | 25.13 |
| WOS:001234881800001 | 2024 | 2018-2023 | China | Asia | Temperate | Yes | NA | 2897 | 198 | 6.83 | 5.97 | 7.81 |
| WOS:000390631000004 | 2016 | 2011-2012 | China | Asia | NA | NA | NA | 280 | 60 | 21.43 | 17.03 | 26.60 |
| WOS:000416127200002 | 2017 | NA | Australia | Oceania | Subtropical | Yes | NA | 288 | 5 | 1.74 | 0.74 | 4 |
| 40266927 | 2025 | 2015-2024 | China | Asia | Temperate | Yes | NA | 12053 | 451 | 3.74 | 3.42 | 4.10 |
| Random effects model (6.3657 [3.8034; 10.4666], I^2^=98.7%, *P*< 0.0001，X^2^= 996.66) | | | | | | | | | | | | |

**Table S14.** **Research information and epidemiological situation of *Pseudomonas* spp. causing bovine mastitis.**

| **Accession number** | **Published year** | **Year of samples collection** | **Country** | **Continent** | **Climate** | **Intensive or not** | **Purebred or not** | **Sample size**  **(*n*)** | **Number of positive**  **(*n*)** | **Positive rate**  **,%** | **CI lower,%** | **CI upper,%** |
| --- | --- | --- | --- | --- | --- | --- | --- | --- | --- | --- | --- | --- |
| 34511943 | 2021 | 2016-2019 | China | Asia | Temperate | Yes | No | 830 | 32 | 3.86 | 2.74 | 5.39 |
| 39595342 | 2024 | NA | China | Asia | Temperate | NA | Yes | 168 | 63 | 37.50 | 30.54 | 45.02 |
| 36009946 | 2022 | 2021 | Serbia | Europe | Temperate | Yes | No | 81 | 3 | 3.70 | 1.27 | 10.33 |
| 29757079 | 2018 | 2015-2017 | Tunisia | Africa | Subtropical | No | NA | 452 | 5 | 1.11 | 0.47 | 2.56 |
| 18349229 | 2018 | 2003-2005 | Canada | North America | Temperate | NA | Yes | 3149 | 23 | 0.73 | 0.49 | 1.09 |
| 26686728 | 2016 | 2011-2012 | Canada | North America | Temperate | NA | NA | 936 | 12 | 1.28 | 0.73 | 2.23 |
| WOS:001098206200001 | 2023 | 2022 | China | Asia | Subtropical | Yes | NA | 208 | 54 | 25.96 | 20.48 | 32.32 |
| WOS:000919499400001 | 2023 | 2021 | China | Asia | Subtropical | Yes | NA | 156 | 2 | 1.28 | 0.35 | 4.55 |
| 40266927 | 2025 | 2015-2024 | China | Asia | Temperate | Yes | NA | 12053 | 298 | 2.47 | 2.21 | 2.77 |
| https://doi.org/10.22207/JPAM.11.1.41 | 2017 | NA | India | Asia | Tropical | NA | NA | 50 | 9 | 18 | 9.77 | 30.8 |
| Random effects model (4.2939 [1.6121; 10.9406%], I^2^=98.6%, *P*< 0.0001，X^2^= 649.63 ) | | | | | | | | | | | | |

**Table S15.** **Research information and epidemiological situation of *Bacillus* spp. causing bovine mastitis.**

| **Accession number** | **Published year** | **Year of samples collection** | **Country** | **Continent** | **Climate** | **Intensive or not** | **Purebred or not** | **Sample size**  **(*n*)** | **Number of positive**  **(*n*)** | **Positive rate**  **,%** | **CI lower,%** | **CI upper,%** |
| --- | --- | --- | --- | --- | --- | --- | --- | --- | --- | --- | --- | --- |
| 28434736 | 2017 | 2014-2016 | China | Asia | NA | Yes | No | 3288 | 42 | 1.28 | 0.95 | 1.72 |
| 18349229 | 2018 | 2003-2005 | Canada | North America | Temperate | NA | Yes | 3149 | 32 | 1.02 | 0.72 | 1.43 |
| 26686728 | 2016 | 2011-2012 | Canada | North America | Temperate | NA | NA | 936 | 152 | 16.24 | 14.02 | 18.74 |
| 34438713 | 2021 | 2018 | Bangladesh | Asia | Subtropical | NA | NA | 153 | 29 | 18.95 | 13.53 | 25.90 |
| WOS:001098206200001 | 2023 | 2022 | China | Asia | Subtropical | Yes | NA | 208 | 29 | 13.94 | 9.89 | 19.31 |
| WOS:000270226200065 | 2009 | 2007-2008 | Ethiopia | Africa | Plateau | No | NA | 144 | 12 | 8.33 | 4.83 | 14 |
| WOS:000558156400004 | 2020 | 2018 | China | Asia | NA | NA | NA | 1153 | 95 | 8.24 | 6.79 | 9.97 |
| 40266927 | 2025 | 2015-2024 | China | Asia | Temperate | Yes | NA | 12053 | 157 | 1.30 | 1.12 | 1.52 |
| 39595342 | 2024 | NA | China | Asia | Temperate | NA | Yes | 168 | 61 | 36.31 | 29.42 | 43.81 |
| 29757079 | 2018 | 2015-2017 | Tunisia | Africa | Temperate | No | NA | 452 | 5 | 1.11 | 0.47 | 2.56 |
| Random effects model (5.7682 [2.3335; 13.5568]%, I^2^=99.1%, *P*< 0.0001，X^2^=1020.3) | | | | | | | | | | | | |

**Table S16.** **Research information and epidemiological situation of *Yeast* causing bovine mastitis.**

| **Accession number** | **Published year** | **Year of samples collection** | **Country** | **Continent** | **Climate** | **Intensive or not** | **Purebred or not** | **Sample size**  **(*n*)** | **Number of positive**  **(*n*)** | **Positive rate**  **,%** | **CI lower,%** | **CI upper,%** |
| --- | --- | --- | --- | --- | --- | --- | --- | --- | --- | --- | --- | --- |
| 27801370 | 2016 | 2013-2014 | Kosovo | Europe | Temperate | NA | NA | 152 | 8 | 5.26 | 2.69 | 10.04 |
| 24119795 | 2013 | NA | United States | North America | Temperate | Yes | NA | 741 | 21 | 2.83 | 1.86 | 4.29 |
| 30157847 | 2018 | NA | China | Asia | Temperate | NA | NA | 482 | 256 | 53.11 | 48.65 | 57.52 |
| 28434736 | 2017 | 2014-2016 | China | Asia | NA | Yes | No | 3288 | 32 | 0.97 | 0.69 | 1.37 |
| 18349229 | 2018 | 2003-2005 | Canada | North America | NA | NA | Yes | 3149 | 57 | 1.81 | 1.40 | 2.34 |
| 26686728 | 2016 | 2011-2012 | Canada | North America | NA | NA | NA | 936 | 14 | 1.50 | 0.89 | 2.49 |
| WOS:001480412600001 | 2025 | 2022 | Egypt | Africa | NA | NA | No | 130 | 3 | 2.31 | 0.79 | 6.57 |
| 34955276 | 2022 | 2011-2012 | Australia | Oceania | NA | NA | NA | 3219 | 6 | 0.19 | 0.09 | 0.41 |
| 40266927 | 2025 | 2015-2024 | China | Asia | Temperate | Yes | NA | 12053 | 221 | 1.83 | 1.61 | 2.09 |
| WOS:000351168800022 | 2015 | 2010-2012 | Algeria | Africa | Temperate | NA | NA | 865 | 66 | 7.63 | 6.04 | 9.59 |
| WOS:000735317700001 | 2021 | 2018-2019 | Poland | Europe | Temperate | NA | No | 48 | 3 | 6.25 | 2.15 | 16.84 |
| 29397171 | 2018 | 2007-2008 | Canada | North America | NA | NA | NA | 682 | 6 | 0.88 | 0.40 | 1.91 |
| Random effects model (2.7132 [0.8879; 7.9879]%, I^2^=99.3%, *P*< 0.0001，X^2^=1642.75) | | | | | | | | | | | | |

**Table S17.** **Research information and epidemiological situation of *Corynebacterium* spp. causing bovine mastitis.**

| **Accession number** | **Published year** | **Year of samples collection** | **Country** | **Continent** | **Climate** | **Intensive or not** | **Purebred or not** | **Sample size**  **(*n*)** | **Number of positive**  **(*n*)** | **Positive rate**  **,%** | **CI lower,%** | **CI upper,%** |
| --- | --- | --- | --- | --- | --- | --- | --- | --- | --- | --- | --- | --- |
| WOS:000391351600042 | 2017 | 2007-2008 | Finland | Europe | Temperate | Yes | Yes | 29969 | 1368 | 4.56 | 4.33 | 4.81 |
| 28434736 | 2017 | 2014-2016 | China | Asia | NA | Yes | No | 3288 | 20 | 0.61 | 0.39 | 0.94 |
| 18349229 | 2018 | 2003-2005 | Canada | North America | Temperate | NA | Yes | 3149 | 6 | 0.19 | 0.09 | 0.42 |
| 26686728 | 2016 | 2011-2012 | Canada | North America | Temperate | NA | NA | 936 | 32 | 3.42 | 2.43 | 4.79 |
| WOS:000270226200065 | 2009 | 2007-2008 | Ethiopia | Africa | Plateau | No | NA | 144 | 5 | 3.47 | 1.49 | 7.87 |
| 19447987 | 2009 | 2004-2005 | United Kingdom | Europe | Temperate | NA | No | 929 | 15 | 1.61 | 0.98 | 2.65 |
| 34955276 | 2022 | 2011-2012 | Australia | Oceania | NA | NA | NA | 3219 | 82 | 2.55 | 2.06 | 3.15 |
| 40266927 | 2025 | 2015-2024 | China | Asia | Temperate | Yes | NA | 12053 | 289 | 2.40 | 2.14 | 2.69 |
| Random effects model (1.8070 [1.1663; 2.7898]%, I^2^=97.3%, *P*< 0.0001，X^2^= 260.87 ) | | | | | | | | | | | | |

**Table S18.** **Research information and epidemiological situation of *Enterobacter* spp. causing bovine mastitis.**

| **Accession number** | **Published year** | **Year of samples collection** | **Country** | **Continent** | **Climate** | **Intensive or not** | **Purebred or not** | **Sample size**  **(*n*)** | **Number of positive**  **(*n*)** | **Positive rate**  **,%** | **CI lower,%** | **CI upper,%** |
| --- | --- | --- | --- | --- | --- | --- | --- | --- | --- | --- | --- | --- |
| 34511943 | 2021 | 2016-2019 | China | Asia | Temperate | Yes | No | 830 | 53 | 6.39 | 4.91 | 8.26 |
| 30790157 | 2019 | 2007-2016 | India | Asia | Subtropical | Yes | NA | 3945 | 43 | 1.09 | 0.81 | 1.46 |
| 24119795 | 2013 | NA | United States | North America | Temperate | Yes | NA | 741 | 27 | 3.64 | 2.52 | 5.25 |
| 29757079 | 2018 | 2015-2017 | Tunisia | Africa | Subtropical | No | NA | 452 | 19 | 4.20 | 2.71 | 6.47 |
| 28434736 | 2017 | 2014-2016 | China | Asia | NA | Yes | No | 3288 | 182 | 5.54 | 4.80 | 6.37 |
| 26686728 | 2016 | 2011-2012 | Canada | North America | Temperate | NA | NA | 936 | 28 | 2.99 | 2.08 | 4.29 |
| WOS:000509758800099 | 2019 | 2017 | Bosnia and Herzegovina | Europe | Temperate | No | NA | 120 | 1 | 0.83 | 0.15 | 4.57 |
| WOS:000987293900001 | 2023 | 2018-2022 | China | Asia | Temperate | NA | NA | 2897 | 32 | 1.10 | 0.78 | 1.56 |
| 26255183 | 2015 | NA | Mexico | North America | Tropical | No | NA | 53 | 5 | 9.43 | 4.10 | 20.25 |
| Random effects model (3.1329 [1.8491; 5.2603]%, I^2^=95.3%, *P*< 0.0001，X^2^= 169.88 ) | | | | | | | | | | | | |

**Table S19.** **Research information and epidemiological situation of *Mycoplasma* spp. causing bovine mastitis.**

| **Accession number** | **Published year** | **Year of samples collection** | **Country** | **Continent** | **Climate** | **Intensive or not** | **Purebred or not** | **Sample size**  **(*n*)** | **Number of positive**  **(*n*)** | **Positive rate**  **,%** | **CI lower,%** | **CI upper,%** |
| --- | --- | --- | --- | --- | --- | --- | --- | --- | --- | --- | --- | --- |
| WOS:000432327500014 | 2018 | 2015-2016 | Iran | Asia | Temperate | Yes | NA | 328 | 97 | 29.57 | 24.89 | 34.73 |
| 32810702 | 2020 | 2018-2019 | China | Asia | Temperate | Yes | No | 450 | 39 | 8.67 | 6.40 | 11.63 |
| WOS:001535763000001 | 2025 | 2009-2023 | United States | North America | Temperate | NA | NA | 56650 | 1029 | 1.82 | 1.71 | 1.93 |
| WOS:000416127200002 | 2017 | NA | Australia | Oceania | Temperate | Yes | NA | 288 | 7 | 2.43 | 1.18 | 4.93 |
| 40266927 | 2025 | 2015-2024 | China | Asia | Temperate | Yes | NA | 12053 | 322 | 2.67 | 2.40 | 2.97 |
| 28601455 | 2017 | 2014 | Estonia | Europe | NA | Yes | NA | 522 | 90 | 17.24 | 14.24 | 20.72 |
| Random effects model (6.4950 [2.6446; 15.0827]%, I^2^=99.5%, *P*< 0.0001，X^2^= 1026.19) | | | | | | | | | | | | |

**Table S20.** **Research information and epidemiological situation of** ***Trueperella* spp. causing bovine mastitis.**

| **Accession number** | **Published year** | **Year of samples collection** | **Country** | **Continent** | **Climate** | **Intensive or not** | **Purebred or not** | **Sample size**  **(*n*)** | **Number of positive**  **(*n*)** | **Positive rate**  **,%** | **CI lower,%** | **CI upper,%** |
| --- | --- | --- | --- | --- | --- | --- | --- | --- | --- | --- | --- | --- |
| 33153084 | 2020 | 2014-2018 | Germany | Europe | Temperate | Yes | NA | 2883 | 38 | 1.32 | 0.96 | 1.80 |
| 29757079 | 2018 | 2015-2017 | Tunisia | Africa | Subtropical | No | NA | 452 | 4 | 0.88 | 0.34 | 2.25 |
| 28434736 | 2017 | 2014-2016 | China | Asia | NA | Yes | No | 3288 | 30 | 0.91 | 0.64 | 1.30 |
| 26686728 | 2016 | 2011-2012 | Canada | North America | Temperate | NA | NA | 936 | 19 | 2.03 | 1.30 | 3.15 |
| 29397171 | 2018 | 2007-2008 | Canada | North America | NA | NA | NA | 682 | 24 | 3.52 | 2.38 | 5.18 |
| Random effects model (1.5607 [0.9302; 2.6074]%, I^2^=86%, *P*< 0.0001，X^2^= 28.59 ) | | | | | | | | | | | | |

**Table S21.** **Research information and epidemiological situation of** ***Proteus* spp. causing bovine mastitis.**

| **Accession number** | **Published year** | **Year of samples collection** | **Country** | **Continent** | **Climate** | **Intensive or not** | **Purebred or not** | **Sample size**  **(*n*)** | **Number of positive**  **(*n*)** | **Positive rate**  **,%** | **CI lower,%** | **CI upper,%** |
| --- | --- | --- | --- | --- | --- | --- | --- | --- | --- | --- | --- | --- |
| 36964326 | 2023 | 2022 | Brazil | South America | Tropical | NA | NA | 257 | 4 | 1.56 | 0.61 | 3.93 |
| 26255183 | 2015 | NA | Mexico | North America | Tropical | No | NA | 53 | 15 | 28.30 | 17.97 | 41.57 |
| 40266927 | 2025 | 2015-2024 | China | Asia | Temperate | Yes | NA | 12053 | 289 | 2.40 | 2.14 | 2.69 |
| Random effects model (5.1445 [0.7798; 27.2343]%, I^2^=97.5%, *P*< 0.0001，X^2^= 81.03 ) | | | | | | | | | | | | |

**Table S22.** **Research information and epidemiological situation of** ***Lactococcus* spp. causing bovine mastitis.**

| **Accession number** | **Published year** | **Year of samples collection** | **Country** | **Continent** | **Climate** | **Intensive or not** | **Purebred or not** | **Sample size**  **(*n*)** | **Number of positive**  **(*n*)** | **Positive rate**  **,%** | **CI lower,%** | **CI upper,%** |
| --- | --- | --- | --- | --- | --- | --- | --- | --- | --- | --- | --- | --- |
| WOS:000940708300001 | 2023 | 2020-2021 | China | Asia | NA | NA | NA | 1441 | 49 | 3.40 | 2.58 | 4.47 |
| WOS:000982776200001 | 2023 | 2017-2021 | China | Asia | Temperate | Yes | NA | 2899 | 39 | 1.35 | 0.99 | 1.83 |
| 40266927 | 2025 | 2015-2024 | China | Asia | Temperate | Yes | NA | 12053 | 386 | 3.20 | 2.90 | 3.53 |
| Random effects model (2.4853 [1.5240; 4.0280]%, I^2^=92.9%, *P*< 0.0001，X^2^= 28.29 ) | | | | | | | | | | | | |

**Fig. S1.** **Funnel plot for the meta-analysis of bovine mastitis caused by *S. aureus*.**

**Fig. S2.** **Funnel plot for the meta-analysis of bovine mastitis caused by *Streptococcus* spp.**

**Fig. S3.** **Funnel plot for the meta-analysis of bovine mastitis caused by** ***E.coli*.**

**Fig. S4.** **Funnel plot for the meta-analysis of bovine mastitis caused by** **CNS*.***

**Fig. S5.** **Funnel plot for the meta-analysis of bovine mastitis caused by** ***Klebsiella* spp*.***

**Fig. S6. Period-specific hotspot countries for *S. aureus* infection in dairy cow mastitis.**

**Fig. S7. Period-specific hotspot countries for *Streptococcus* spp. infection in dairy cow mastitis.**

**Fig. S8. Period-specific hotspot countries for *E. coli* infection in dairy cow mastitis.**

**Fig. S9. Period-specific hotspot countries for CNS infection in dairy cow mastitis.**

**Fig. S10. Period-specific hotspot countries for *Klebsiella* spp. infection in dairy cow mastitis.**

**Fig. S11. Prevalence of *S. aureus* in dairy cow mastitis: a meta-analysis and subgroup analysis by country.**

**Fig. S12. Prevalence of *S. aureus* in dairy cow mastitis: a meta-analysis and subgroup analysis by continent.**

 **Fig. S13. Prevalence of *S. aureus* in dairy cow mastitis: a meta-analysis and subgroup analysis by climate.**

**Fig. S14. Prevalence of *S. aureus* in dairy cow mastitis: a meta-analysis and subgroup analysis by breed purity.**

**Fig. S15. Prevalence of *S. aureus* in dairy cow mastitis: a meta-analysis and subgroup analysis by** **intensification.**

**Fig. S16. Prevalence of *Streptococcus* spp. in dairy cow mastitis: a meta-analysis and subgroup analysis by country.**

**Fig. S17. Prevalence of *Streptococcus* spp. in dairy cow mastitis: a meta-analysis and subgroup analysis by continent.**

**Fig. S18. Prevalence of *Streptococcus* spp. in dairy cow mastitis: a meta-analysis and subgroup analysis by climate.**

**Fig. S19. Prevalence of *Streptococcus* spp. in dairy cow mastitis: a meta-analysis and subgroup analysis by breed purity.**

**Fig. S20. Prevalence of *Streptococcus* spp. in dairy cow mastitis: a meta-analysis and subgroup analysis by** **intensification.**

**Fig. S21. Prevalence of *E. coli* in dairy cow mastitis: a meta-analysis and subgroup analysis by country.**

**Fig. S22. Prevalence of *E. coli* in dairy cow mastitis: a meta-analysis and subgroup analysis by continent.**

**Fig. S23. Prevalence of *E. coli* in dairy cow mastitis: a meta-analysis and subgroup analysis by climate.**

**Fig. S24. Prevalence of *E. coli* in dairy cow mastitis: a meta-analysis and subgroup analysis by breed purity.**

**Fig. S25. Prevalence of *E. coli* in dairy cow mastitis: a meta-analysis and subgroup analysis by** **intensification.**

**Fig. S26. Prevalence of CNS in dairy cow mastitis: a meta-analysis and subgroup analysis by country.**

**Fig. S27. Prevalence of CNS in dairy cow mastitis: a meta-analysis and subgroup analysis by continent.**

**Fig. S28. Prevalence of CNS in dairy cow mastitis: a meta-analysis and subgroup analysis by climate.**

**Fig. S29. Prevalence of CNS in dairy cow mastitis: a meta-analysis and subgroup analysis by breed purity.**

**Fig. S30. Prevalence of CNS in dairy cow mastitis: a meta-analysis and subgroup analysis by** **intensification.**

**Fig. S31. Prevalence of *Klebsiella* spp. in dairy cow mastitis: a meta-analysis and subgroup analysis by country.**

**Fig. S32. Prevalence of *Klebsiella* spp. in dairy cow mastitis: a meta-analysis and subgroup analysis by continent.**

**Fig. S33. Prevalence of *Klebsiella* spp. in dairy cow mastitis: a meta-analysis and subgroup analysis by climate.**

**Fig. S34. Prevalence of *Klebsiella* spp. in dairy cow mastitis: a meta-analysis and subgroup analysis by breed purity.**

**Fig. S35. Prevalence of *Klebsiella* spp. in dairy cow mastitis: a meta-analysis and subgroup analysis by** **intensification.**

**Fig. S36. Spatiotemporal distribution of *Enterococcus* spp. causing dairy cow mastitis (2000-2025).**

A. Country-level ranked prevalence plots. B. Time trend in the prevalence.

**Fig. S37. Spatiotemporal distribution of *Pseudomonas* spp. causing dairy cow mastitis (2000-2025).**

A. Country-level ranked prevalence plots. B. Time trend in the prevalence.

**Fig. S38. Spatiotemporal distribution of *Yeast* causing dairy cow mastitis (2000-2025).**

A. Country-level ranked prevalence plots. B. Time trend in the prevalence.

**Fig. S39. Spatiotemporal distribution of *Bacillus* spp. causing dairy cow mastitis (2000-2025).**

A. Country-level ranked prevalence plots. B. Time trend in the prevalence.

**Fig. S40. Spatiotemporal distribution of *Corynebacterium* spp. causing dairy cow mastitis (2000-2025).**

A. Country-level ranked prevalence plots. B. Time trend in the prevalence.

**Fig. S41. Spatiotemporal distribution of *Mycoplasma* spp. causing dairy cow mastitis (2000-2025).**

A. Country-level ranked prevalence plots. B. Time trend in the prevalence.

**Fig. S42. Spatiotemporal distribution of *Trueperella* spp. causing dairy cow mastitis (2000-2025).**

A. Country-level ranked prevalence plots. B. Time trend in the prevalence.

**Fig. S43. Spatiotemporal distribution of *Enterobacter* spp. causing dairy cow mastitis (2000-2025).**

A. Country-level ranked prevalence plots. B. Time trend in the prevalence.

**Fig. S44. Spatiotemporal distribution of *Proteus* spp. causing dairy cow mastitis (2000-2025).**

A. Country-level ranked prevalence plots. B. Time trend in the prevalence.

**Fig. S45. Spatiotemporal distribution of *Lactococcus* spp. causing dairy cow mastitis (2000-2025).**

A. Country-level ranked prevalence plots. B. Time trend in the prevalence.

**References：**

1.	Brahma U, Suresh A, Murthy S, Bhandari V, Sharma P. Antibiotic resistance and molecular profiling of the clinical isolates of Staphylococcus aureus causing bovine mastitis from India. Microorganisms. 2022;10(4).

2.	Li K, Hou MY, Zhang L, Tian MY, Yang M, Jia L, et al. Analysis of antimicrobial resistance and genetic correlations of Escherichia coli in dairy cow mastitis. J Vet Res. 2022;66(4):571-9.

3.	Kumar R, Yadav BR, Singh RS. Antibiotic resistance and pathogenicity factors in Staphylococcus aureus isolated from mastitic Sahiwal cattle. J Biosci. 2011;36(1):175-88.

4.	Zaatout N, Ayachi A, Kecha M, Kadlec K. Identification of staphylococci causing mastitis in dairy cattle from Algeria and characterization of Staphylococcus aureus. J Appl Microbiol. 2019;127(5):1305-14.

5.	My TT, Thien LV, Manh VD, My BTP, Lan DTM, Binh DX, et al. Antimicrobial resistance and molecular characterization of Escherichia coli isolated from bovine mastitis samples in Nghe An province, Vietnam. Vet World. 2023;16(4):743-51.

6.	Tran MT, Vu DM, Vu MD, Bui MTP, Dang BX, Dang LTM, et al. Antimicrobial resistance and molecular characterization of Klebsiella species causing bovine mastitis in Nghe An province, Vietnam. J Adv Vet Anim Res. 2023;10(1):132-43.

7.	Wang J, Li J, Ji X, Zhang L, Wang R, Wang H, et al. Antimicrobial resistance and molecular epidemiology of Klebsiella pneumoniae isolated from bovine mastitis in seven provinces in China. BMC Microbiol. 2025;25(1).

8.	Shen JR, Wu XH, Yang YY, Lv YN, Li XP, Ding XZ, et al. Antimicrobial resistance and virulence factor of Streptococcus dysgalactiae isolated from clinical bovine mastitis cases in northwest China. Infect Drug Resist. 2021;14:3519-30.

9.	Guo Y, Qiu P, Su H, Li Y, Guo Y, Zhang Z, et al. Antimicrobial resistance and virulence genes distribution in Trueperella pyogenes isolated from dairy cows with clinical mastitis in Liaoning of China. Pak Vet J. 2021;41(3):329-34.

10.	Tian XY, Zheng N, Han RW, Ho H, Wang J, Wang YT, et al. Antimicrobial resistance and virulence genes of Streptococcus isolated from dairy cows with mastitis in China. Microb Pathog. 2019;131:33-9.

11.	Ding YX, Zhao JL, He XL, Li M, Guan H, Zhang ZY, et al. Antimicrobial resistance and virulence-related genes of Streptococcus obtained from dairy cows with mastitis in Inner Mongolia, China. Pharm Biol. 2016;54(1):162-7.

12.	Mehmeti I, Behluli B, Mestani M, Ademi A, Nes IF, Diep DB. Antimicrobial resistance levels amongst staphylococci isolated from clinical cases of bovine mastitis in Kosovo. J Infect Dev Ctries. 2016;10(10):1081-7.

13.	Wang D, Zhang LM, Zhou XZ, He YL, Yong CF, Shen ML, et al. Antimicrobial susceptibility, virulence genes, and randomly amplified polymorphic DNA analysis of Staphylococcus aureus recovered from bovine mastitis in Ningxia, China. J Dairy Sci. 2016;99(12):9560-9.

14.	Al-harbi H, Ranjbar S, Moore RJ, Alawneh JI. Bacteria isolated from milk of dairy cows with and without clinical mastitis in different regions of Australia and their AMR profiles. Front Vet Sci. 2021;8.

15.	Bhutia PS, Bansal BK, Gupta DK, Singh RS, Uppal SK. Bacterial isolation of milk samples submitted from clinical mastitis buffaloes during 2007 to 2016. Trop Anim Health Prod. 2019;51(6):1551-7.

16.	Ebrahimi A, Moatamedi A, Lotfalian S, Mirshokraei P. Biofilm formation, hemolysin production and antimicrobial susceptibilities of Streptococcus agalactiae isolated from the mastitis milk of dairy cows in Shahrekord district, Iran. Vet Res Forum. 2013;4(4):269-72.

17.	Oliveira L, Hulland C, Ruegg PL. Characterization of clinical mastitis occurring in cows on 50 large dairy herds in Wisconsin. J Dairy Sci. 2013;96(12):7538-49.

18.	Huang YC, Chen PQ, Cao HA, Zhou Z, Xu TL. Characterization of Pseudomonas aeruginosa isolated from bovine mastitis in northern Jiangsu province and correlation to drug resistance and biofilm formability. Animals. 2024;14(22).

19.	Schmenger A, Krömker V. Characterization, cure rates and associated risks of clinical mastitis in northern Germany. Vet Sci. 2020;7(4):1-17.

20.	Abdalhamed AM, Ibrahim ES, El-Shafey DYH, Zeedan GSG. Detecting Mycoplasma bovis by SYBR green-based real-time quantitative PCR and loop-mediated isothermal amplification methods. Pak Vet J. 2024;44(3):847-53.

21.	Zhang Z, Chen Y, Li XP, Wang XR, Li HS. Detection of antibiotic resistance, virulence gene, and drug resistance gene of Staphylococcus aureus isolates from bovine mastitis. Microbiol Spectr. 2022;10(4).

22.	Xu J, Tan X, Zhang XY, Xia XL, Sun HC. The diversities of staphylococcal species, virulence and antibiotic resistance genes in the subclinical mastitis milk from a single Chinese cow herd. Microb Pathog. 2015;88:29-38.

23.	Hassan J, Bag MAS, Ali MW, Kabir A, Hoque MN, Hossain MM, et al. Diversity of Streptococcus spp. and genomic characteristics of Streptococcus uberis isolated from clinical mastitis of cattle in Bangladesh. Front Vet Sci. 2023;10.

24.	Tomanic D, Bozin B, Kladar N, Stanojevic J, Cabarkapa I, Stilinovic N, et al. Environmental bovine mastitis pathogens: prevalence, antimicrobial susceptibility, and sensitivity to Thymus vulgaris L., Thymus serpyllum L., and Origanum vulgare L. Essential oils. Antibiotics. 2022;11(8).

25.	Abd El-Aziz NK, Ammar AM, El Damaty HM, Abd Elkader RA, Saad HA, El-Kazzaz W, et al. Environmental Streptococcus uberis associated with clinical mastitis in dairy cows: Virulence traits, antimicrobial and biocide resistance, and epidemiological typing. Animals. 2021;11(7).

26.	Du J, Wang X, Luo H, Wang Y, Liu X, Zhou X. Epidemiological investigation of non-albicans Candida species recovered from mycotic mastitis of cows in Yinchuan, Ningxia of China. BMC Vet Res. 2018;14(1).

27.	Klibi A, Jouini A, Boubaker El Andolsi R, Kmiha S, Ben Hamda C, Ghedira K, et al. Epidemiology of β-lactamase-producing Staphylococci and gram negative bacteria as cause of clinical bovine mastitis in Tunisia. Biomed Res Int. 2019;2019.

28.	Saidani M, Messadi L, Soudani A, Daaloul-Jedidi M, Châtre P, Ben Chehida F, et al. Epidemiology, antimicrobial resistance, and extended-spectrum beta-lactamase-producing Enterobacteriaceae in clinical bovine mastitis in Tunisia. Microb Drug Resist. 2018;24(8):1242-8.

29.	Salat O, Lemaire G, Durel L, Perrot F. Etiology of severe mastitis in French dairy herds. PLoS One. 2023;18(12 December).

30.	Taponen S, Liski E, Heikkilä AM, Pyörälä S. Factors associated with intramammary infection in dairy cows caused by coagulase-negative staphylococci, Staphylococcus aureus, Streptococcus uberis, Streptococcus dysgalactiae, Corynebacterium bovis, or Escherichia coil. J Dairy Sci. 2017;100(1):493-503.

31.	Fredebeul-Krein F, Schmenger A, Wente N, Zhang Y, Krömker V. Factors associated with the severity of clinical mastitis. Pathogens. 2022;11(10).

32.	Karabasanavar NS, Radder SK, Sivaraman GK. Field level interventions on subclinical mastitis and detection of Staphylococcus in crossbred dairy cows. Indian J Anim Sci. 2019;89(7):7-13.

33.	Mohsenzadeh M, Ghazvini K, Azimian A. Frequency of specific agr groups and antibiotic resistance in Staphylococcus aureus isolated from bovine mastitis in the northeast of Iran. Vet Res Forum. 2015;6(4):295-9.

34.	Katira BP, Prajapati BI, Singh RD, Patel SS, Solanki KM. Genetic characterization of virulence and extended spectrum β-lactamase producing genes of Klebsiella pneumoniae isolated from bovine milk. Vet Res Forum. 2024;15(2):57-64.

35.	Cheng J, Zhou M, Nobrega DB, Barkema HW, Xu S, Li M, et al. Genetic diversity and molecular epidemiology of outbreaks of Klebsiella pneumoniae mastitis on two large Chinese dairy farms. J Dairy Sci. 2021;104(1):762-75.

36.	Wang L, Chen W, Zhang L, Zhu Y. Genetic diversity of Streptococcus uberis isolates from dairy cows with subclinical mastitis in southern Xinjiang province, China. J Gen Appl Microbiol. 2013;59(4):287-93.

37.	Panigrahi S, Biswal S, Padhi SK, Pahari A, Bhuyan K, Sahoo N. Genotypic and phenotypic characterization of Mammaliicoccus sciuri - AMDR strain causing clinical mastitis in cows of Odisha, India. Indian J Anim Res. 2022;56(12):1537-41.

38.	Huilca-Ibarra MP, Vasco-Julio D, Ledesma Y, Guerrero-Freire S, Zurita J, Castillejo P, et al. High prevalence of Prototheca bovis infection in dairy cattle with chronic mastitis in Ecuador. Vet Sci. 2022;9(12).

39.	Guimarães FF, Moraes GN, Joaquim SF, Guerra ST, Dalanezi FM, Mioni MSR, et al. Identification of Enterococcus spp. by MALDI-TOF mass spectrometry isolated from clinical mastitis and bulk tank milk samples. BMC Vet Res. 2024;20(1).

40.	Mahato S, Mistry HU, Chakraborty S, Sharma P, Saravanan R, Bhandari V. Identification of variable traits among the methicillin resistant and sensitive coagulase negative staphylococci in milk samples from mastitic cows in India. Front Microbiol. 2017;8(JUL).

41.	Gao J, Barkema HW, Zhang L, Liu G, Deng Z, Cai L, et al. Incidence of clinical mastitis and distribution of pathogens on large Chinese dairy farms. J Dairy Sci. 2017;100(6):4797-806.

42.	Olde Riekerink RG, Barkema HW, Kelton DF, Scholl DT. Incidence rate of clinical mastitis on Canadian dairy farms. J Dairy Sci. 2008;91(4):1366-77.

43.	Levison LJ, Miller-Cushon EK, Tucker AL, Bergeron R, Leslie KE, Barkema HW, et al. Incidence rate of pathogen-specific clinical mastitis on conventional and organic Canadian dairy farms. J Dairy Sci. 2016;99(2):1341-50.

44.	Singha S, Koop G, Persson Y, Hossain D, Scanlon L, Derks M, et al. Incidence, etiology, and risk factors of clinical mastitis in dairy cows under semi-tropical circumstances in Chattogram, Bangladesh. Animals. 2021;11(8).

45.	Adhikari S, Khanal S, Marasini A, Panthi P, Adhikari A. Investigation of antimicrobial resistance and biofilm-forming ability in Staphylococcus aureus causing clinical bovine mastitis in Chitwan, Nepal. Vet Anim Sci. 2025;30:100508.

46.	Feyissa N, Alemu T, Birri DJ, Desalegn A, Sombo M, Abera S. Isolation and determination of antibacterial sensitivity characteristics of Staphylococcus aureus from lactating cows in west Shewa Zone, Ethiopia. Vet Med Int. 2023;2023.

47.	Cao WD, Xu Y, Huang YC, Xu TL. Isolation of pathogenic bacteria from dairy cow mastitis and correlation of biofilm formation and drug resistance of Klebsiella pneumoniae in Jiangsu, China. Agriculture-Basel. 2023;13(10).

48.	Yimana M, Tesfaye J. Isolation, identification and antimicrobial profile of methicillin-resistant Staphylococcus aureus from bovine mastitis in and around Adama, Central Ethiopia. Vet Med Sci. 2022;8(6):2576-84.

49.	Imandar M, Pourbakhsh SA, Jamshidian M, Zahraei ST. Isolation, identification and molecular characterization of Mycoplasma bovis in mastitic dairy cattle by PCR and culture methods. J Hell Vet Med Soc. 2018;69(1):815-22.

50.	Seric-Haracic S, Fejzic N, Clanjak-Kudra E, Velic S, Smajlovic M, editors. Mastitis in dairy cow farms in canton Sarajevo and antimicrobial resistance of causative agents. 60th International Meat Industry Conference (MEATCON); 2019 Sep 22-25

2019; Inst Meat Hygiene & Technol, Kopaonik, Serbia; 2019.

51.	Hutu I, Lungu BC, Spataru, II, Torda I, Iancu T, Barrow PA, et al. Microbiological and molecular investigation of antimicrobial resistance in Staphylococcus aureus isolates from western Romanian dairy farms: an epidemiological approach. Animals. 2024;14(15).

52.	Zhu L, Lai Y, Li X, Ma H, Gong F, Sun X, et al. Molecular and epidemiological characterization of Staphylococcus aureus causing bovine mastitis in China. Microb Pathog. 2024;191.

53.	Dobrut A, Siemińska I, Sroka-Oleksiak A, Drożdż K, Sobońska J, Mroczkowska U, et al. Molecular and phenotypic identification of bacterial species isolated from cows with mastitis from three regions of Poland. BMC Vet Res. 2024;20(1).

54.	Liu Y, Xu S, Li M, Zhou M, Huo W, Gao J, et al. Molecular characteristics and antibiotic susceptibility profiles of Mycoplasma bovis associated with mastitis on dairy farms in China. Prev Vet Med. 2020;182:105106.

55.	Wusiman M, Zuo JK, Yu Y, Lv ZY, Wang MD, Nie LH, et al. Molecular characterization of Klebsiella pneumoniae in clinical bovine mastitis in 14 provinces in China. Vet Res Commun. 2025;49(1).

56.	Liu J, Wang X, Bi C, Mehmood K, Ali F, Qin J, et al. Molecular characterization of multi-drug-resistant Staphylococcus aureus in mastitis bovine milk from a dairy farm in Anhui, China. Front Vet Sci. 2022;9.

57.	Farzana Z, Saha A, Siddiki AZ. Molecular characterization of Streptococcus agalactiae and Streptococcus dysgalactiae causing bovine mastitis in the southern region of Bangladesh. J Adv Vet Anim Res. 2023;10(2):178-84.

58.	Tegegne DT, Mamo G, Waktole H, Messele YE. Molecular characterization of virulence factors in Staphylococcus aureus isolated from bovine subclinical mastitis in central Ethiopia. Ann Microbiol. 2021;71(1).

59.	Cai K, Xu M, Liu L, Zhao H. Molecular epidemiology and antimicrobial resistance of Klebsiella pneumoniae strains isolated from dairy cows in Xinjiang, China. Vet Med Sci. 2025;11(1).

60.	Fu S, Wen C, Wang Z, Qiu Y, Zhang Y, Zuo J, et al. Molecular epidemiology and antimicrobial resistance of outbreaks of Klebsiella pneumoniae clinical mastitis in Chinese dairy farms. Microbiol Spectr. 2022;10(6):e0299722.

61.	Song SK, He WJ, Yang DW, Benmouffok M, Wang Y, Li JY, et al. Molecular epidemiology of Klebsiella pneumoniae from clinical bovine mastitis in northern area of China, 2018-2019. Engineering. 2022;10:146-54.

62.	Anderson KL, Lyman R, Moury K, Ray D, Watson DW, Correa MT. Molecular epidemiology of Staphylococcus aureus mastitis in dairy heifers. J Dairy Sci. 2012;95(9):4921-30.

63.	Lin LS, Huang XJ, Yang HF, He YX, He XF, Huang JH, et al. Molecular epidemiology, antimicrobial activity, and virulence gene clustering of Streptococcus agalactiae isolated from dairy cattle with mastitis in China. J Dairy Sci. 2021;104(4):4893-903.

64.	Mora-Hernández Y, Murguía EV, Stinenbosch J, Jauregui PH, van Dijl JM, Buist G. Molecular typing and antimicrobial resistance profiling of 33 mastitis-related Staphylococcus aureus isolates from cows in the Comarca Lagunera region of Mexico. Sci Rep. 2021;11(1).

65.	Han G, Zhang B, Luo Z, Lu B, Luo Z, Zhang J, et al. Molecular typing and prevalence of antibiotic resistance and virulence genes in Streptococcus agalactiae isolated from Chinese dairy cows with clinical mastitis. PLoS One. 2022;17(5).

66.	Fayed H, Elgendy AA, Shoulah SA, Moustafa SM, Maher A, Hikal AF, et al. Mycological and molecular identification of mycoses involved in mastitis from Holstein dairy cattle with special reference to Candida albicans. Vet Res Commun. 2025;49(4).

67.	Zeng JF, Wang Y, Fan LX, Yang N, Pan JW, Han Y, et al. Novel Streptococcus uberis sequence types causing bovine subclinical mastitis in Hainan, China. J Appl Microbiol. 2022;132(3):1666-74.

68.	Camsing A, Phetburom N, Chopjitt P, Pumhirunroj B, Patikae P, Watwiengkam N, et al. Occurrence of antimicrobial-resistant bovine mastitis bacteria in Sakon Nakhon, Thailand. Vet World. 2024;17(6):1202-9.

69.	Rózańska H, Lewtak-Piłat A, Kubajka M, Weiner M. Occurrence of enterococci in mastitic cow's milk and their antimicrobial resistance. J Vet Res. 2019;63(1):93-7.

70.	Sierra TAO, Acosta AC, de Melo RPB, de Oliveira PRF, de Moraes Peixoto R, Cavalcanti EFTSF, et al. Occurrence of extended-spectrum β-lactamase-producing Enterobacteriaceae in raw milk from cows with subclinical mastitis in northeast Brazil. Braz J Microbiol. 2023;54(2):1303-7.

71.	Shahid M, Hussain R, Nawaz Z, Aslam B, Ahmad MZ, Siddique A, et al. Occurrence of virulence genes among methicillin-resistant Staphylococcus aureus isolated from subclinical bovine mastitis. ACS Omega. 2023;8(41):38111-7.

72.	Wang Y, Meng S, Bahetijiang H, Li H, Wang T, Assabayev T, et al. Phenotypic and genotypic characterization of ST103 serotype Ia Streptococcus agalactiae isolated from bovine mastitis in China. BMC Microbiol. 2025;25(1):662.

73.	Xavier A, Almeida AC, Souza CN, Silva LMV, Ruas AXA, Sanglard DA, et al. Phenotypic and genotypic characterization of Staphylococcus aureus isolates in milk from flocks diagnosed with subclinical mastitis. Genet Mol Res. 2017;16(2).

74.	Fazel F, Jamshidi A, Khoramian B. Phenotypic and genotypic study on antimicrobial resistance patterns of E. coli isolates from bovine mastitis. Microb Pathog. 2019;132:355-61.

75.	Abdeen EE, Mousa WS, Abdel-Tawab AA, El-Faramawy R, Abo-Shama UH. Phenotypic, genotypic and antibiogram among Staphylococcus aureus isolated from bovine subclinical mastitis. Pak Vet J. 2021;41(3):289-93.

76.	Tarazi YH, Gharaibeh MH, Al-Hurani HA, Ismail Z. Phylogenetic grouping and antibiogram of ESBL-producing Escherichia coli isolated from bovine mastitis. Trop Anim Sci J. 2023;46(4):410-7.

77.	Archer SC, Bradley AJ, Cooper S, Davies PL, Green MJ. Prediction of Streptococcus uberis clinical mastitis risk using matrix-assisted laser desorption ionization time of flight mass spectrometry (MALDI-TOF MS) in dairy herds. Prev Vet Med. 2017;144:1-6.

78.	Widianingrum DC, Khasanah H, Addy HS. Presence of antibiotic-resistant in Staphylococcal subclinical mastitis in several regencies of east Java, Indonesia. Trop Anim Sci J. 2022;45(1):91-7.

79.	Kateete DP, Kabugo U, Baluku H, Nyakarahuka L, Kyobe S, Okee M, et al. Prevalence and antimicrobial susceptibility patterns of bacteria from milkmen and cows with clinical mastitis in and around Kampala, Uganda. PLoS One. 2013;8(5).

80.	Yu ZN, Wang J, Ho H, Wang YT, Huang SN, Han RW. Prevalence and antimicrobial-resistance phenotypes and genotypes of Escherichia coli isolated from raw milk samples from mastitis cases in four regions of China. J Glob Antimicrob Resist. 2020;22:94-101.

81.	Liang ZY, Shen JH, Liu J, Sun X, Yang YY, Lv YA, et al. Prevalence and characterization of Serratia marcescens isolated from clinical bovine mastitis cases in Ningxia Hui Autonomous Region of China. Infect Drug Resist. 2023;16:2727-35.

82.	Ren Q, Liao GH, Wu ZH, Lv JF, Chen W. Prevalence and characterization of Staphylococcus aureus isolates from subclinical bovine mastitis in southern Xinjiang, China. J Dairy Sci. 2020;103(4):3368-80.

83.	Lakew M, Tolosa T, Tigre W. Prevalence and major bacterial causes of bovine mastitis in Asella, South Eastern Ethiopia. Trop Anim Health Prod. 2009;41(7):1525-30.

84.	Liu J, Liang ZY, Zhongla M, Wang HS, Sun X, Zheng JS, et al. Prevalence and molecular characteristics of Enterococci isolated from clinical bovine mastitis cases in Ningxia. Infect Drug Resist. 2024;17:2121-9.

85.	Chowdhury T, Roy MC, Hossain FMA. Prevalence and zoonotic risk of multidrug-resistant Escherichia coli in bovine subclinical mastitis milk: insights into the virulence and antimicrobial resistance. Food Sci Nutr. 2025;13(1).

86.	Maalaoui A, Majdoub H, Trimeche A, Souissi N, Saidani F, Marnet PG. Prevalence of bovine mastitis and main risk factors in Tunisia. Trop Anim Health Prod. 2021;53(5).

87.	Olivares-Pérez J, Kholif AE, Rojas-Hernández S, Elghandour MM, Salem AZ, Bastida AZ, et al. Prevalence of bovine subclinical mastitis, its etiology and diagnosis of antibiotic resistance of dairy farms in four municipalities of a tropical region of Mexico. Trop Anim Health Prod. 2015;47(8):1497-504.

88.	Ewida RM, Al-Hosary AAT. Prevalence of enterotoxins and other virulence genes of Staphylococcus aureus caused subclinical mastitis in dairy cows. Vet World. 2020;13(6):1193-8.

89.	Xu TL, Cao WD, Huang YC, Zhao JW, Wu XY, Yang ZP. The prevalence of Escherichia coli derived from bovine clinical mastitis and distribution of resistance to antimicrobials in part of Jiangsu province, China. Agriculture-Basel. 2023;13(1).

90.	Yang FL, Shen C, He BX, Yang YY, Gong dC, Li XS. The prevalence of heifer mastitis and its associated risk factors in Huanggang, Central China. Trop Anim Health Prod. 2015;47(1):87-92.

91.	Shrestha A, Bhattarai RK, Luitel H, Karki S, Basnet HB. Prevalence of methicillin-resistant Staphylococcus aureus and pattern of antimicrobial resistance in mastitis milk of cattle in Chitwan, Nepal. BMC Vet Res. 2021;17(1):239.

92.	Song XB, Huang XP, Xu HY, Zhang C, Chen S, Liu F, et al. The prevalence of pathogens causing bovine mastitis and their associated risk factors in 15 large dairy farms in China: an observational study. Vet Microbiol. 2020;247.

93.	Xu JY, Yang ZQ, Xing J, Yang JT, Zang RX, Bai JL, et al. Prevalence of the "high-pathogenicity island" in escherichia coli isolated from clinical and subclinical bovine mastitis in China. Asian J Anim Vet Adv. 2012;7(10):980-8.

94.	Smistad M, Bakka HC, Sølverød L, Jørgensen HJ, Wolff C. Prevalence of udder pathogens in milk samples from Norwegian dairy cows recorded in a national database in 2019 and 2020. Acta Vet Scand. 2023;65(1).

95.	Wu XH, Hou SB, Zhang QW, Ma YJ, Zhang Y, Kan W, et al. Prevalence of virulence and resistance to antibiotics in pathogenic enterococci isolated from mastitic cows. J Vet Med Sci. 2016;78(11):1663-8.

96.	Saeed SI, Mat Yazid KA, Hashimy HA, Dzulkifli SK, Nordin F, Nik Him NA, et al. Prevalence, antimicrobial resistance, and characterization of Staphylococcus aureus isolated from subclinical bovine mastitis in east coast Malaysia. Animals. 2022;12(13).

97.	Seyoum B, Kefyalew H, Abera B, Abdela N. Prevalence, risk factors and antimicrobial susceptibility test of Staphylococcus aureus in bovine cross breed mastitic milk in and around Asella town, Oromia regional state, southern Ethiopia. Acta Trop. 2018;177:32-6.

98.	Farabi A, Hossain H, Brishty KA, Rahman MH, Rahman M, Siddiqui MSI, et al. Prevalence, risk factors, and antimicrobial resistance of Staphylococcus and Streptococcus species isolated from subclinical bovine mastitis. Foodborne Pathog Dis. 2025;22(7):467-76.

99.	Xie XM, Pan ZH, Yu Y, Yu LR, Wu F, Dong J, et al. prevalence, virulence, and antibiotics gene profiles in Lactococcus garvieae Isolated from cows with clinical mastitis in China. Microorganisms. 2023;11(2).

100.	Breen JE, Green MJ, Bradley AJ. Quarter and cow risk factors associated with the occurrence of clinical mastitis in dairy cows in the United Kingdom. J Dairy Sci. 2009;92(6):2551-61.

101.	Elhaig MM, El-Tarabili RM, Wahdan A. A report of coagulase-negative Staphylococci from clinically incurable cases of bovine mastitis: prevalence, biofilm formation, and resistance profile. Iran J Vet Res. 2024;25(3).

102.	Kálmán D, Jánosi S, Egyed L. Role of bovine herpesvirus 4 in bacterial bovine mastitis. Microb Pathog. 2004;37(3):125-9.

103.	Krebs I, Zhang Y, Wente N, Leimbach S, Krömker V. Severity of clinical mastitis and bacterial shedding. Pathogens. 2023;12(9).

104.	Jamali H, Radmehr B, Ismail S. Short communication: prevalence and antibiotic resistance of Staphylococcus aureus isolated from bovine clinical mastitis. J Dairy Sci. 2014;97(4):2226-30.

105.	Dyson R, Charman N, Hodge A, Rowe SM, Taylor LF. A survey of mastitis pathogens including antimicrobial susceptibility in southeastern Australian dairy herds. J Dairy Sci. 2022;105(2):1504-18.

106.	Bruno DR, Tonooka KH, Lehenbauer TW, Aly SS, Elashmawy WR. Annual and seasonal trends in mastitis pathogens isolated from milk samples from dairy cows of california's san joaquin valley dairies between january 2009 and december 2023. Vet Sci. 2025;12(7).

107.	Khasapane NG, Nkhebenyane JS, Kwenda S, Khumalo ZTH, Mtshali PS, Taioe MO, et al. Application of culture, PCR, and PacBio sequencing for determination of microbial composition of milk from subclinical mastitis dairy cows of smallholder farms. Open Life Sci. 2021;16(1):800-8.

108.	Paradis MÈ, Haine D, Gillespie B, Oliver SP, Messier S, Comeau J, et al. Bayesian estimation of the diagnostic accuracy of a multiplex real-time PCR assay and bacteriological culture for 4 common bovine intramammary pathogens. J Dairy Sci. 2012;95(11):6436-48.

109.	Belay N, Mohammed N, Seyoum W. bovine mastitis: prevalence, risk factors, and bacterial pathogens isolated in lactating cows in Gamo Zone, southern Ethiopia. Vet Med. 2022;13:9-19.

110.	Ara J, Chowdhury S, Nath C, Barua S, Nizami TA, Islam MS, et al. Burden of multidrug-resistant S. aureus and E. coli isolated from bovine milk in Chattogram Metropolitan Area, Bangladesh. Microbe. 2025;8.

111.	Sharma V, Sharma S, Dahiya DK, Khan A, Mathur M, Sharma A. Coagulase gene polymorphism, enterotoxigenecity, biofilm production, and antibiotic resistance in Staphylococcus aureus isolated from bovine raw milk in north West India. Ann Clin Microbiol Antimicrob. 2017;16.

112.	Lin YS, Han JE, Barkema HW, Wang Y, Gao J, Kastelic JP, et al. Comparative genomic analyses of Lactococcus garvieae isolated from bovine mastitis in China. Microbiol Spectr. 2023;11(3).

113.	Xu SY, Liu Y, Gao J, Zhou M, Yang JY, He FM, et al. Comparative genomic analysis of Streptococcus dysgalactiae subspecies dysgalactiae isolated from bovine mastitis in China. Front Microbiol. 2021;12.

114.	Nagahata H, Ito H, Maruta H, Nishikawa Y, Susukino H, Matsuki S, et al. Controlling highly prevalent Staphylococcus aureus mastitis from the dairy farm. J Vet Med Sci. 2007;69(9):893-8.

115.	Marashifard M, Aliabad ZK, Hosseini S, Darban-Sarokhalil D, Mirzaii M, Khoramrooz SS. Determination of antibiotic resistance pattern and virulence genes in Escherichia coli isolated from bovine with subclinical mastitis in southwest of Iran. Trop Anim Health Prod. 2019;51(3):575-80.

116.	Aqib AI, Ijaz M, Farooqi SH, Ahmed R, Shoaib M, Ali MM, et al. Emerging discrepancies in conventional and molecular epidemiology of methicillin resistant Staphylococcus aureus isolated from bovine milk. Microb Pathog. 2018;116:38-43.

117.	Zaatout N, Ayachi A, Kecha M. Epidemiological investigation of subclinical bovine mastitis in Algeria and molecular characterization of biofilm-forming Staphylococcus aureus. Trop Anim Health Prod. 2020;52(1):283-92.

118.	Al-Farha A, Hemmatzadeh F, Khazandi M, Hoare A, Petrovski K. Evaluation of effects of Mycoplasma mastitis on milk composition in dairy cattle from South Australia. BMC Vet Res. 2017;13.

119.	Girmay W, Gugsa G, Taddele H, Tsegaye Y, Awol N, Ahmed M, et al. Isolation and identification of methicillin-resistant Staphylococcus aureus (MRSA) from milk in shire dairy farms, Tigray, Ethiopia. Vet Med Int. 2020;2020.

120.	Zhao H, Guo T, Zhou Y, Zhao F, Sun Y, Wang Y, et al. Major causative bacteria of dairy cow mastitis in the Inner Mongolia Autonomous Region, China, 2015-2024: an epidemiologic survey and analysis. Vet Sci. 2025;12(3).

121.	Fesseha H, Mathewos M, Aliye S, Wolde A. Study on prevalence of bovine mastitis and associated risk factors in dairy farms of Modjo town and suburbs, central Oromia, Ethiopia. Vet Med. 2021;12:271-83.

122.	Ahmed HF, Straubinger RK, Hegazy YM, Ibrahim S. Subclinical mastitis in dairy cattle and buffaloes among small holders in Egypt: prevalence and evidence of virulence of Escherichia coli causative agent. Trop Biomed. 2018;35(2):321-9.

123.	Ranasinghe RMSBK, Deshapriya RMC, Abeygunawardana DI, Rahularaj R, Dematawewa CMB. Subclinical mastitis in dairy cows in major milk-producing areas of Sri Lanka: Prevalence, associated risk factors, and effects on reproduction. J Dairy Sci. 2021;104(12):12900-11.

124.	Wang L, Haq SU, Shoaib M, He J, Guo W, Wei X, et al. Subclinical mastitis in small-holder dairy herds of Gansu province, northwest China: prevalence, bacterial pathogens, antimicrobial susceptibility, and risk factor analysis. Microorganisms. 2024;12(12).

125.	Ksouri S, Djebir S, Hadef Y, Benakhla A. Survey of bovine mycotic mastitis in different mammary gland statuses in two north-eastern regions of Algeria. Mycopathologia. 2015;179(3-4):327-31.

126.	Anwar MA, Aziz S, Ashfaq K, Aqib AI, Shoaib M, Naseer MA, et al. Trends in frequency, potential risks and antibiogram of E. coli isolated from semi-intensive dairy systems. Pak Vet J. 2022;42(2):167-72.

127.	Bochniarz M, Piech T, Kocki T, Iskra M, Krukowski H, Jagielski T. Tryptophan, kynurenine and kynurenic acid concentrations in milk and serum of dairy cows with prototheca mastitis. Animals. 2021;11(12).

128.	Naqvi SA, De Buck J, Dufour S, Barkema HW. Udder health in Canadian dairy heifers during early lactation. J Dairy Sci. 2018;101(4):3233-47.

129.	Shahzad MA, Yousaf A, Ahsan A, Irshad H, Riaz A, Khan A, et al. Virulence and resistance profiling of Staphylococcus aureus isolated from subclinical bovine mastitis in the Pakistani Pothohar region. Sci Rep. 2024;14(1).

130.	Bag MAS, Khan MSR, Sami MDH, Begum F, Islam MS, Rahman MM, et al. Virulence determinants and antimicrobial resistance of E. coli isolated from bovine clinical mastitis in some selected dairy farms of Bangladesh. Saudi J Biol Sci. 2021;28(11):6317-23.

131.	Eidaroos NH, Algammal AM, Mohamaden WI, Alenzi AM, Alghamdi S, Kabrah A, et al. Virulence traits, agr typing, multidrug resistance patterns, and biofilm ability of MDR Staphylococcus aureus recovered from clinical and subclinical mastitis in dairy cows. BMC Microbiol. 2025;25(1).

132.	Boonyayatra S, Tharavichitkul P, Oliver SP. Virulence-associated genes and molecular typing of Streptococcus uberis associated with bovine mastitis in northern Thailand. Turk J Vet Anim Sci. 2018;42(1):73-81.

133.	Timonen AAE, Katholm J, Petersen A, Mõtus K, Kalmus P. Within-herd prevalence of intramammary infection caused by Mycoplasma bovis and associations between cow udder health, milk yield, and composition. J Dairy Sci. 2017;100(8):6554-61.

134.	Khan A, Durrani AZ, Yousaf A, Khan JA, Chaudhry M, Fatima Z, et al. Epidemiology and antimicrobial susceptibility of methicillin-resistant Staphylococcus aureus in cattle of Pothohar Region, Pakistan. Pak Vet J. 2019;39(3):438-42.

135.	Gangwal A, Kashyap SK, Katiyar S, Meena D, Boyal P, Gupta K. Isolation and identification of common mastitis causing pathogens from clinical bovine mastitic milk. J Pure Appl Microbiol. 2017;11(1):329-33.

136.	Neamah AA, Fahed KH, Sadeq JN, Alfatlawi MA. Molecular characterization and phylogenetic analysis of Escherichia coli isolated from milk of cattle affected by mastitis. Iraqi J Vet Sci. 2022;36(1):251-4.

137.	Ahmed A, Ijaz M, Khan JA, Anjum AA. Molecular characterization and therapeutic insights into biofilm positive Staphylococcus aureus isolated from bovine subclinical mastitis. Pak Vet J. 2022;42(4):584-90.
